# An efficient metal-free and catalyst-free C–S/C–O bond-formation strategy: synthesis of pyrazole-conjugated thioamides and amides

**DOI:** 10.3762/bjoc.19.22

**Published:** 2023-03-02

**Authors:** Shubham Sharma, Dharmender Singh, Sunit Kumar, Vaishali, Rahul Jamra, Naveen Banyal, Deepika, Chandi C Malakar, Virender Singh

**Affiliations:** 1 Department of Chemistry, Dr B R Ambedkar National Institute of Technology (NIT) Jalandhar, 144027, Punjab, India; 2 Central Revenues Control Laboratory, New Delhi-110012, India; 3 Department of Chemistry, Central University of Punjab, Bathinda, 151401, Punjab, India; 4 Department of Chemistry, National Institute of Technology (NIT) Manipur, Imphal, 795004, India

**Keywords:** C–S/O bond formation, metal-free, oxidative amidation, pyrazole carbaldehydes, sulfur insertion, thioamides

## Abstract

An operationally simple and metal-free approach is described for the synthesis of pyrazole-tethered thioamide and amide conjugates. The thioamides were generated by employing a three-component reaction of diverse pyrazole C-3/4/5 carbaldehydes, secondary amines, and elemental sulfur in a single synthetic operation. The advantages of this developed protocol refer to the broad substrate scope, metal-free and easy to perform reaction conditions. Moreover, the pyrazole C-3/5-linked amide conjugates were also synthesized via an oxidative amination of pyrazole carbaldehydes and 2-aminopyridines using hydrogen peroxide as an oxidant.

## Introduction

**Figure 1:**
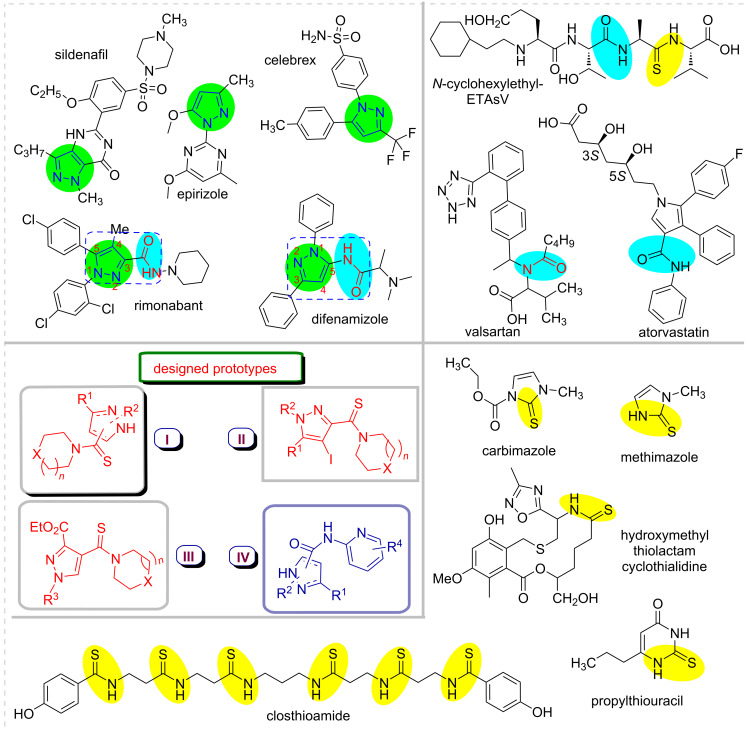
Representative drug molecules based on pyrazole, thioamide, and amide derivatives.

During the past years, the significance of pyrazole chemistry has been notably escalated which is attributed to the discovery of their amazing biological properties. Among the heterocyclic molecules, pyrazoles are considered as privileged scaffolds for the design and construction of pharmacologically relevant compounds [1–3]. Their effectiveness has been witnessed in agrochemicals [4–6], chemicals, and pharmaceutical industries. Moreover, recent findings have affirmed the potential of the pyrazole nucleus as CB1 receptor antagonists [7], estrogen receptor ligands [8], A2A receptor antagonists [9], and DNA intercalating agents [10]. Importantly, pyrazole derivatives can be traced in a spectrum of well-established drug candidates of various categories with diverse therapeutic properties such as antipyretic [11], antibacterial [12], anticancer [13–15], antiviral [16], analgesic [17], antioxidants, antimicrobial [18], antidiabetic, anticonvulsant [19], antihelminthic [20], and antiarrhythmic activities. The pyrazole nucleus is a core unit in several FDA-approved marketed drugs such as sildenafil [21–23], celebrex [24–25], difenamizole [26], epirizole [27], rimonabant [28] etc. (Figure 1). Additionally, pyrazole derivatives hold a prominent position in the field of materials science as a result of their numerous applications in products like brightening agents [29], semiconductors [30], and organic light-emitting diodes [31]. Substituted pyrazoles are also of considerable interest because of their synthetic utility as chiral auxiliaries [32], synthetic reagents in multicomponent reactions [33–34], and guanylating agents [35].

The installation of a thioamide functionality has attracted an immense attention in medicinal chemistry, due to various biological activities [36–39]. Accordingly, a broad spectrum of effective and useful methods has been acknowledged in the literature for their preparation [40–42]. In this regard, a review article by Jagodzinski et al. based on the examination of a massive virtual library synthesized with frequently occurring pharmacophores originating from drug components comes to the conclusion that the thioamide linkage establishes an intriguing class of biologically significant compounds amenable to combinatorial chemistry [43]. This organic functional group is found in vital biological and pharmaceutical molecules such as *N-*cyclohexylethyl-ETAsV [44], carbimazole, methimazole, propylthiouracil [45], and closthioamide [46] (Figure 1). Moreover, they also find widespread applications as intermediates for the construction of five- and six-membered heterocyclic compounds [47] and active pharmaceutical ingredients [48] such as fenclosic acid, fentiazac, and febuxostate.

Similarly, in contemporary chemistry, the amide functionality is one of the most studied functional groups. Specifically, this moiety is vital for the formation of the backbone of structural proteins and enzymes [49]. The amide linkage is present in several naturally occurring compounds and it is also one of the most productive functional groups in current pharmaceutical drugs [50–51]. As prime examples; atorvastatin [52], valsartan [53] and *N*-cyclohexylethyl-ETAsV are successfully utilized to treat various life challenging diseases (Figure 1). Accordingly, as a part of our ongoing research project, it was planned to incorporate thioamide and amide functional groups into a pyrazole framework to develop new scaffolds.

**Figure 2:**
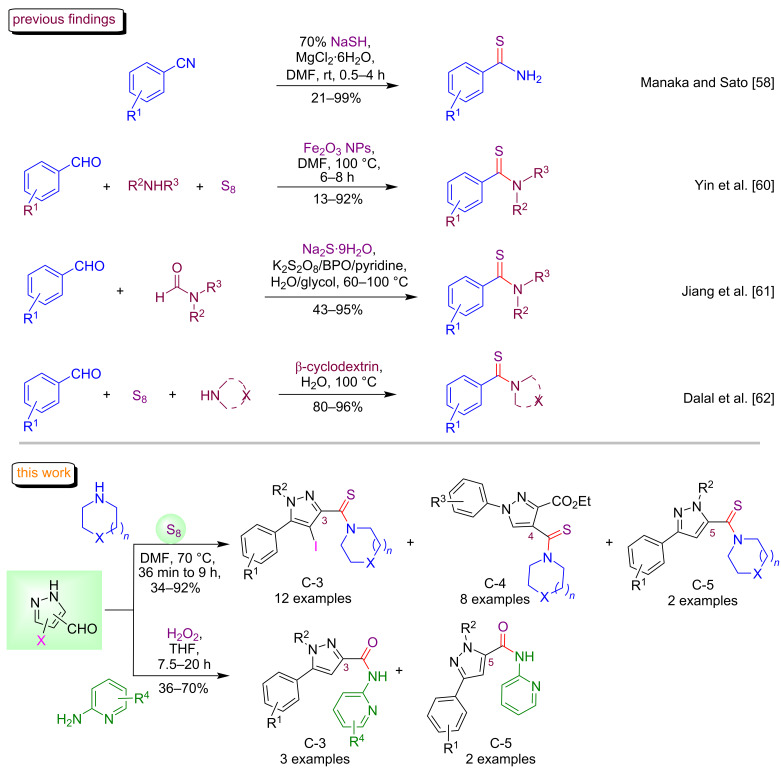
Previous and present findings for the synthesis of thioamide derivatives.

An extensive literature survey revealed that several approaches are well-documented for the construction of the thioamide functionality including base-catalyzed Willgerodt–Kindler reaction [54], Kindler reaction in the presence of sulfated tungstate [55], thionation of amides using thionating reagents [56] and thionation of amides using TsCl (4-toluenesulfonyl chloride) or PSCl_3_-mediated Beckmann rearrangement [57]. Although, these protocols are useful and have exhibited wide applications in organic synthesis (Figure 2), the scope of these reported methods may suffer from drawbacks such as harsh reaction conditions, use of expensive reagents, prolonged reaction times, low product yields, and cumbersome product isolation procedures [58–62]. In the recent past, our group also reported two methods towards the exploration of elemental sulfur for the formation of a sulfur-containing framework; however, these methods suffer from some drawbacks such as lack of diversity in starting substrate, need of base/catalyst and limitation of starting reagents [63–64]. Our current work was completed with the exploration of the position of the pyrazole ring like C-3, C-4 and C-5 and we also employed the pyrazole-based AXB3s (4-iodo-C-3 and 4-iodo-C-5). Moreover, we also disclosed the synthesis of pyrazole C-3/C-5 amide conjugates.

Fascinated by the immense pharmacological profiles of pyrazole, thioamide and amide derivatives, it was envisaged to develop a practical approach towards the synthesis of pyrazole-thioamide and pyrazole-amide conjugates. Elemental sulfur was explored as a sulfurating reagent for the generation of thioamides owing to its nontoxic, odorless nature and versatile reactivity profile [65–76]. To the best of our knowledge, the syntheses of pyrazole C-3/4/5-linked thioamide and amide conjugates have not been reported. Herein, we report an operationally simple one-pot procedure for the preparation of highly diversified thioamide and amide-linked pyrazole analogues.

## Results and Discussion

Initially, the synthesis of pyrazole C-3/4/5 carbaldehydes and 4-iodopyrazole-3-carbaldehydes was achieved by employing the recently reported procedures [77–80]. Thereafter, the pyrazole-3-carbaldehyde **1**, morpholine (**C**) and elemental sulfur were selected as the model substrates towards the preparation of pyrazole-linked thioamide derivatives. To begin with, an experiment was executed with model reactants in the presence of catalytic amounts of β-cyclodextrin (β-CD) [81] under aqueous conditions at room temperature as well as under heating at 100 °C (entries 1 and 2, Table 1). Unfortunately, the model reactants remained unreacted and similar observations were recorded using a mixture of H_2_O/MeOH 1:4, and methanol as a reaction medium (entries 3 and 4, Table 1). Moreover, it was also investigated that various organic solvents in combination with β-CD at room temperature were inactive towards accomplishment of this transformation (entries 5–8, Table 1). Fortunately, when the reaction was performed in CH_3_CN at 60 °C; a polar product was obtained, which was isolated after a short silica gel column chromatography (entry 9, Table 1). To our delight, the spectroscopic analysis revealed the structure of the purified product as (5-(4-fluorophenyl)-1-phenyl-1*H*-pyrazol-3-yl)(morpholino)methanethione (**1C**), which was obtained in 64% isolated yield. Encouraged by these preliminary results, we next assembled the model reactants in DMF as a solvent in the presence of β-CD at 60 °C. It was learned that the outcome of the reaction was slightly better (reaction time was reduced and the yield of the product **1C** was increased to 70%, entry 10, Table 1), which indicated the superiority of DMF over other solvents. Subsequently, we examined the effects of La(OTf)_3_ as a catalyst in combination with DMF as a solvent. However, the targeted prototype **1C** was obtained only in 20% yield at 60 °C after 24 hours of reaction time (entries 11 and 12, Table 1). Next, ZnO nanoparticles were screened for the thioamidation of pyrazole-3-carbaldehyde. The desired thioamide-conjugated pyrazole **1C** was afforded in 30% yield, as the starting substrates were not completely consumed even after 24 hours of reaction time (entry 13, Table 1). On the basis of the experimental results above, we concluded that CH_3_CN and DMF were the ideal solvents for this transformation towards the effective formation of the product. As per literature reports, K_2_CO_3_ shows remarkable efficacy in various organic transformations [82]. Hence, this reaction was also examined under the influences of K_2_CO_3_ (2 equiv) in CH_3_CN, but the reaction conditions were inactive towards the formation of pyrazole-tethered thioamide **1C** (entry 14, Table 1). Surprisingly, when the reaction was carried out in DMF at ambient temperature, the desired product **1C** was obtained in 80% yield (entry 15, Table 1). However, the same reaction under heating conditions at 70 °C, afforded the desired product **1C** in 82% yield with a drastic reduction in the reaction time to 1 hour (entry 16, Table 1). Moreover, an increase in the amount of base had a negligible effect on the yield of the thioamide conjugate **1C** (entries 17 and 18, Table 1). To check the role of K_2_CO_3_, we executed a model reaction in DMF without base (K_2_CO_3_) and it was noted that pyrazole-linked thioamide **1C** was obtained in excellent yield (90%) after 2 hours of reaction time (entry 19, Table 1). This experiment indicated that the K_2_CO_3_ was not mandatory for the desired thioamidation reaction. After that, DMSO and NMP were also screened as solvents in the absence of base, but a longer reaction time was required for similar transformation (7 h) (entries 20 and 21, Table 1). A reaction of model substrates under neat conditions delivered product **1C** in poor yield (entry 22, Table 1). Based on these screening experiments, it was concluded that the reaction proceeded smoothly in DMF as the reaction medium at 70 °C for 2 hours, and these were considered as the optimal conditions for further investigation of the scope of the developed strategy (entry 19, Table 1).

**Table 1:** Screening of reaction conditions towards the formation of pyrazole-conjugated thioamide.^a^

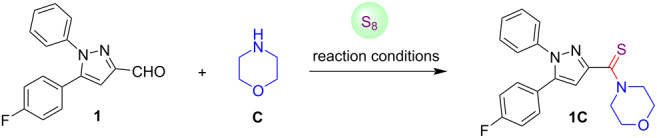					

Entry	Catalyst/reagent (equiv)	solvent^b^	temp. (^o^C)	time (h)	isolated yield^c,d^

1	β-CD (0.2)	H_2_O	rt	7	NR^e^
2	β-CD (0.2)	H_2_O	100	7	NR
3	β-CD (0.2)	MeOH	rt	7	NR
4	β-CD (0.2)	H_2_O/MeOH 1:4	rt	7	NR
5	β-CD (0.2)	DCE	rt	7	NR
6	β-CD (0.2)	AcOH	rt	3	NR
7	β-CD (0.2)	CH_3_CN	rt	3	NR
8	β-CD (0.2)	toluene	rt	3	NR
9	β-CD (0.2)	CH_3_CN	60	7	64%
10	β-CD (0.2)	DMF	60	3	70%
11	La(OTf)_3_ (0.1)	DMF	rt	24	NR
12	La(OTf)_3_ (0.1)	DMF	60	24	20%
13	ZnO NPs (0.1)	DMF	rt	24	30% + **1**
14	K_2_CO_3_ (2.0)	CH_3_CN	rt	18	NR
15	K_2_CO_3_ (1.0)	DMF	rt	24	80%
16	K_2_CO_3_ (1.0)	DMF	70	1	82%
17	K_2_CO_3_ (2.0)	DMF	70	1	80%
18	K_2_CO_3_ (3.0)	DMF	70	1	79%
**19**	–	**DMF**	**70**	**2**	**90%**
20	–	DMSO	70	7	88%
21	–	NMP	70	7	85%
22	–	neat	70	29	13% + **1**

^a^All reactions were optimized with 0.07 mmol (1 equiv) of **1**, 0.08 mmol (1.1 equiv) of **C**, 0.28 mmol (4 equiv) of sulfur in 2 mL of solvent; ^b^all reactions were performed in anhydrous solvents (except entries 1, 2, 4, and 22); ^c^isolated yields of the purified product **1C**; ^d^NR = no reaction; ^e^the model substrates remained intact.

**Scheme 1:**
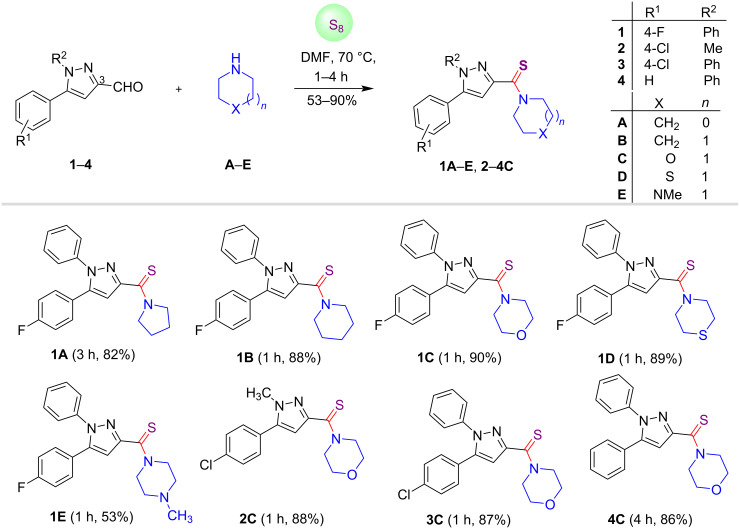
Synthesis of pyrazole C-3-tethered thioamides.

Having established the optimal reaction conditions, we explored the generality and the scope of this metal- and catalyst-free approach by employing pyrazole C-3 carbaldehydes **1**–**4**, secondary amines **A**–**E** and elemental sulfur as substrates. It was observed that the reaction conditions were compatible with different pyrazole-3-carbaldehydes and various secondary amines for the synthesis of pyrazole C-3-tethered thioamides **1A**–**E** and **2**–**4C** with the yield ranging from 53–90% (Scheme 1). Notably, 1-methylpiperazine (**E**) afforded the product in low yield (53%). The electronic nature of the substituents located at the N-1 and C-5 positions of the pyrazole ring exerted unnoticeable impacts on the yields of the desired products.

**Scheme 2:**
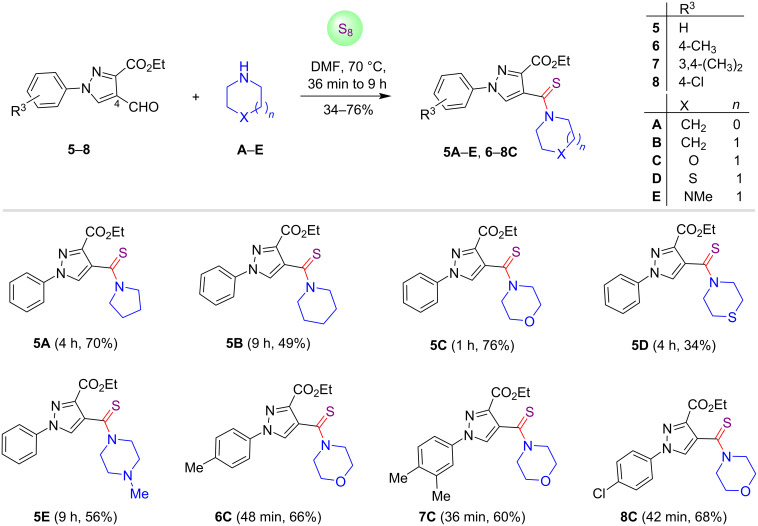
Synthesis of pyrazole C-4-tethered thioamides.

Encouraged by these successful results, we further investigated the thioamidation reaction of various pyrazole-4-carbaldehydes **5**–**8** using the optimal conditions as illustrated in Scheme 2. The pyrazole-4-carbaldehydes **5**–**8** were found to be suitable substrates for this operation. It is pertinent to mention that the substrate **5** reacted with cyclic secondary amines **A**–**C** to yield the designed prototypes in moderate to good yields (49–76%), whereas thiomorpholine (**D**) delivered the thioamide conjugate **5D** in low yield (34%). During the preparation of pyrazole C-4-conjugated thioamides **5A**–**E** and **6**–**8C**, it was also noticed that when the reaction was exercised with morpholine (**C**), the reaction was accomplished in lesser time (36 min to 1 h) as compared to other secondary amines.

**Scheme 3:**
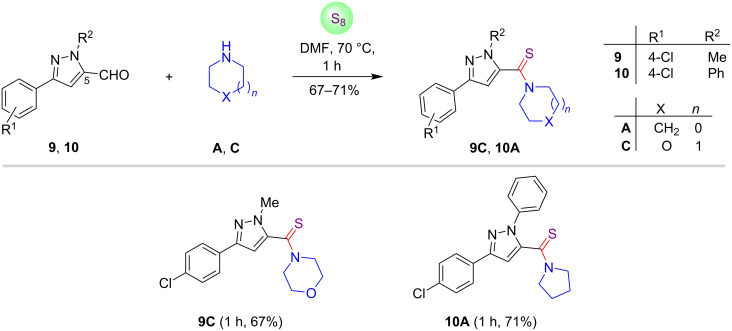
Metal- and catalyst-free preparation of pyrazole C-5-linked thioamide conjugates.

To further validate the synthetic flexibility of this methodology, we employed pyrazole C-5 carbaldehydes **9** and **10** for the synthesis of thioamide conjugates. It was noticed that the pyrazole-5-carbaldehydes **9** and **10** were more reactive as compared to pyrazole C-3 and C-4 carbaldehydes, leading to the formation of products **9C** and **10A** in high yields (67–71%) within 1 hour of reaction time as depicted in Scheme 3.

**Scheme 4:**
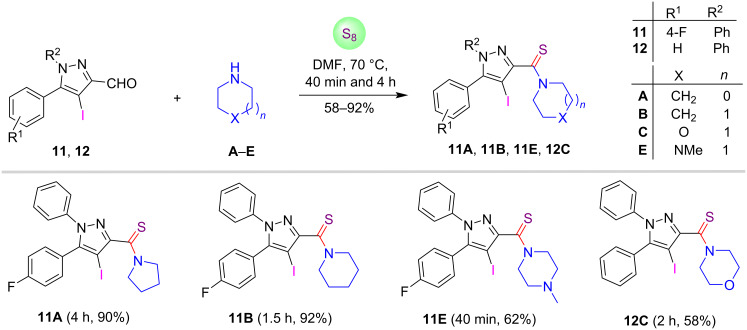
Synthesis of 4-iodopyrazole C-3-tethered thioamides.

Thereafter, the substrates 4-iodopyrazole-3-carbaldehydes were further investigated for this metal- and catalyst-free sulfur insertion reaction as shown in Scheme 4. It was found that 4-iodopyrazole C-3 carbaldehydes **11** and **12** were also tolerated well for this thioamidation process and furnished the anticipated products **11A**,**B**,**E**, and **12C** in good to excellent yields (58–92%) within 40 min to 4 hours.

**Scheme 5:**
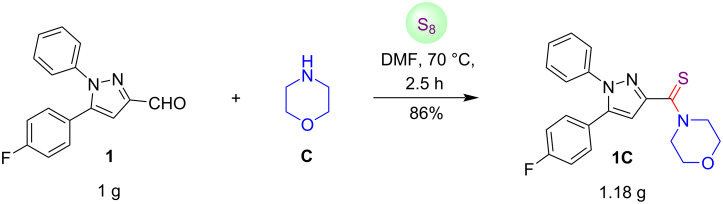
Gram-scale scope of the current protocol.

To check the industrial scope of the current protocol, we conducted a gram-scale reaction between pyrazole-3-carbaldehyde **1**, morpholine (**C**) and elemental sulfur under the standard reaction conditions as depicted in Scheme 5. It was noticed that this one-pot operation was completed within 2.5 hours and smoothly furnished the desired product, (5-(4-fluorophenyl)-1-phenyl-1*H*-pyrazol-3-yl)(morpholino)methanethione (**1C**) in 86% yield.

**Scheme 6:**
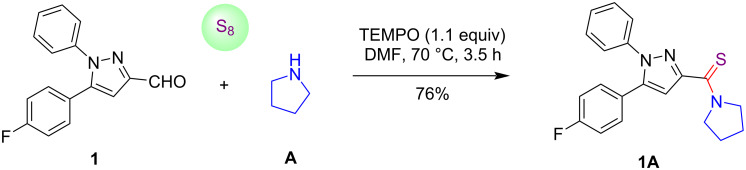
Control experiment.

To find out more information about the mechanistic route of the reaction, we performed a control experiment in the presence of TEMPO as a radical scavenger as depicted in Scheme 6. The reaction of pyrazole-3-carbaldehyde **1**, pyrrolidine (**A**) and elemental sulfur in the presence of 1.1 equiv of TEMPO delivered the targeted product in 76% yield. On the basis of this experiment, it was concluded that TEMPO did not affect the progress of the reaction and the formation of product **1A**. Hence, a radical mechanism of the reaction may be ruled out.

The successful synthesis of pyrazole C-3/4/5-tethered thioamides inspired us to generate analogous pyrazole-pyridine conjugates having an amide linkage. For this purpose, 5-(4-fluorophenyl)-1-phenyl-1*H*-pyrazole-3-carbaldehyde (**1**) and 2-aminopyridine (**F**) were selected as the model reactants to explore this transformation. Initially, we conducted an oxidative amidation reaction of pyrazole-3-carbaldehyde **1** and 2-aminopyridine (**F**) in the presence of TBHP in DMSO as a solvent at 130 °C (entry 1, Table 2). However, the reaction required longer time (20 h) for the completion, and afforded a product in 29% yield only. It was realized that the isolated product was the desired product, 5-(4-fluorophenyl)-1-phenyl-*N*-(pyridin-2-yl)-1*H*-pyrazole-3-carboxamide (**1F**), as analyzed by spectroscopic data.

**Table 2:** Optimization of the reaction conditions towards the formation of pyrazole-pyridine conjugates having an amide linkage.^a^

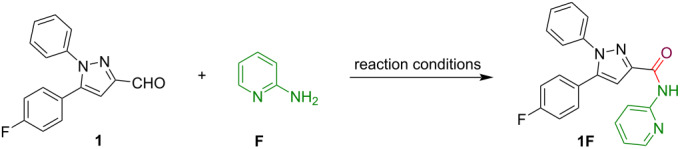					

Entry	Oxidant (equiv)	Solvent^b^	Temp. (°C)	Time (h)	Isolated yield^c^

1	TBHP (3.0)	DMSO	130	20	29%
2	TBHP (3.0)	DMF (10.0 equiv)	130	10	35% + **1**
3	TBHP (3.0)	CH_3_CN (10.0 equiv)	100	10	37% + **1**
4	TBHP (3.0)	THF (10.0 equiv)	100	9	42% + **1**
5	TBHP (3.0)	MeOH (10.0 equiv)	80	8	40% + **1**
6	TBHP (10.0)	DMSO (2.0 equiv)	70	18	36% + **1**
7	H_2_O_2_ (25.0)	neat	rt	19	30%
8	H_2_O_2_ (5.0)	DMSO	70	19	10% + **1**
9	H_2_O_2_ (25.0)	DMSO (2.0 equiv)	70	5	40%
10	H_2_O_2_ (10.0)	DMSO (2.0 equiv)	70	4	50%
11	H_2_O_2_ (10.0)	DMF (2.0 equiv)	70	8	33% + **1**
12	H_2_O_2_ (10.0)	CH_3_CN (2.0 equiv)	70	5	56%
13	H_2_O_2_ (10.0)	THF (2.0 equiv)	70	7	58%
14	H_2_O_2_ (10.0)	MeOH (2.0 equiv)	70	6	45%
15	H_2_O_2_ (10.0)	CH_3_CN	70	4	54%
**16**	**H** **_2_** **O** **_2_** ** (10.0)**	**THF**	**70**	**4**	**61%**

^a^All the optimization reactions were conducted with 0.07 mmol (1.0 equiv) of **1**, 0.08 mmol (1.1 equiv) of **F**; ^b^all the reactions were examined in dry solvents (except entry 7); ^c^isolated yields of **1F**.

Next, we screened other organic solvents including DMF, CH_3_CN, THF, and MeOH to improve the yield of the desired product **1F**, but only a slight improvement in the yield was observed (entries 2–5, Table 2). The oxidant TBHP (10 equiv) failed to deliver the anticipated product in good yield (36%, entry 6, Table 2). Similar results were obtained with H_2_O_2_ (25.0 equiv) under neat reaction conditions (entry 7, Table 2). Next, we performed the oxidative amidation reaction with 5.0 equiv of H_2_O_2_ in DMSO as the reaction medium under heating, whereas, a poor yield of the product was obtained (entry 8, Table 2). Moreover, different combinations of H_2_O_2_ and DMSO were examined for the oxidative amidation of pyrazole-3-carbaldehyde **1** (entries 9 and 10, Table 2). Interestingly, a significant reduction in the reaction time was detected with 25 equiv as well as 10 equiv of H_2_O_2_. Next, we screened DMF, CH_3_CN, THF, and MeOH (2.0 equiv) with 10.0 equiv of H_2_O_2_ to increase the yield of the designed prototype **1F**. An acceptable enhancement was observed in the yield of the desired compound (58%) **1F** (entries 11–14, Table 2). After that, we subjected all the starting substrates to an excess amount of CH_3_CN and THF as reaction solvents (entries 15 and 16, Table 2). It was noticed from these two experiments that THF was the outstanding solvent for our current transformation (entry 16, Table 2). From the above screening experiments, it was concluded that 10.0 equiv of hydrogen peroxide in THF at 70 °C proved to be the optimal conditions for the construction of the pyrazole-pyridine conjugate with an amide linkage (entry 16, Table 2).

**Scheme 7:**
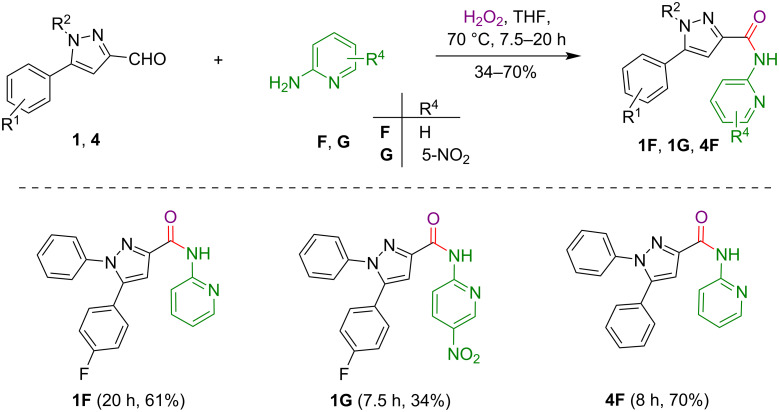
H_2_O_2_-mediated synthesis of pyrazole-pyridine conjugates with amide tethers.

Having the optimized conditions in hand, we employed pyrazole-3-carbaldehydes **1** and **4** for the reaction with different 2-aminopyridines **F** and **G** towards the preparation of amide tethers as displayed in Scheme 7. The pyrazole-3-carbaldehydes **1** and **4** reacted efficiently with 2-aminopyridine (**F**) to deliver the pyrazole conjugated amides **1F** and **4F** in good yields (61 and 70%), whereas, in the case of 5-nitro-2-aminopyridine (**G**), the anticipated product **1G** was obtained in low yield (34%).

**Scheme 8:**
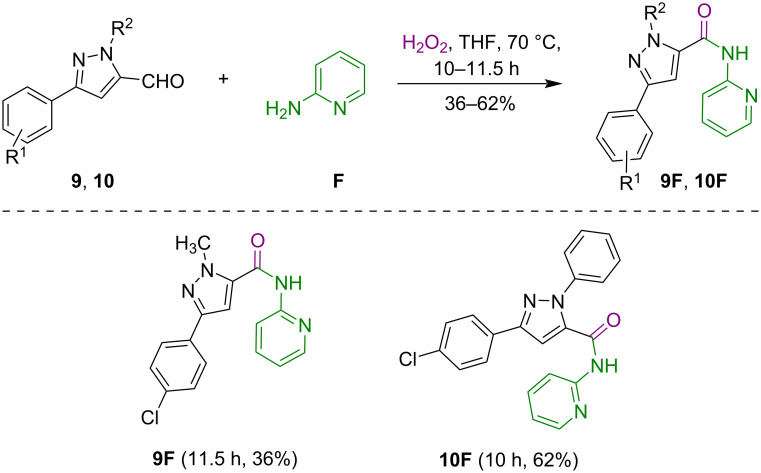
Synthesis of pyrazole-pyridine conjugates **9F** and **10F** having amide tethers.

To check the synthetic versatility of this oxidative amidation approach, we tested the scope of the methodology with pyrazole-5-carbaldehydes **9** and **10**. Using this method, 3-(4-chlorophenyl)-1-phenyl-*N*-(pyridin-2-yl)-1*H*-pyrazole-5-carboxamide (**10F**) was produced in good yield (62%), while **9F** was generated in low yield (36%) as depicted in Scheme 8.

**Scheme 9:**
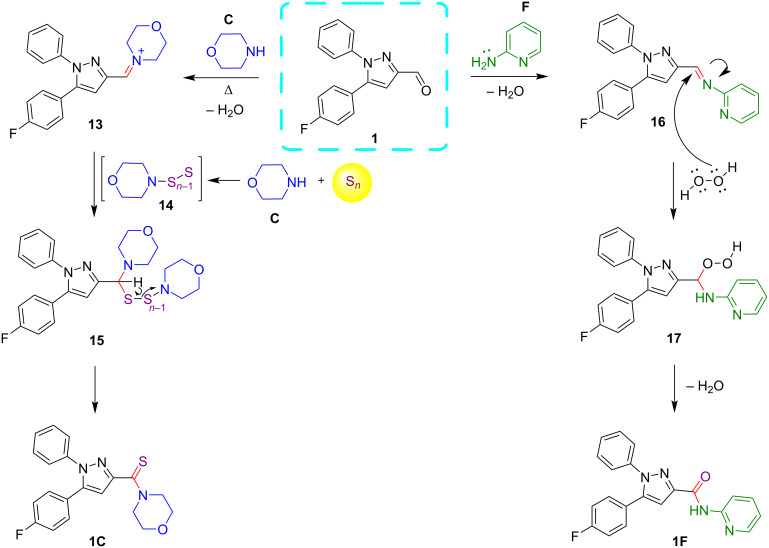
A tentative mechanism for the formation of pyrazole conjugates with thioamide and amide linkage.

Based on the current experimental observations and literature reports [62,83] a plausible mechanistic pathway is outlined in Scheme 9 for the formation of the thioamide and amide-linked pyrazole derivatives **1C** and **1F**. It is proposed that initially pyrazole-3-carbaldehyde **1** reacted with morpholine (**C**) to furnish the iminium intermediate **13**. Meanwhile, the intermediate polysulfide **14** formed by the nucleophilic attack of morpholine (**C**) on elemental sulfur may react with the intermediate **13** to afford another intermediate **15**, which undergoes oxidation to release the thioamide-tethered pyrazole **1C**. On the other hand, the pyrazole carbaldehyde **1** forms imine intermediate **16** by condensation with 2-aminopyridine. Thereafter, a nucleophilic attack of H_2_O_2_ on the imine carbon may afford the intermediate **17**. Finally, the loss of a water molecule from the intermediate **17** may generate the pyrazole-pyridine conjugate with amide linkage **1F**.

## Conclusion

In summary, a simple, straightforward and efficient approach for the construction of biologically interesting highly diversified pyrazole-linked thioamide and amide conjugates has been developed. The pyrazole C-3/4/5-tethered thioamide conjugates were prepared via a one-pot reaction between highly diversified pyrazole carbaldehydes, cyclic secondary amines, and elemental sulfur under metal and catalyst-free conditions. The salient features of the current protocol may be attributed to the broad substrate scope, commercially available secondary amines, operational simplicity, multicomponent character of the reaction, easy isolation of products, short reaction time, and good to excellent yields of the desired molecules. Moreover, a practical synthetic utility of pyrazole-3/5-carbaldehydes has been explored through the formation of amide bond-tethered pyrazole-pyridine conjugates. This developed methodology was successfully carried out by employing commercially available substituted 2-aminopyridines and hydrogen peroxide as an oxidant. The biological evaluation of the thioamide and amide conjugates is underway in our laboratory.

## Experimental

### General information

All chemicals and reagents were purchased from Sigma-Aldrich, Acros, Avera Synthesis, Spectrochem Pvt. Ltd., and used without further purification. Commercially available anhydrous solvents (THF, DMF, benzene, toluene, MeOH, EtOH, and CH_2_Cl_2_ Spectrochem) were used in the reactions. Thin-layer chromatography (TLC) was performed using precoated aluminum plates purchased from E. Merck (silica gel 60 PF254, 0.25 mm). Column chromatography was performed using Spectrochem silica gel (60–120 mesh). Melting points were determined in open capillary tubes on the Precision Digital melting point apparatus (LABCO make) containing silicone oil, and the results are uncorrected. IR spectra (neat) were recorded on an Agilent FTIR spectrophotometer. ^1^H and ^13^C NMR spectra were recorded either on an Avance III Bruker or a JEOL JNM-ECS spectrometer at operating frequencies of 200/400/500 MHz (^1^H) and or 100/125/150 MHz (^13^C) as indicated in the individual spectra using TMS as an internal standard. Elemental analyses were performed on a Carlo-Erba 108 or an Elementar Vario EL III microanalyzer. The room temperature varied between 25 °C and 30 °C. The multiplicities in the ^1^HNMR spectra are presented as s for singlet, d for doublet, dd for doublet of the doublet, td for a triplet of doublet, t for triplet and m for multiplet. The multiplicity in the ^13^C NMR spectra is presented as d for doublet.

### Experimental procedures

**General procedure for the synthesis of compounds 1A–E, 2–4C, 5A–E, 6–8C, 9C, 10A, 11A,B, 11E, and 12C as exemplified for (5-(4-fluorophenyl)-1-phenyl-1*H*-pyrazol-3-yl)(morpholino)methanethione (1C):** In a dry round-bottomed flask, pyrazole-3-carbaldehyde **1** (0.20 g, 0.75 mmol), morpholine (**C**, 0.072 g, 0.83 mmol), and sulfur powder (0.096 g, 3 mmol) were added to dry DMF (2 mL) at room temperature. The reaction flask was heated at 70 °C in an oil bath for 1 h. After completion of the reaction, as determined by TLC, cold water was added to the reaction mixture at room temperature which resulted in precipitation of the product. The product was collected by filtration under reduced pressure using a Büchner funnel and further purified by silica gel column chromatography (60–120 mesh silica gel) using hexane and ethyl acetate as an eluent (80:20, v/v) to give the final product **1C** (0.247 g, 90%; *R*_f_ 0.19 (hexane/EtOAc 90:10, v/v)).

**Gram-scale synthesis of (5-(4-fluorophenyl)-1-phenyl-1*H*-pyrazol-3-yl)(morpholino)methanethione (1C):** A 50 mL round-bottomed flask was charged with pyrazole-3-carbaldehyde **1** (1 g, 3.74 mmol), morpholine (**C**, 0.36 g, 4.14 mmol), and elemental sulfur (0.48 g, 15 mmol) in dry DMF (10 mL) followed by heating of the reaction mixture at 70 °C for 2.5 h. On completion of the reaction, as determined by TLC, the reaction content was cooled to room temperature and poured into ice-cold water under stirring, which resulted in the formation of a precipitate. The solid was collected under vacuum using a Büchner funnel and further purified by silica gel column chromatography (60–120 mesh silica gel) using hexane and ethyl acetate (80:20, v/v) as an eluent to give the analytically pure product **1C** (1.18 g from 1 g, 86%; *R*_f_ 0.19 (hexane/EtOAc 90:10, v/v)).

**Procedure for the synthesis of (5-(4-fluorophenyl)-1-phenyl-1*H*-pyrazol-3-yl)(pyrrolidin-1-yl)methanethione (1A) through control experiment:** In a dry round-bottomed flask, pyrazole-3-carbaldehyde **1** (0.05 g, 0.19 mmol), pyrrolidine (**A**, 0.015 g, 0.21 mmol), and sulfur powder (0.024 g, 0.75 mmol) were added to dry DMF (2 mL) at room temperature. The reaction flask was heated at 70 °C in an oil bath for 3.5 h. After completion of the reaction, as monitored by the TLC, cold water was added to the reaction mixture at room temperature which resulted in the formation of a precipitate. The product was collected by filtration under reduced pressure using a Büchner funnel and further purified by silica gel column chromatography (60–120 mesh silica gel) using hexane and ethyl acetate as an eluent (80:20, v/v) to give final product **1A** (0.049 g, 76%; *R*_f_ 0.68, (hexane/EtOAc 70:30, v/v)).

**Typical procedure for the synthesis of compounds 1F, 1G, 4F, 9F, and 10F as exemplified for 5-(4-fluorophenyl)-1-phenyl-*N*-(pyridin-2-yl)-1*H*-pyrazole-3-carboxamide (1F):** To a stirred solution of compound **1** (0.10 g, 0.37 mmol) and 2-aminopyridine (**F**, 0.04 g, 0.42 mmol) in dry THF, H_2_O_2_ (0.087 mL, 3.73 mmol) was added dropwise at room temperature and the reaction mixture was heated at 70 °C for 20 h. Upon completion of the reaction, as monitored by TLC, the reaction mixture was cooled to room temperature, water was added, and the product was extracted with ethyl acetate (3 × 25 mL). The combined organic layers were washed with brine, dried over anhydrous Na_2_SO_4_, and concentrated under reduced pressure to afford crude product **1F**. This material was purified by silica gel column chromatography (60–120 mesh) using hexane and ethyl acetate as an eluent (95:05, v/v) to get the analytically pure product **1F** (0.082 g, 61%; *R*_f_ 0.63, (hexane/EtOAc 90:10, v/v)).

## Supporting Information

File 1 Analytical data and copies of spectra.
