# Supplementary material for: An efficient metal-free and catalyst-free C–S/C–O bond-formation strategy: synthesis of pyrazole-conjugated thioamides and amides
Source: Beilstein J Org Chem. 2023 Mar 2;19:231–44. doi: 10.3762/bjoc.19.22 (PMC9989676; doi:10.3762/bjoc.19.22)
Supplement: File 1 — Analytical data and copies of spectra. [file Beilstein_J_Org_Chem-19-231-s001.pdf]

BEILSTEIN JOURNAL OF ORGANIC CHEMISTRY

Supporting Information

for

An efficient metal-free and catalyst-free C–S/C–O bond-formation strategy: synthesis of pyrazole-conjugated thioamides and amides

Shubham Sharma, Dharmender Singh, Sunit Kumar, Vaishali, Rahul Jamra, Naveen Banyal, Deepika, Chandi C. Malakar and Virender Singh

Beilstein J. Org. Chem. **2023,** *19,* 231–244. doi:10.3762/bjoc.19.22

Analytical data and copies of spectra

License and Terms: This is a supporting information file under the terms of the Creative Commons Attribution License (https://creativecommons.org/licenses/by/4.0). Please note that the reuse, redistribution and reproduction in particular requires that the author(s) and source are credited and that individual graphics may be subject to special legal provisions.
The license is subject to the *Beilstein Journal of Organic Chemistry* terms and conditions: (https://www.beilstein-journals.org/bjoc/terms)

Table of contents

1. Experimental data S2–S9

2. ^{1}H and ^{13}C NMR spectra of the new products S10–S37

(5-(4-Fluorophenyl)-1-phenyl-1*H*-pyrazol-3-yl)(pyrrolidin-1-yl)methanethione (1A). Yield: 82% (0.215 g from 0.20 g) as a light pink solid; mp 134–136 °C; R_f 0.68 (hexane/EtOAc, 70:30, v/v); IR (neat): ν_{max} (cm^{-1}) = 1232 (C=S) ; ^{1}H NMR (500 MHz, $CDCl_3$): δ = 2.05–2.07 (m, 4 H, $(CH_2)_2$), 4.03–4.06 (m, 2 H, CH_2), 4.14–4.17 (m, 2 H, CH_2), 6.97–7.02 (m, 2 H, ArH), 7.11 (s, 1 H, ArH), 7.19–7.23 (m, 2 H, ArH), 7.24–7.25 (m, 1 H, ArH), 7.27–7.28 (m, 1 H, ArH), 7.33–7.38 (m, 3 H, ArH) ppm; ^{13}C NMR (100 MHz, $CDCl_3$): δ = 24.3, 26.9, 54.6, 54.9, 112.2, 115.8 (d, *J* = 22 Hz), 125.3, 126.0 (d, *J* = 4 Hz), 128.1, 129.2, 130.8 (d, *J* = 8 Hz), 139.6, 142.3, 153.4, 162.8 (d, *J* = 248 Hz), 185.9 ppm; HRMS (ESI) *m/z*: calcd. for $C_{20}H_{18}FN_3S$ [M + H^+]: 352.1283, found: 352.1275.

(5-(4-Fluorophenyl)-1-phenyl-1*H*-pyrazol-3-yl)(piperidin-1-yl)methanethione (1B). Yield: 88% (0.241 g from 0.20 g) as a yellow solid; mp 148–150 °C; R_f 0.60 (hexane/EtOAc, 90:10, v/v); IR (neat): ν_{max} (cm^{-1}) = 1238 (C=S) ; ^{1}H NMR (500 MHz, $CDCl_3$): δ = 1.74–1.84 (m, 6 H, $(CH_2)_3$), 4.02 (t, *J* = 5.2 Hz, 2 H, CH_2), 4.38 (t, *J* = 5.5 Hz, 2 H, CH_2), 6.85 (s, 1 H, ArH), 7.00 (t, *J* = 8.6 Hz, 2 H, ArH), 7.20–7.23 (m, 2 H, ArH), 7.24–7.25 (m, 1 H, ArH), 7.27–7.28 (m, 1 H, ArH), 7.32–7.36 (m, 3 H, ArH) ppm; ^{13}C NMR (100 MHz, $CDCl_3$): δ = 24.5, 25.7, 27.2, 51.6, 54.0, 110.8, 115.8 (d, *J* = 21 Hz), 125.3, 126.0 (d, *J* = 3 Hz), 128.0, 129.2, 130.7 (d, *J* = 8 Hz), 139.5, 142.6, 152.9, 162.8 (d, *J* = 248 Hz), 189.5 ppm; HRMS (ESI) *m/z*: calcd. for $C_{21}H_{20}FN_3S$ [M + H^+]: 366.144, found: 366.1428.

(5-(4-Fluorophenyl)-1-phenyl-1*H*-pyrazol-3-yl)(morpholino)methanethione (1C). Yield: 90% (0.247 g from 0.20 g) as a yellow solid; mp 174–176 °C; R_f 0.19 (hexane/EtOAc, 90:10, v/v); IR (neat): ν_{max} (cm^{-1}) = 1236 (C=S) ; ^{1}H NMR (500 MHz, $CDCl_3$): δ = 3.80 (t, *J* = 4.8 Hz, 2 H, CH_2), 3.91 (t, *J* = 4.9 Hz, 2 H, CH_2), 4.20 (t, *J* = 4.8 Hz, 2 H, CH_2), 4.48 (t, *J* = 4.8 Hz, 2 H, CH_2), 6.94 (s, 1 H, ArH), 7.00 (t, *J* = 8.6 Hz, 2 H, ArH), 7.19–7.22 (m, 2 H, ArH), 7.24–7.26 (m, 2 H, ArH), 7.34–7.37 (m, 3 H, ArH) ppm; ^{13}C NMR (100 MHz, $CDCl_3$): δ = 50.6, 53.3, 66.7, 67.2, 112.0, 115.9 (d, *J* = 22 Hz), 125.3, 125.8 (d, *J* = 3 Hz), 128.2, 129.2, 130.7 (d, *J* = 8 Hz), 139.4, 142.9, 152.2, 162.9 (d, *J* = 248 Hz), 190.0 ppm; HRMS (ESI) *m/z*: calcd. for $C_{20}H_{18}FN_3OS$ [M + H^+]: 368.1233, found: 368.1211.

(5-(4-Fluorophenyl)-1-phenyl-1*H*-pyrazol-3-yl)(thiomorpholino)methanethione (1D). Yield: 89% (0.255 g from 0.20 g) as a yellow solid; mp 190–192 °C; R_f 0.73 (hexane/EtOAc, 90:10, v/v); IR (neat): ν_{max} (cm^{-1}) = 1225 (C=S) ; ^{1}H NMR (500 MHz, $CDCl_3$): δ = 2.85 (t, *J* = 5.0 Hz, 2 H,

CH_2), 2.92 (t, *J* = 5.1 Hz, 2 H, CH_2), 4.39 (t, *J* = 5.0 Hz, 2 H, CH_2), 4.72 (t, *J* = 5.0 Hz, 2 H, CH_2), 6.90 (s, 1 H, ArH), 7.01 (t, *J* = 8.7 Hz, 2 H, ArH), 7.19–7.23 (m, 2 H, ArH), 7.23–7.25 (m, 2 H, ArH), 7.34–7.37 (m, 3 H, ArH) ppm; ^{13}C NMR (100 MHz, $CDCl_3$): δ = 27.5, 29.0, 53.2, 55.5, 111.6, 115.9 (d, *J* = 22 Hz), 125.2, 125.8 (d, *J* = 3 Hz), 128.1, 129.2, 130.8 (d, *J* = 8 Hz), 139.4, 142.8, 152.6, 162.9 (d, *J* = 248 Hz), 190.8 ppm; HRMS (ESI) *m/z*: calcd. for $C_{20}H_{18}FN_3S_2$ [M + H^+]: 384.1004, found: 384.1023.

(5-(4-Fluorophenyl)-1-phenyl-1*H*-pyrazol-3-yl)(4-methylpiperazin-1-yl)methanethione (1E). Yield: 53% (0.152 g from 0.20 g) as a red solid; mp 118–120 °C; R_f 0.24 (hexane/EtOAc, 50:50, v/v); IR (neat): ν_{max} (cm^{-1}) = 1230 (C=S) ; ^{1}H NMR (500 MHz, $CDCl_3$): δ = 2.36 (s, 3 H, NCH_3), 2.55 (t, *J* = 5.0 Hz, 2 H, CH_2), 2.65 (t, *J* = 5.1 Hz, 2 H, CH_2), 4.17 (t, *J* = 4.9 Hz, 2 H, CH_2), 4.48 (t, *J* = 4.5 Hz, 2 H, CH_2), 6.89 (s, 1 H, ArH), 7.00 (t, *J* = 8.6 Hz, 2 H, ArH), 7.19–7.23 (m, 2 H, ArH), 7.24–7.25 (m, 1 H, ArH), 7.27 (s, 1 H, ArH), 7.33–7.38 (m, 3 H, ArH) ppm; ^{13}C NMR (125 MHz, $CDCl_3$): δ = 45.7, 50.0, 52.5, 54.5, 55.5, 111.5, 115.8 (d, *J* = 22.5 Hz), 125.3, 125.9 (d, *J* = 3.8 Hz), 128.1, 129.2, 130.8 (d, *J* = 7.5 Hz), 139.5, 142.8, 152.6, 162.9 (d, *J* = 247.5 Hz), 190.3 ppm; HRMS (ESI) *m/z*: calcd. for $C_{21}H_{21}FN_4S$ [M + H^+]: 381.1549, found: 381.1522.

(5-(4-Chlorophenyl)-1-methyl-1*H*-pyrazol-3-yl)(morpholino)methanethione (2C). Yield: 88% (0.255 g from 0.20 g) as a pale yellow solid; mp 144–146 °C; R_f 0.12 (hexane/EtOAc, 90:10, v/v); IR (neat): ν_{max} (cm^{-1}) = 1225 (C=S) ; ^{1}H NMR (500 MHz, $CDCl_3$): δ = 3.77 (t, *J* = 4.8 Hz, 2 H, CH_2), 3.85 (s, 3 H, NCH_3), 3.89 (t, *J* = 4.8 Hz, 2 H, CH_2), 4.12 (t, *J* = 4.8 Hz, 2 H, CH_2), 4.45 (t, *J* = 4.9 Hz, 2 H, CH_2), 6.70 (s, 1 H, ArH), 7.35–7.38 (m, 2 H, ArH), 7.44–7.46 (m, 2 H, ArH) ppm; ^{13}C NMR (125 MHz, $CDCl_3$): δ = 40.0, 50.4, 53.1, 66.6, 67.1, 109.9, 128.1, 129.2, 130.1, 135.2, 143.3, 150.8, 190.9 ppm; HRMS (ESI) *m/z*: calcd. for $C_{15}H_{16}ClN_3OS$ [M + H^+]: 322.0781, found: 322.0792.

(5-(4-Chlorophenyl)-1-phenyl-1*H*-pyrazol-3-yl)(morpholino)methanethione (3C). Yield: 87% (0.235 g from 0.20 g) as a white solid; mp 158–160 °C; R_f 0.52 (hexane/EtOAc, 90:10, v/v); IR (neat): ν_{max} (cm^{-1}) = 1245 (C=S) ; ^{1}H NMR (500 MHz, $CDCl_3$): δ = 3.80 (t, *J* = 4.8 Hz, 2 H, CH_2), 3.90 (t, *J* = 4.8 Hz, 2 H, CH_2), 4.22 (t, *J* = 4.8 Hz, 2 H, CH_2), 4.48 (t, *J* = 4.8 Hz, 2 H, CH_2), 6.96 (s, 1 H, ArH), 7.15–7.17 (m, 2 H, ArH), 7.24–7.25 (m, 1 H, ArH), 7.27–7.29 (m, 3 H, ArH), 7.36–7.37 (m, 3 H, ArH) ppm; ^{13}C NMR (125 MHz, $CDCl_3$): δ = 50.5, 53.2, 66.6, 67.1, 112.0,

125.3, 128.1, 128.3, 128.9, 129.2, 130.0, 134.8, 139.3, 142.6, 152.3, 190.3 ppm; HRMS (ESI) *m/z*: calcd. for $C_{20}H_{18}ClN_3OS$ [M + H$^+$]: 384.0937, found: 384.0955.

(1,5-Diphenyl-1*H*-pyrazol-3-yl)(morpholino)methanethione (4C). Yield: 86% (0.121 g from 0.10 g) as a yellow solid; mp 160–162 °C; R_f 0.21 (hexane/EtOAc, 90:10, v/v); IR (neat): ν_{max} (cm^{-1}) = 1237 (C=S) ; ^{1}H NMR (500 MHz, $CDCl_3$): δ = 3.80 (t, *J* = 4.8 Hz, 2 H, CH_2), 3.91 (t, *J* = 4.8 Hz, 2 H, CH_2), 4.23 (t, *J* = 4.8 Hz, 2 H, CH_2), 4.49 (t, *J* = 4.8 Hz, 2 H, CH_2), 6.96 (s, 1 H, ArH), 7.22–7.24 (m, 2 H, ArH), 7.27–7.28 (m, 1 H, ArH), 7.30–7.32 (m, 3 H, ArH), 7.33–7.35 (m, 4 H, ArH) ppm; ^{13}C NMR (125 MHz, $CDCl_3$): δ = 50.5, 53.2, 66.6, 67.2, 111.8, 125.3, 128.1, 128.6, 128.7, 128.8, 129.1, 129.6, 139.6, 143.9, 152.2, 190.6 ppm; HRMS (ESI) *m/z*: calcd. for $C_{20}H_{19}N_3OS$ [M + H$^+$]: 350.1327, found: 350.1338.

Ethyl 1-phenyl-4-(pyrrolidine-1-carbonothioyl)-1*H*-pyrazole-3-carboxylate (5A). Yield: 70% (0.186 g from 0.20 g) as a light brown solid; mp 175–177 °C; R_f 0.47 (hexane/EtOAc, 90:10, v/v); IR (neat): ν_{max} (cm^{-1}) = 1695 (CO_2Et), 1232 (C=S); ^{1}H NMR (500 MHz, $CDCl_3$): δ = 1.39 (t, *J* = 7.1 Hz, 3 H, $CO_2CH_2\mathit{CH_3}$), 2.01 (t, *J* = 6.8 Hz, 2 H, CH_2), 2.11 (t, *J* = 7.0 Hz, 2 H, CH_2), 3.50 (t, *J* = 6.8 Hz, 2 H, CH_2), 3.99 (t, *J* = 7.1 Hz, 2 H, CH_2), 4.41 (q, *J* = 7.1 Hz, 2 H, $CO_2\mathit{CH_2}CH_3$), 7.38 (t, *J* = 7.4 Hz, 1 H, ArH), 7.48 (t, *J* = 7.9 Hz, 2 H, ArH), 7.72 (d, *J* = 7.8 Hz, 2 H, ArH), 8.04 (s, 1 H, ArH) ppm; ^{13}C NMR (125 MHz, $CDCl_3$): δ = 14.4, 24.8, 26.3, 53.1, 53.3, 61.5, 120.1, 127.4, 128.2, 129.6, 129.7, 138.8, 139.1, 161.5, 186.5 ppm; HRMS (ESI) *m/z*: calcd. for $C_{17}H_{19}N_3O_2S$ [M + H$^+$]: 330.1276, found: 330.1292.

Ethyl 1-phenyl-4-(piperidine-1-carbonothioyl)-1*H*-pyrazole-3-carboxylate (5B). Yield: 49% (0.135 g from 0.20 g) as a off white solid; mp 155–157 °C; R_f 0.61 (hexane/EtOAc, 90:10, v/v); IR (neat): ν_{max} (cm^{-1}) = 1698 (CO_2Et), 1241 (C=S) ; ^{1}H NMR (500 MHz, $CDCl_3$): δ = 1.39 (t, *J* = 7.1 Hz, 3 H, $CO_2CH_2\mathit{CH_3}$), 1.58 (s, 2 H, CH_2), 1.71–1.86 (m, 5 H, $(CH_2)_2$ and CH), 3.59 (t, *J* = 5.5 Hz, 2 H, CH_2), 4.41 (q, *J* = 7.1 Hz, 3 H, $CO_2\mathit{CH_2}CH_3$ and CH), 7.37 (t, *J* = 7.4 Hz, 1 H, ArH), 7.48 (t, *J* = 7.9 Hz, 2 H, ArH), 7.73 (d, *J* = 7.8 Hz, 2 H, ArH), 8.02 (s, 1 H, ArH) ppm; ^{13}C NMR (125 MHz, $CDCl_3$): δ = 14.5, 24.1, 25.3, 26.3, 50.2, 53.1, 61.5, 120.1, 127.5, 128.1, 128.7, 129.6, 138.8, 139.1, 161.3, 188.2 ppm; HRMS (ESI) *m/z*: calcd. for $C_{18}H_{21}N_3O_2S$ [M + H$^+$]: 344.1432, found: 344.145.

Ethyl 4-(morpholine-4-carbonothioyl)-1-phenyl-1*H*-pyrazole-3-carboxylate (5C). Yield: 76% (0.211 g from 0.20 g) as a white solid; mp 170–172 °C; R_f 0.25 (hexane/EtOAc, 90:10, v/v); IR (neat): ν_{max} (cm^{-1}) = 1230 (C=S), 1707 (CO_2Et) ; ^1H NMR (500 MHz, $CDCl_3$): δ = 1.41 (t, *J* = 7.1 Hz, 3 H, $CO_2CH_2\mathit{CH_3}$), 3.62–3.68 (m, 4 H, $(CH_2)_2$), 3.91 (t, *J* = 4.9 Hz, 2 H), 4.40–4.49 (m, 4 H, $CO_2\mathit{CH_2}CH_3$ and CH_2), 7.39 (t, *J* = 7.4 Hz, 1 H, ArH), 7.49 (t, *J* = 7.9 Hz, 2 H, ArH), 7.71–7.73 (m, 2 H, ArH), 8.05 (s, 1 H, ArH) ppm; ^{13}C NMR (125 MHz, $CDCl_3$): δ = 14.5, 49.2, 52.2, 61.7, 66.2, 66.3, 120.2, 128.0, 128.2, 128.3, 129.7, 138.8, 139.0, 161.3, 189.7 ppm; HRMS (ESI) *m/z*: calcd. for $C_{17}H_{19}N_3O_3S$ [M + H^+]: 346.1225, found: 346.1216.

Ethyl 1-phenyl-4-(thiomorpholine-4-carbonothioyl)-1*H*-pyrazole-3-carboxylate (5D). Yield: 34% (0.101 g from 0.20 g) as a light brown solid; mp 170–172 °C; R_f 0.35 (hexane/EtOAc, 80:20, v/v); IR (neat): ν_{max} (cm^{-1}) = 1711 (CO_2Et), 1234 (C=S) ; ^1H NMR (500 MHz, $CDCl_3$): δ = 1.41 (t, *J* = 7.1 Hz, 3 H, $CO_2CH_2\mathit{CH_3}$), 2.64 (t, *J* = 5.0 Hz, 2 H, CH_2), 2.92 (t, *J* = 5.1 Hz, 2 H, CH_2), 3.93 (t, *J* = 5.1 Hz, 2 H, CH_2), 4.42 (q, *J* = 7.1 Hz, 2 H, $CO_2\mathit{CH_2}CH_3$), 4.73 (s, 2 H, CH_2), 7.39 (t, *J* = 7.4 Hz, 1 H, ArH), 7.49 (t, *J* = 7.9 Hz, 2 H, ArH), 7.71–7.74 (m, 2 H, ArH), 8.02 (s, 1 H, ArH) ppm; ^{13}C NMR (125 MHz, $CDCl_3$): δ = 14.5, 27.2, 27.9, 51.6, 54.6, 61.7, 120.1, 127.6, 128.2, 128.3, 129.7, 138.7, 139.0, 161.2, 190.0 ppm; HRMS (ESI) *m/z*: calcd. for $C_{17}H_{19}N_3O_2S_2$ [M + H^+]: 362.0997, found: 362.0981.

Ethyl 4-(4-methylpiperazine-1-carbonothioyl)-1-phenyl-1*H*-pyrazole-3-carboxylate (5E). Yield: 56% (0.164 g from 0.20 g) as a yellow solid; mp 124–126 °C; R_f 0.13 (hexane/EtOAc, 80:20, v/v); IR (neat): ν_{max} (cm^{-1}) = 1705 (CO_2Et), 1233 (C=S); ^1H NMR (500 MHz, $CDCl_3$): δ = 1.40 (t, *J* = 7.1 Hz, 3 H, $CO_2CH_2\mathit{CH_3}$), 2.35 (s, 3 H, NCH_3), 2.41 (s, 2 H, CH_2), 2.64 (s, 2 H, CH_2), 3.65 (t, *J* = 5.1 Hz, 2 H, CH_2), 4.40–4.50 (m, 4 H, $CO_2\mathit{CH_2}CH_3$ and CH_2), 7.36– 7.40 (m, 1 H, ArH), 7.46–7.50 (m, 2 H, ArH), 7.72–7.74 (m, 2 H, ArH), 8.04 (s, 1 H, ArH) ppm; ^{13}C NMR (125 MHz, $CDCl_3$): δ = 14.5, 45.7, 48.7, 51.7, 54.1, 54.5, 61.6, 120.1, 127.9, 128.2, 129.7, 138.9, 139.0, 161.2, 189.4 ppm; HRMS (ESI) *m/z*: calcd. for $C_{18}H_{22}N_4O_2S$ [M + H^+]: 359.1541, found: 359.152.

Ethyl 4-(morpholine-4-carbonothioyl)-1-(*p*-tolyl)-1*H*-pyrazole-3-carboxylate (6C). Yield: 66% (0.184 g from 0.20 g) as a off white solid; mp 158–160 °C; R_f 0.44 (hexane/EtOAc, 70:30, v/v); IR (neat): ν_{max} (cm^{-1}) = 1710 (CO_2Et), 1234 (C=S); ^1H NMR (500 MHz, $CDCl_3$): δ = 1.40 (t, *J* = 7.1 Hz, 3 H, $CO_2CH_2\mathit{CH_3}$), 2.40 (s, 3 H, $ArCH_3$), 3.62–3.68 (m, 4 H, $(CH_2)_2$), 3.91 (t, *J* = 4.9 Hz, 2 H, CH_2), 4.40–4.47 (m, 4 H, $CO_2\mathit{CH_2}CH_3$ and CH_2), 7.27–7.28 (m, 2 H, ArH), 7.58–7.60

(m, 2 H, ArH), 8.01–8.02 (m, 1 H, ArH) ppm; ^{13}C NMR (125 MHz, $CDCl_3$): δ = 14.5, 21.1, 49.2, 52.2, 61.6, 66.2, 66.3, 120.0, 127.8, 128.1, 130.2, 136.7, 138.4, 138.5, 161.3, 189.8 ppm; HRMS (ESI) *m/z*: calcd. for $C_{18}H_{21}N_3O_3S$ [M + H^+]: 360.1382, found: 360.1362.

Ethyl 1-(3,4-dimethylphenyl)-4-(morpholine-4-carbonothioyl)-1*H*-pyrazole-3-carboxylate (7C). Yield: 60% (0.164 g from 0.20 g) as a light yellow solid; mp 160–162 °C; R_f 0.35 (hexane/EtOAc, 70:30, v/v); IR (neat): ν_{max} (cm^{-1}) = 1717 (CO_2Et), 1220 (C=S); ^{1}H NMR (500 MHz, $CDCl_3$): δ = 1.40 (t, J = 7.1 Hz, 3 H, $CO_2CH_2\mathit{CH_3}$), 2.29 (s, 3 H, $ArCH_3$), 2.32 (s, 3 H, $ArCH_3$), 3.62–3.68 (m, 4 H, $(CH_2)_2$), 3.90 (t, J = 4.9 Hz, 2 H, CH_2), 4.39–4.49 (m, 4 H, $CO_2\mathit{CH_2}CH_3$ and CH_2), 7.21 (d, J = 8.2 Hz, 1 H, ArH), 7.39 (dd, J_1 = 8.1, J_2 = 2.4 Hz, 1 H, ArH), 7.52 (d, J = 2.2 Hz, 1 H, ArH), 8.01 (s, 1 H, ArH) ppm; ^{13}C NMR (125 MHz, $CDCl_3$): δ = 14.5, 19.5, 20.0, 49.2, 52.2, 61.6, 66.2, 66.3, 117.3, 121.3, 127.7, 128.2, 130.6, 136.9, 137.1, 138.3, 138.4, 161.4, 189.9 ppm; HRMS (ESI) *m/z*: calcd. for $C_{19}H_{23}N_3O_3S$ [M + H^+]: 374.1538, found: 374.1554.

Ethyl 1-(4-chlorophenyl)-4-(morpholine-4-carbonothioyl)-1*H*-pyrazole-3-carboxylate (8C). Yield: 68% (0.184 g from 0.20 g) as a light pink solid; mp 188–190 °C; R_f 0.28 (hexane/EtOAc, 70:30, v/v); IR (neat): ν_{max} (cm^{-1}) = 1696 (CO_2Et), 1236 (C=S) ; ^{1}H NMR (500 MHz, $CDCl_3$): δ = 1.41 (t, J = 7.1 Hz, 3 H, $CO_2CH_2\mathit{CH_3}$), 3.61–3.68 (m, 4 H, $(CH_2)_2$), 3.91 (t, J = 4.9 Hz, 2 H, CH_2), 4.40–4.46 (m, 4 H, $CO_2\mathit{CH_2}CH_3$ and CH_2), 7.45–7.47 (m, 2 H, ArH), 7.67–7.69 (m, 2 H, ArH), 8.02 (s, 1 H, ArH) ppm; ^{13}C NMR (100 MHz, $CDCl_3$): δ = 14.5, 49.1, 52.2, 61.8, 66.1, 66.3, 121.2, 128.0, 128.2, 129.8, 134.0, 137.5, 139.0, 161.1, 189.3 ppm; HRMS (ESI) *m/z*: calcd. for $C_{17}H_{18}ClN_3O_3S$ [M + H^+]: 380.0835, found: 380.0851.

(3-(4-Chlorophenyl)-1-methyl-1*H*-pyrazol-5-yl)(morpholino)methanethione (9C). Yield: 67% (0.097 g from 0.10 g) as a light yellow solid; mp 196–198 °C; R_f 0.20 (hexane/EtOAc, 70:30, v/v); IR (neat): ν_{max} (cm^{-1}) = 1226 (C=S) ; ^{1}H NMR (500 MHz, $CDCl_3$): δ = 3.69 (s, 4 H, $(CH_2)_2$), 3.88–3.91 (m, 5 H, NCH_3 and CH_2), 4.41–4.43 (m, 2 H, CH_2), 6.43 (s, 1 H, ArH), 7.34–7.37 (m, 2 H, ArH), 7.67–7.69 (m, 2 H, ArH) ppm; ^{13}C NMR (125 MHz, $CDCl_3$): δ = 37.6, 48.9, 52.5, 66.5, 66.8, 101.4, 126.9, 128.9, 131.2, 133.8, 142.4, 149.5, 187.9 ppm; HRMS (ESI) *m/z*: calcd. for $C_{15}H_{16}ClN_3OS$ [M + H^+]: 322.0781, found: 322.0789.

(3-(4-Chlorophenyl)-1-phenyl-1*H*-pyrazol-5-yl)(pyrrolidin-1-yl)methanethione (10A). Yield: 71% (0.092 g from 0.10 g) as a brown solid; mp 168–170 °C; R_f 0.43 (hexane/EtOAc, 90:10, v/v);

IR (neat): ν_{max} (cm^{-1}) = 1217 (C=S); ^1H NMR (500 MHz, $CDCl_3$): δ = 2.05–2.12 (m, 4 H, $(CH_2)_2$), 3.75 (t, *J* = 6.6 Hz, 2 H, CH_2), 4.01 (t, *J* = 6.8 Hz, 2 H, CH_2), 7.07 (t, *J* = 8.6 Hz, 2 H, CH_2), 7.17–7.19 (m, 2 H, ArH), 7.26–7.31 (m, 6 H, ArH) ppm; ^{13}C NMR (125 MHz, $CDCl_3$): δ = 24.3, 26.0, 52.9, 53.2, 105.7, 122.6, 127.2, 127.8, 129.0, 129.4, 131.0, 134.1, 139.8, 144.2, 151.1, 185.0 ppm; HRMS (ESI) *m/z*: calcd. for $C_{20}H_{18}ClN_3S$ [M + H^+]: 368.0988, found: 368.0994.

(5-(4-Fluorophenyl)-4-iodo-1-phenyl-1*H*-pyrazol-3-yl)(pyrrolidin-1-yl)methanethione (11A). Yield: 90% (0.11 g from 0.10 g) as a brown solid; mp 162–164 °C; R_f 0.28 (hexane/EtOAc, 90:10, v/v); IR (neat): ν_{max} (cm^{-1}) = 1227 (C=S); ^1H NMR (500 MHz, $CDCl_3$): δ = 2.05 (t, *J* = 3.3 Hz, 4 H, $(CH_2)_2$), 4.05 (t, *J* = 7.0 Hz, 2 H, CH_2), 4.16 (t, *J* = 6.7 Hz, 2 H, CH_2), 7.00 (t, *J* = 8.7 Hz, 2 H, ArH), 7.12 (s, 1 H, ArH), 7.20–7.28 (m, 2 H, ArH), 7.27–7.28 (m, 1 H, ArH), 7.33–7.37 (m, 3 H, ArH) ppm; ^{13}C NMR (125 MHz, $CDCl_3$): δ = 24.2, 26.8, 54.5, 54.8, 112.2, 115.8 (d, *J* = 22.5 Hz), 125.2, 126.0, (d, *J* = 2.5 Hz), 128.0, 129.1, 130.7 (d, *J* = 7.5 Hz), 139.6, 142.3, 153.3, 162.8 (d, *J* = 248.8 Hz), 185.8 ppm; HRMS (ESI) *m/z*: calcd. for $C_{20}H_{17}FIN_3S$ [M + H^+]: 478.025, found: 478.0268.

(5-(4-Fluorophenyl)-4-iodo-1-phenyl-1*H*-pyrazol-3-yl)(piperidin-1-yl)methanethione (11B). Yield: 92% (0.115 g from 0.10 g) as a light pink solid; mp 190–192 °C; R_f 0.47 (hexane/EtOAc, 90:10, v/v); IR (neat): ν_{max} (cm^{-1}) = 1238 (C=S); ^1H NMR (500 MHz, $CDCl_3$): δ = 1.74-1.78 (m, 4 H, $(CH_2)_2$), 1.83–1.88 (m, 2 H, CH_2), 3.66–3.68 (m, 2 H, CH_2), 4.38 (t, *J* = 5.6 Hz, 2 H, CH_2), 7.07 (t, *J* = 8.7 Hz, 2 H, ArH), 7.18–7.20 (m, 2 H, ArH), 7.27–7.30 (m, 5 H, ArH) ppm; ^{13}C NMR (125 MHz, $CDCl_3$): δ = 24.3, 25.5, 27.2, 50.0, 53.5, 63.1, 115.9 (d, *J* = 22.5 Hz), 124.8, 125.4, 128.0, 129.0, 132.4 (d, *J* = 8.8 Hz), 139.3, 143.8, 154.8, 163.1 (d, *J* = 248.8 Hz), 189.1 ppm; HRMS (ESI) *m/z*: calcd. for $C_{21}H_{19}FIN_3S$ [M + H^+]: 492.0406, found: 492.0416.

(5-(4-Fluorophenyl)-4-iodo-1-phenyl-1*H*-pyrazol-3-yl)(4-methylpiperazin-1-yl)methanethione (11E). Yield: 62% (0.079 g from 0.10 g) as a light brown solid; mp 196–198 °C; R_f 0.32 (hexane/EtOAc, 90:10, v/v); IR (neat): ν_{max} (cm^{-1}) = 1226 (C=S); ^1H NMR (500 MHz, $CDCl_3$): δ = 2.37 (s, 3 H, NCH_3), 2.56 (t, *J* = 5.1 Hz, 2 H, CH_2), 2.66 (t, *J* = 5.2 Hz, 2 H, CH_2), 3.75 (t, *J* = 5.1 Hz, 2 H, CH_2), 4.47 (t, *J* = 4.8 Hz, 2 H, CH_2), 7.07 (t, *J* = 8.6 Hz, 2 H, ArH), 7.18–7.19 (m, 2 H, ArH), 7.28–7.30 (m, 5 H, ArH) ppm; ^{13}C NMR (125 MHz, $CDCl_3$): δ = 45.8, 48.5, 52.0, 54.3, 55.4, 63.4, 116.0 (d, *J* = 22.5 Hz), 124.8, 125.3, 128.1, 129.1, 132.4 (d, *J* = 8.8 Hz),

139.3, 143.9, 154.4, 163.1 (d, J = 248.8 Hz), 190.0 ppm; HRMS (ESI) m/z: calcd. for $C_{21}H_{20}FIN_4S$ [M + H^+]: 507.0515, found: 507.0502.

(4-Iodo-1,5-diphenyl-1*H*-pyrazol-3-yl)(morpholino)methanethione (12C). Yield: 58% (0.089 g from 0.132 g) as a off white solid; mp 200–202 °C; R_f 0.45 (hexane/EtOAc, 90:10, v/v); IR (neat): ν_{max} (cm^{-1}) = 1238 (C=S); ^1H NMR (500 MHz, $CDCl_3$): δ = 3.76–3.82 (m, 4 H, $(CH_2)_2$), 3.92 (t, J = 4.8 Hz, 2 H, CH_2), 4.47 (t, J = 4.8 Hz, 2 H, CH_2), 7.19–7.20 (m, 2 H, ArH), 7.26–7.30 (m, 5 H, ArH), 7.36–7.40 (m, 3 H, ArH) ppm; ^{13}C NMR (125 MHz, $CDCl_3$): δ = 48.9, 52.6, 63.2, 66.5, 67.1, 124.8, 128.0, 128.7, 129.0, 129.2, 129.5, 130.4, 139.4, 145.0, 154.0, 190.5 ppm; HRMS (ESI) m/z: calcd. for $C_{20}H_{18}IN_3OS$ [M + H^+]: 476.0293, found: 476.0273.

5-(4-Fluorophenyl)-1-phenyl-*N*-(pyridin-2-yl)-1*H*-pyrazole-3-carboxamide (1F). Yield: 61 % (0.082 g from 0.10 g) as a white solid; mp 176–178 °C; R_f 0.63 (hexane/EtOAc, 90:10, v/v); IR (neat): ν_{max} (cm^{-1}) = 3367 (NH); ^1H NMR (400 MHz, $CDCl_3$): δ = 7.01–7.08 (m, 3 H, ArH), 7.11 (s, 1 H, ArH), 7.21–7.24 (m, 2 H, ArH), 7.31–7.33 (m, 2 H, ArH), 7.39–7.41 (m, 3 H, ArH), 7.76 (t, J = 7.3 Hz, 1 H, ArH), 8.33 (d, J = 3.8 Hz, 1 H, ArH), 8.40 (d, J = 8.3 Hz, 1 H, ArH), 9.47 (s, 1 H, NH) ppm; ^{13}C NMR (125 MHz, $CDCl_3$): δ = 108.5, 114.2, 115.9 (d, J = 22.5 Hz), 119.8, 125.3, 125.8 (d, J = 3.8 Hz), 128.5, 129.2, 130.8 (d, J = 8.8 Hz), 138.5, 139.3, 144.4, 146.7, 148.1, 151.3, 161.0 (d, J = 238.8 Hz), 164.0 ppm; HRMS (ESI) m/z: calcd. for $C_{21}H_{15}FN_4O$ [M + H^+]: 359.1308, found: 359.1319.

5-(4-Fluorophenyl)-*N*-(5-nitropyridin-2-yl)-1-phenyl-1*H*-pyrazole-3-carboxamide (1G). Yield: 34 % (0.051 g from 0.10 g) as a light yellow solid; mp 170–172 °C; R_f 0.76 (hexane/EtOAc, 90:10, v/v); IR (neat): ν_{max} (cm^{-1}) = 3250 (NH) ; ^1H NMR (500 MHz, $CDCl_3$): δ = 7.04 (t, J = 8.6 Hz, 2 H, ArH), 7.14 (s, 1 H, ArH), 7.21–7.24 (m, 2 H, ArH), 7.32–7.34 (m, 2 H, ArH), 7.42–7.43 (m, 3 H, ArH), 8.54–8.61 (m, 2 H, ArH), 9.20 (d, J = 2.4 Hz, 1 H, ArH), 9.81 (s, 1 H, NH) ppm; ^{13}C NMR (150 MHz, $CDCl_3$): δ = 108.7, 113.0, 116.1 (d, J = 22.5 Hz), 125.4, 125.5 (d, J = 3 Hz), 128.9, 129.4, 130.8 (d, J = 7.5 Hz), 134.1, 139.2, 140.6, 145.0, 145.1, 145.9, 155.4, 160.2, 163.1 (d, J = 247.5 Hz) ppm; HRMS (ESI) m/z: calcd. for $C_{21}H_{14}FN_5O_3$ [M + H^+]: 404.1159, found: 404.1123.

1,5-Diphenyl-*N*-(pyridin-2-yl)-1*H*-pyrazole-3-carboxamide (4F). Yield: 70 % (0.144 g from 0.15 g) as a off white solid; mp 188–190 °C; R_f 0.50 (hexane/EtOAc, 90:10, v/v); IR (neat): ν_{max}

(cm^{-1}) = 3398 (NH); ^{1}H NMR (500 MHz, $CDCl_3$): δ = 7.05–7.07 (m, 1 H, ArH), 7.13 (s, 1 H, ArH), 7.24 (d, *J* = 1.9 Hz, 1 H, ArH), 7.31–7.36 (m, 6 H, ArH), 7.37–7.39 (m, 3 H, ArH), 7.74–7.77 (m, 1 H, ArH), 8.33 (d, *J* = 3.8 Hz, 1 H, ArH), 8.41 (d, *J* = 8.4 Hz, 1 H, ArH), 9.51 (s, 1 H, NH) ppm; ^{13}C NMR (125 MHz, $CDCl_3$): δ = 108.5, 114.2, 119.8, 125.3, 128.4, 128.8, 128.9, 129.0, 129.1, 129.7, 138.5, 139.5, 145.4, 146.7, 148.1, 151.4, 160.2 ppm; HRMS (ESI) *m/z*: calcd. for $C_{21}H_{16}N_4O$ [M + H$^+$]: 341.1402, found: 341.1424.

3-(4-Chlorophenyl)-1-methyl-*N*-(pyridin-2-yl)-1*H*-pyrazole-5-carboxamide (9F). Yield: 36 % (0.051 g from 0.10 g) as a light yellow solid; mp 213–215 °C; R_f 0.66 (hexane/EtOAc, 90:10, v/v); IR (neat): ν_{max} (cm^{-1}) = 3082 (NH); ^{1}H NMR (500 MHz, $CDCl_3$): δ = 4.28 (s, 3 H, NCH_3), 7.15–7.20 (m, 1 H, ArH), 7.29 (s, 1 H, ArH), 7.39 (d, *J* = 6.9 Hz, 2 H, ArH), 7.75 (d, *J* = 7.6 Hz, 2 H, ArH), 7.85 (t, *J* = 7.2 Hz, 1 H, ArH), 8.27 (s, 1 H, ArH), 8.41 (d, *J* = 7.7 Hz, 1 H, ArH), 9.77 (s, 1 H, NH) ppm; ^{13}C NMR (125 MHz, d^6-DMSO): δ = 39.6, 106.0, 114.7, 120.2, 126.6, 129.0, 131.4, 132.4, 136.4, 138.3, 147.1, 148.1, 151.6, 158.3 ppm; HRMS (ESI) *m/z*: calcd. for $C_{16}H_{13}ClN_4O$ [M + H$^+$]: 313.0856, found: 313.084.

3-(4-Chlorophenyl)-1-phenyl-*N*-(pyridin-2-yl)-1*H*-pyrazole-5-carboxamide (10F). Yield: 62 % (0.082 g from 0.10 g) as a light yellow solid; mp 192–194 °C; R_f 0.22 (hexane/EtOAc, 90:10, v/v); IR (neat): ν_{max} (cm^{-1}) = 3063 (NH); ^{1}H NMR (500 MHz, $CDCl_3$): δ = 7.12–7.15 (m, 1 H, ArH), 7.29–7.31 (m, 1 H, ArH), 7.38 (s, 1 H, ArH), 7.41 (d, *J* = 8.5 Hz, 2 H, ArH), 7.48 (s, 2 H, ArH), 7.50–7.52 (m, 2 H, ArH), 7.75–7.77 (m, 2 H, ArH), 7.81 (d, *J* = 8.4 Hz, 1 H, ArH), 8.21–8.22 (m, 1 H, ArH), 8.30 (d, *J* = 8.5 Hz, 1 H, ArH), 9.91 (s, 1 H, NH) ppm; ^{13}C NMR (125 MHz, $CDCl_3$): δ = 107.2, 110.3, 115.9, 120.4, 125.6, 127.3, 128.6, 129.1, 130.9, 134.3, 137.2, 140.3, 145.8, 150.6, 158.1, 163.8 ppm; HRMS (ESI) *m/z*: calcd. for $C_{21}H_{15}ClN_4O$ [M + H$^+$]: 375.1012, found: 375.1025.

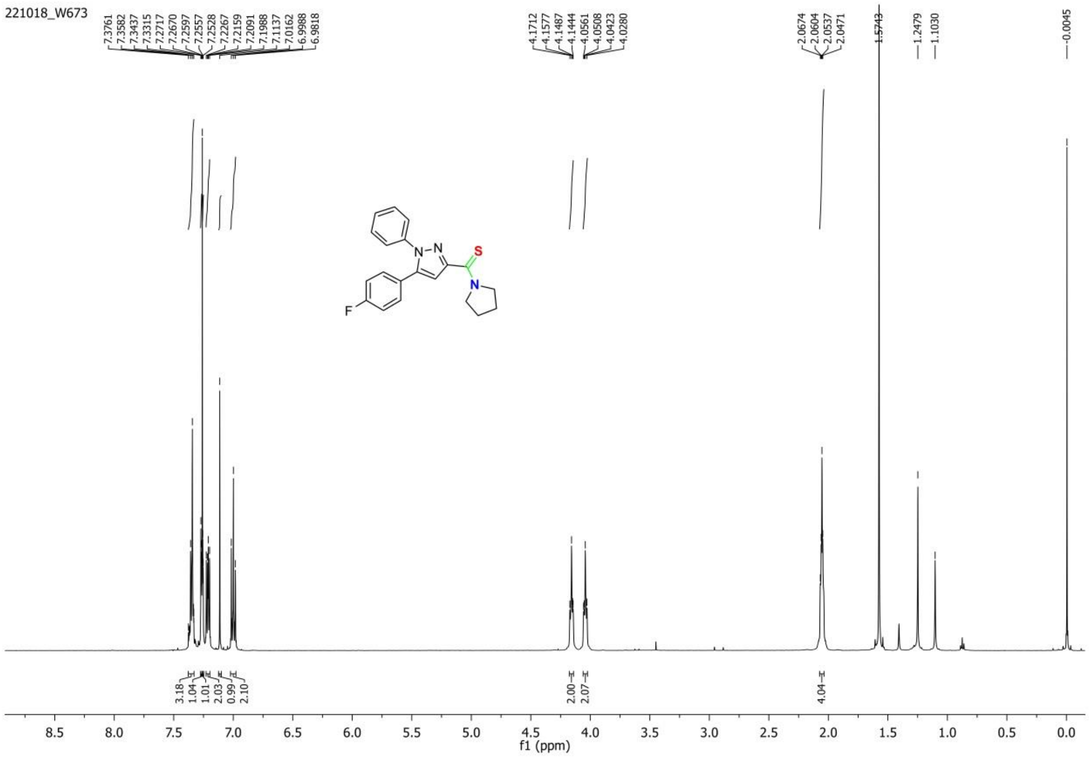

Figure S1: ^{1}H NMR spectrum of **1A**.

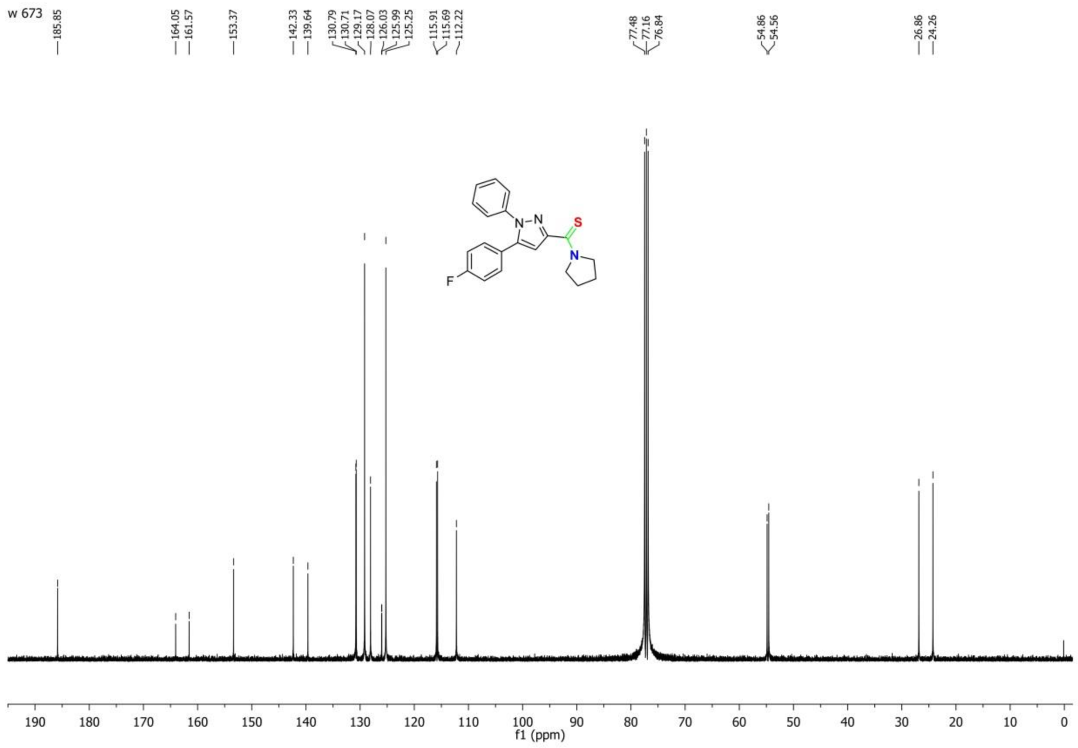

Figure S2: ^{13}C NMR spectrum of **1A**.

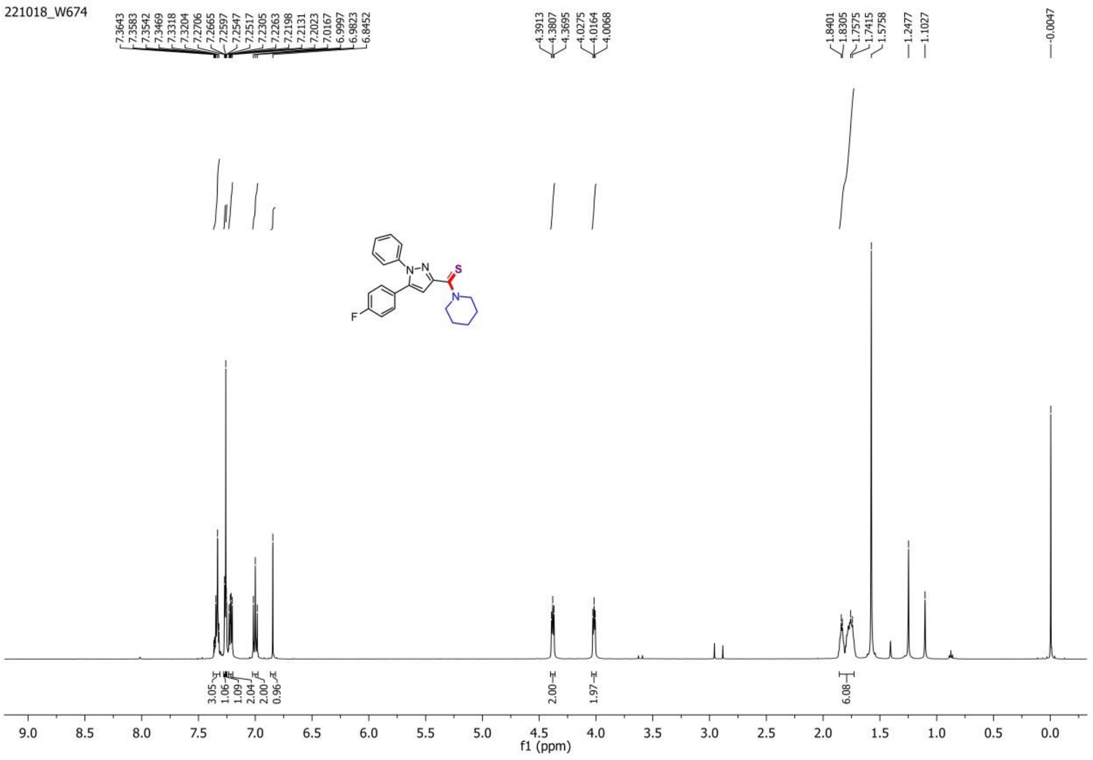

Figure S3: ^{1}H NMR spectrum of **1B**.

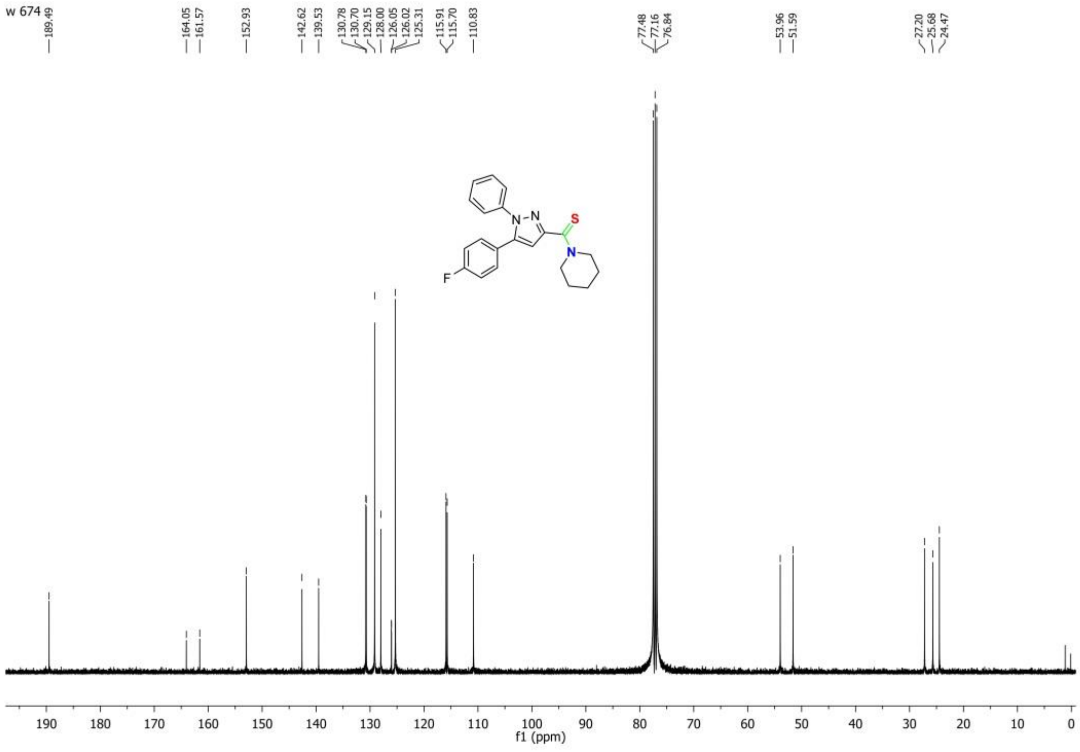

Figure S4: ^{13}C NMR spectrum of **1B**.

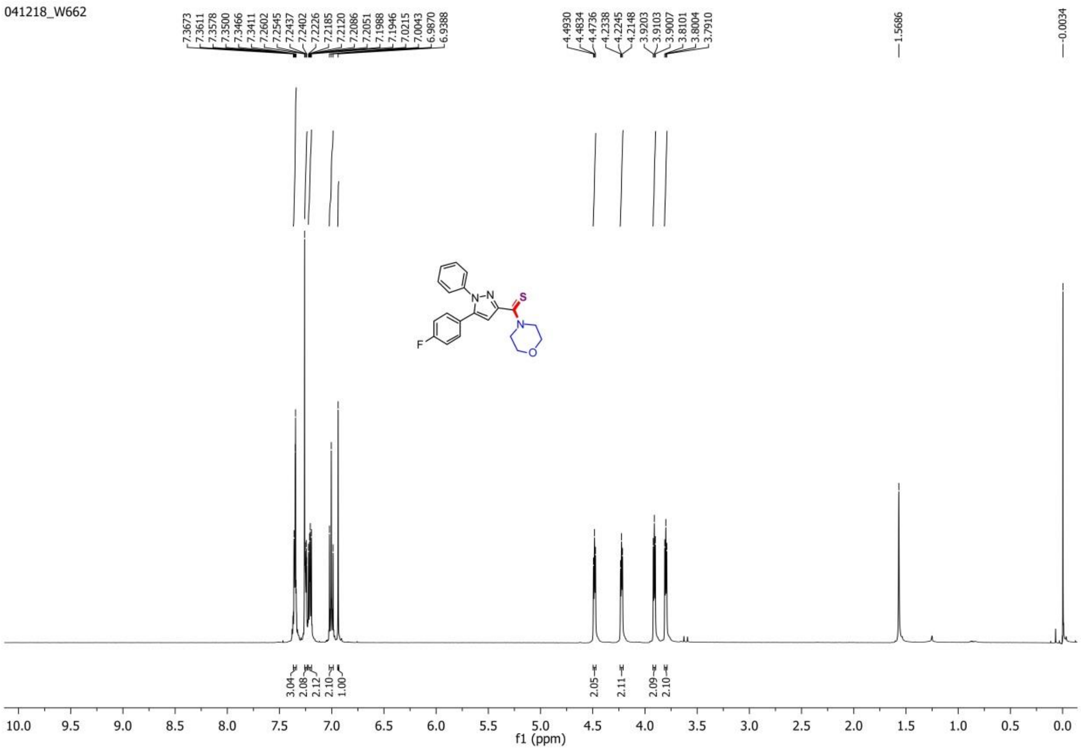

Figure S5: ^{1}H NMR spectrum of **1C**.

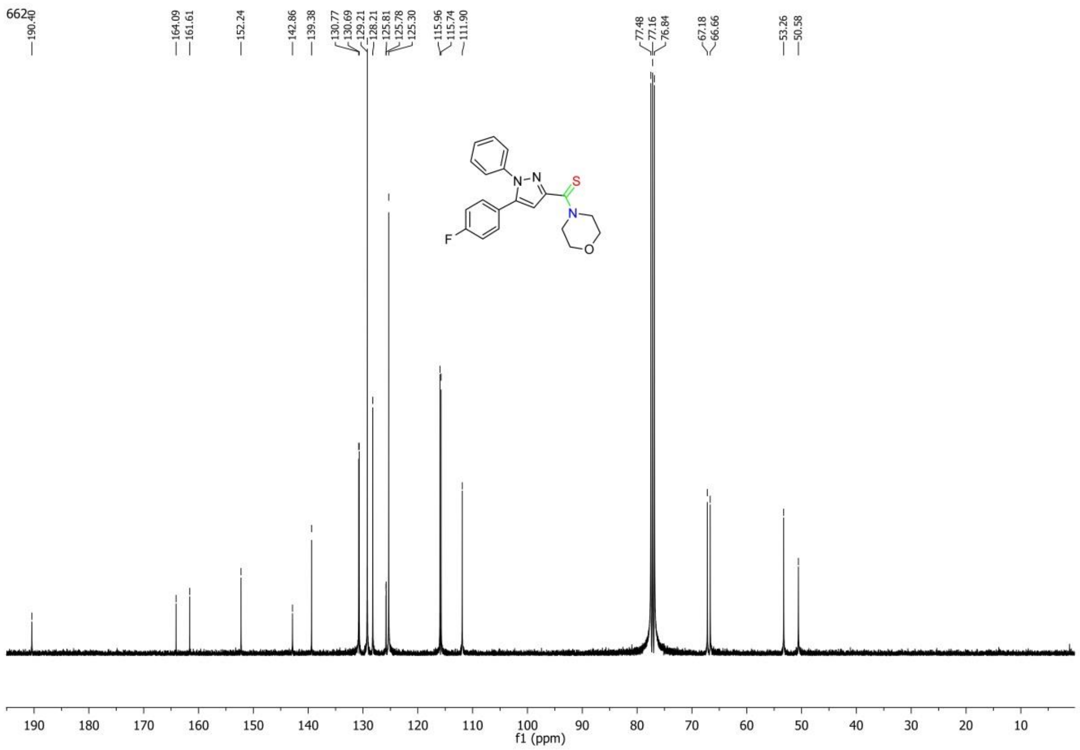

Figure S6: ^{13}C NMR spectrum of **1C**.

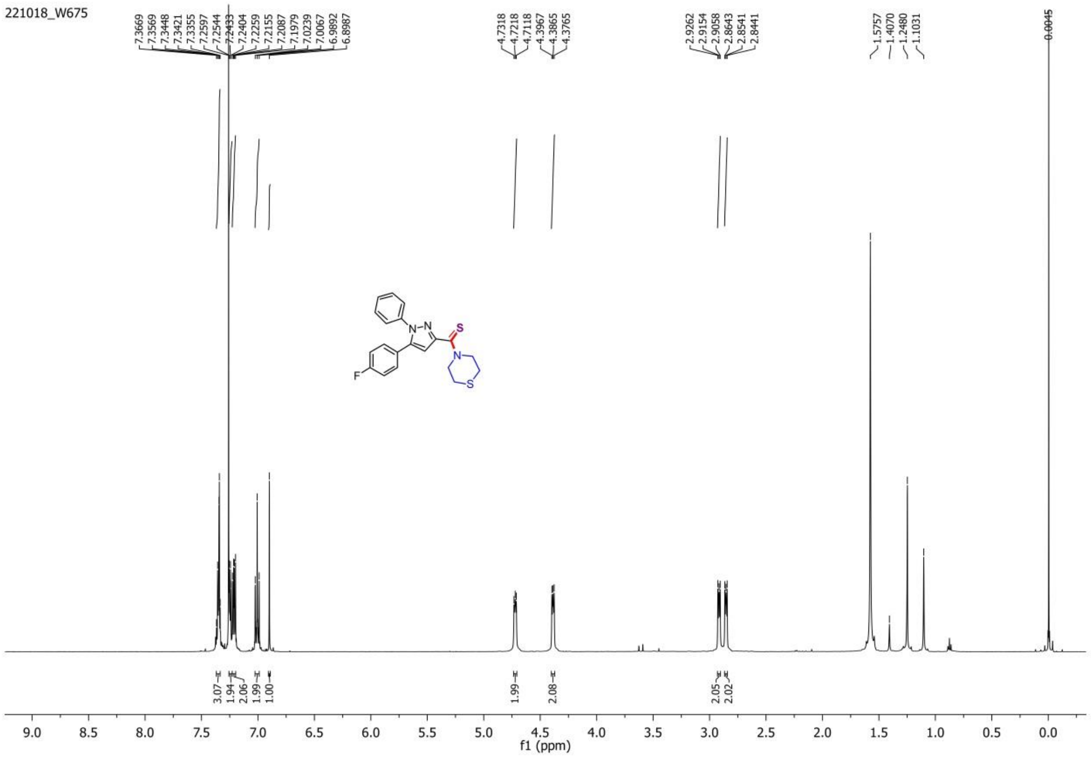

Figure S7: ^{1}H NMR spectrum of **1D**.

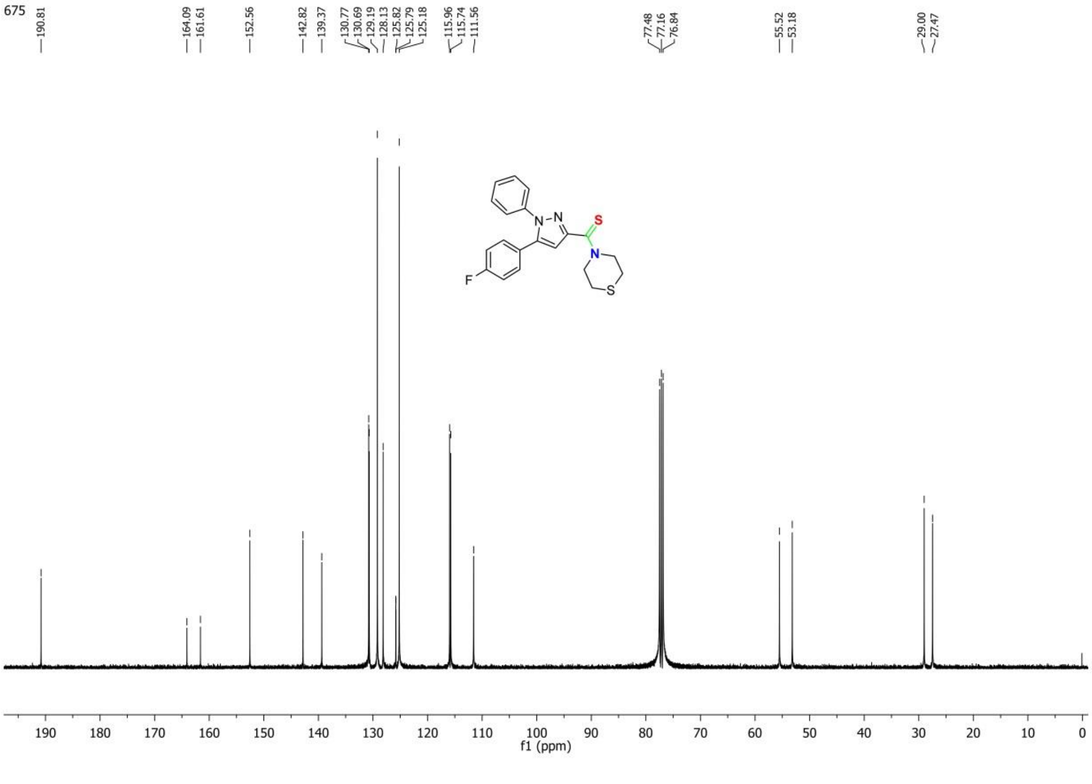

Figure S8: ^{13}C NMR spectrum of **1D**.

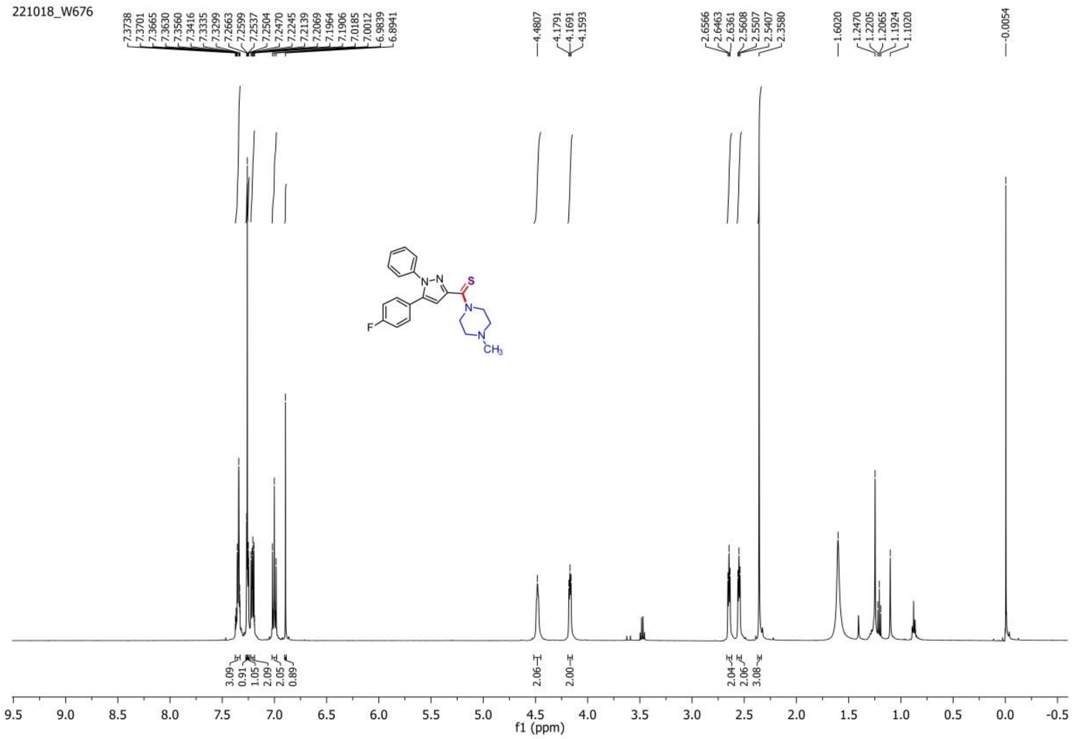

Figure S9: ^{1}H NMR spectrum of **1E**.

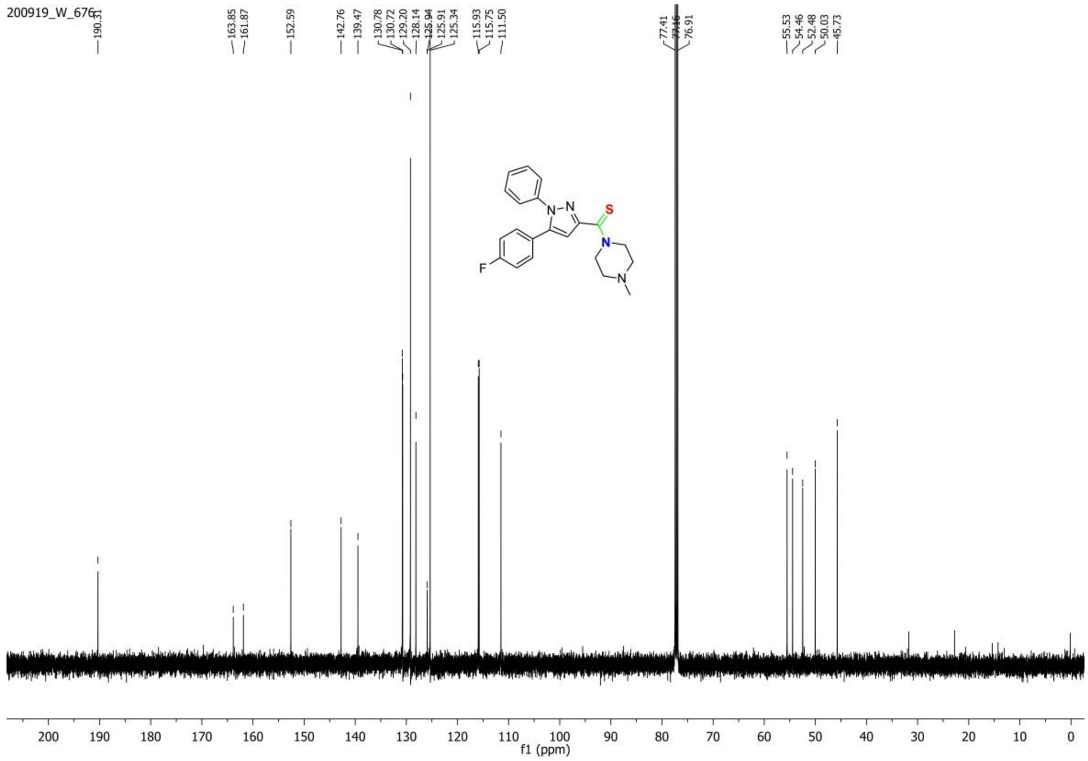

Figure S10: ^{13}C NMR spectrum of **1E**.

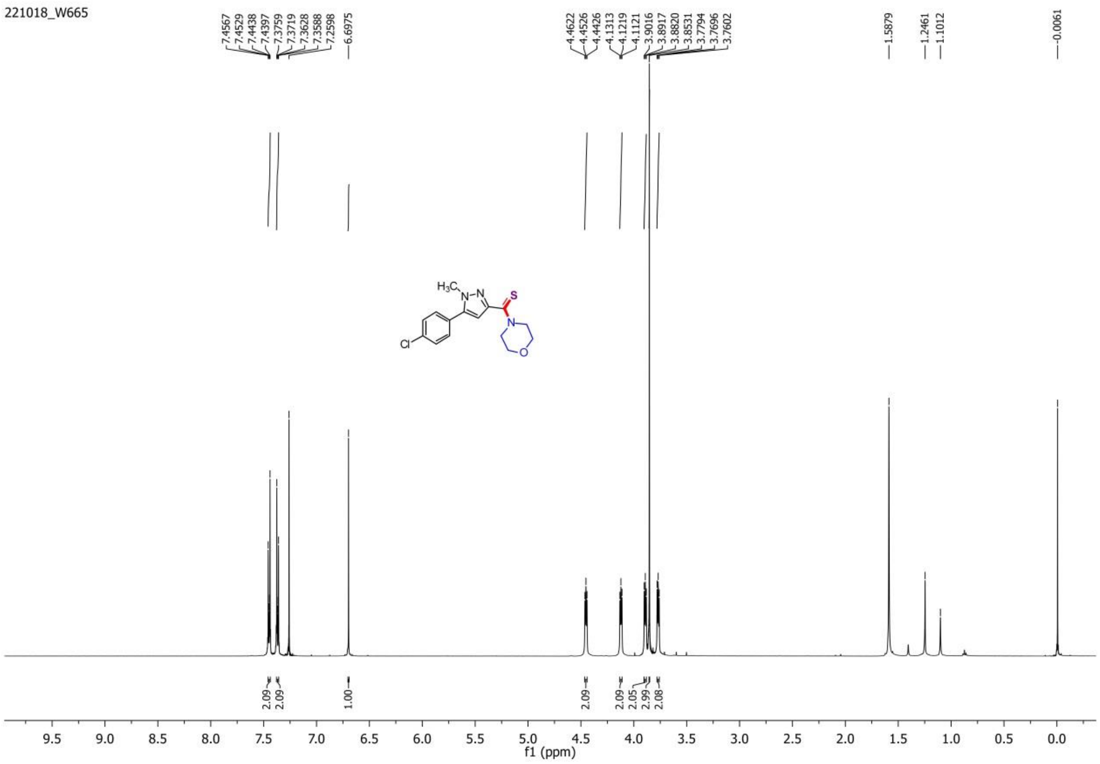

Figure S11: ^{1}H NMR spectrum of **2C**.

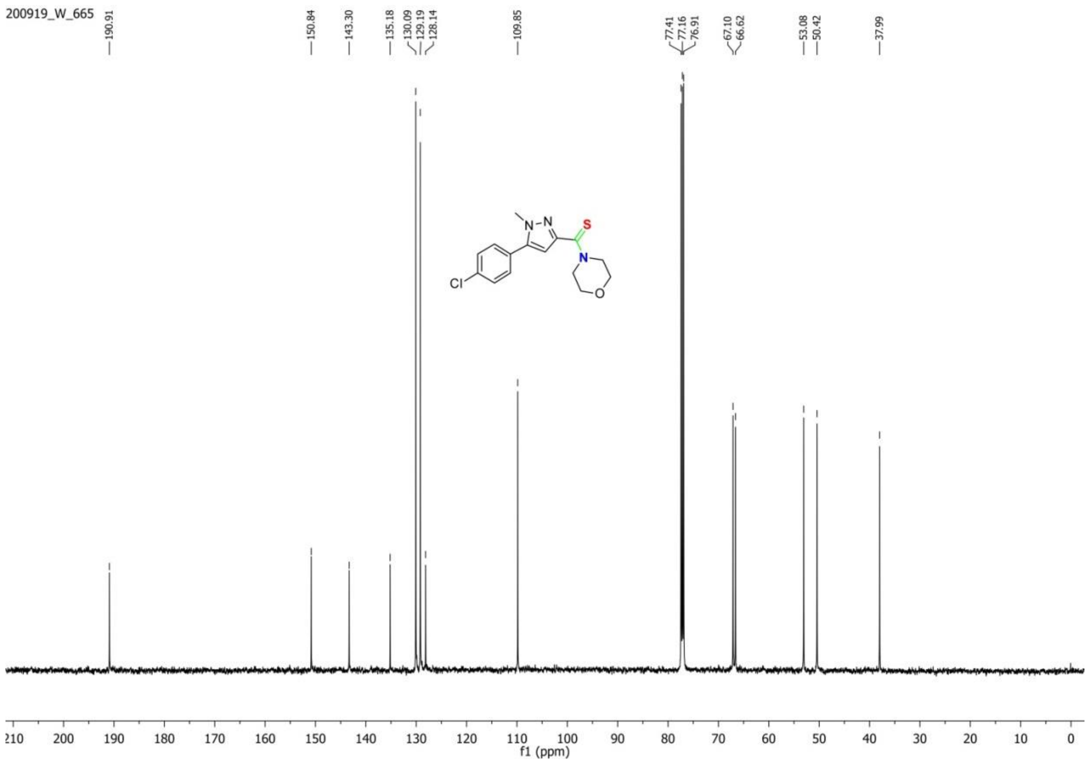

Figure S12: ^{13}C NMR spectrum of **2C**.

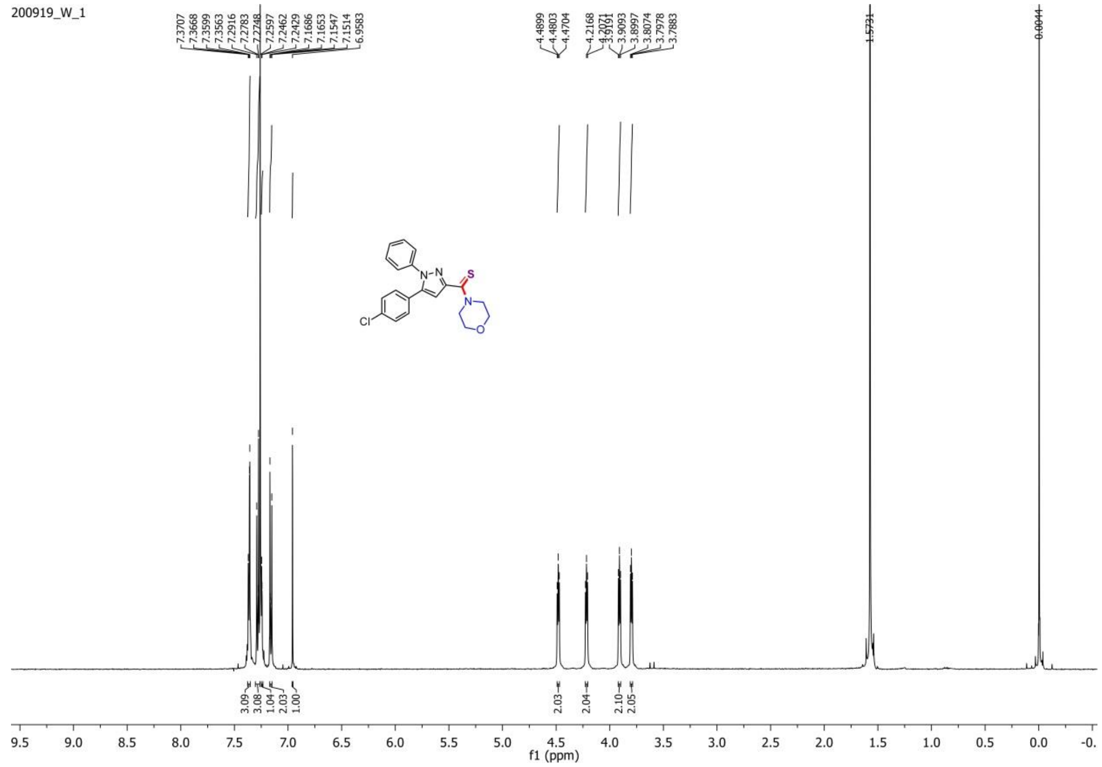

Figure S13: ^{1}H NMR spectrum of **3C**.

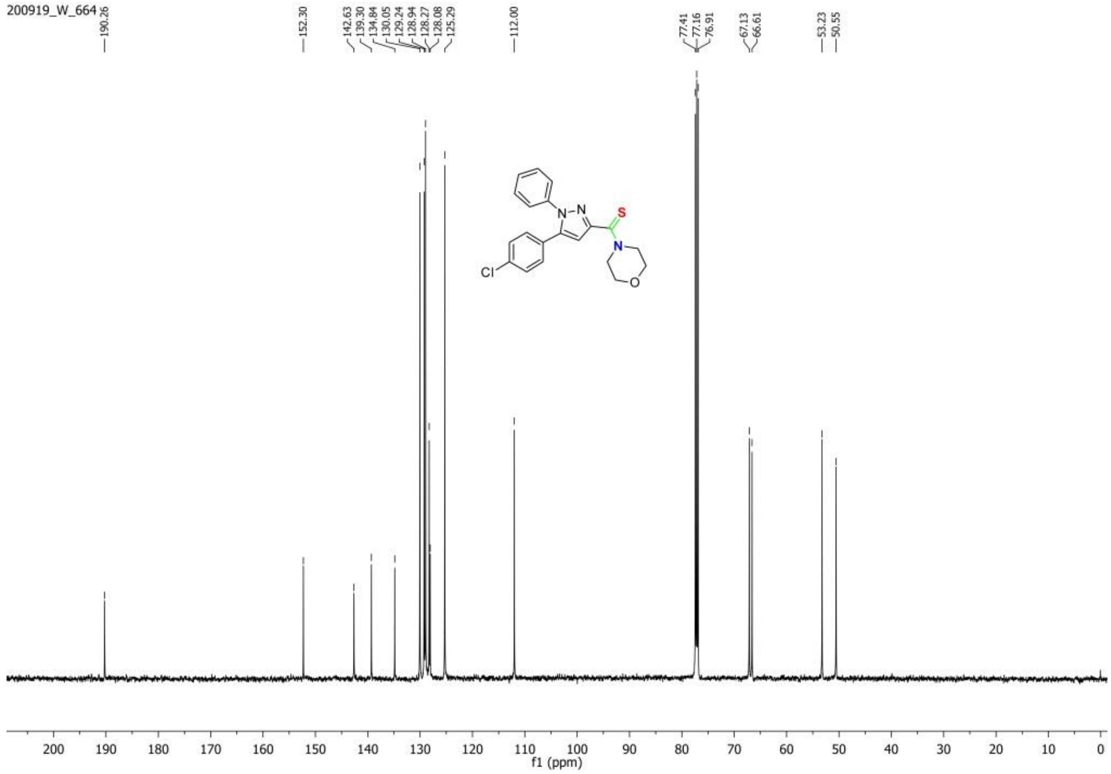

Figure S14: ^{13}C NMR spectrum of **3C**.

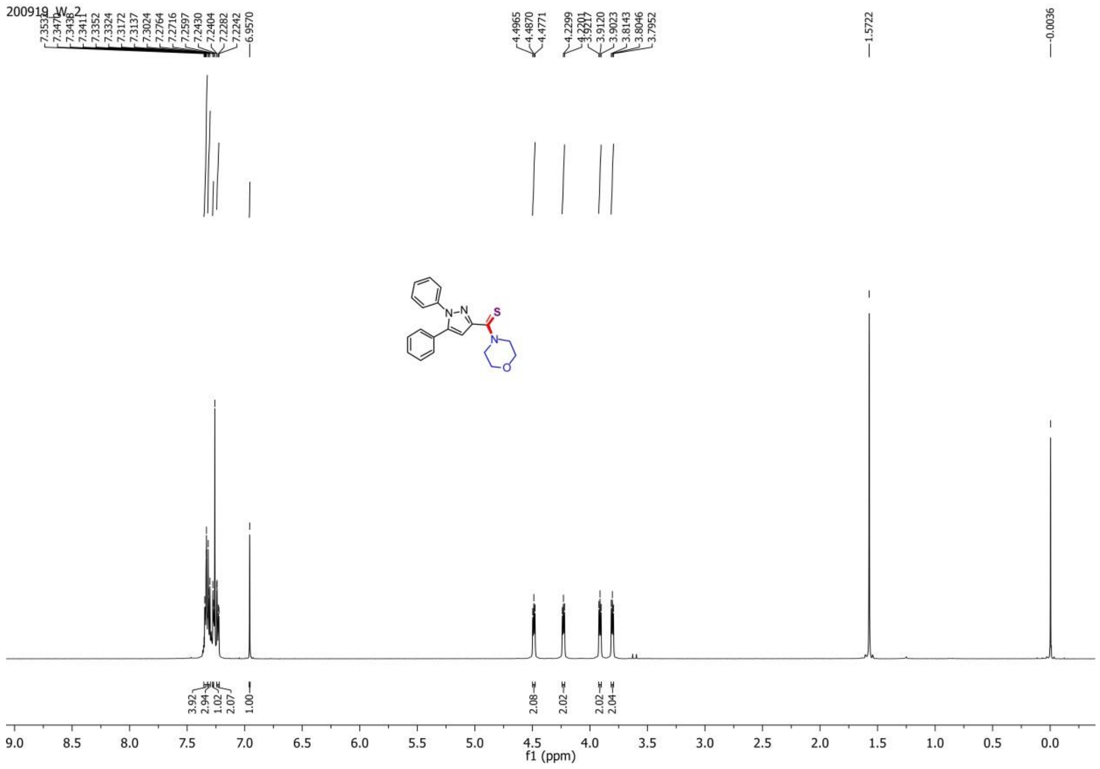

Figure S15: ^{1}H NMR spectrum of **4C**.

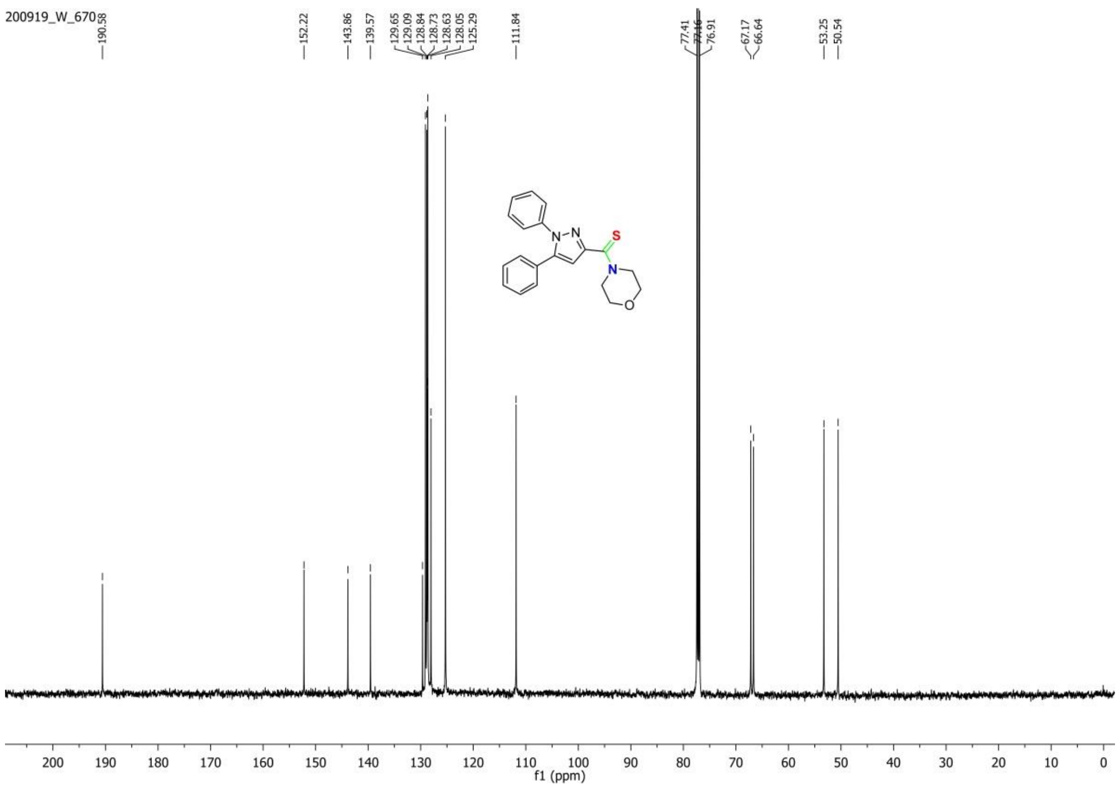

Figure S16: ^{13}C NMR spectrum of **4C**.

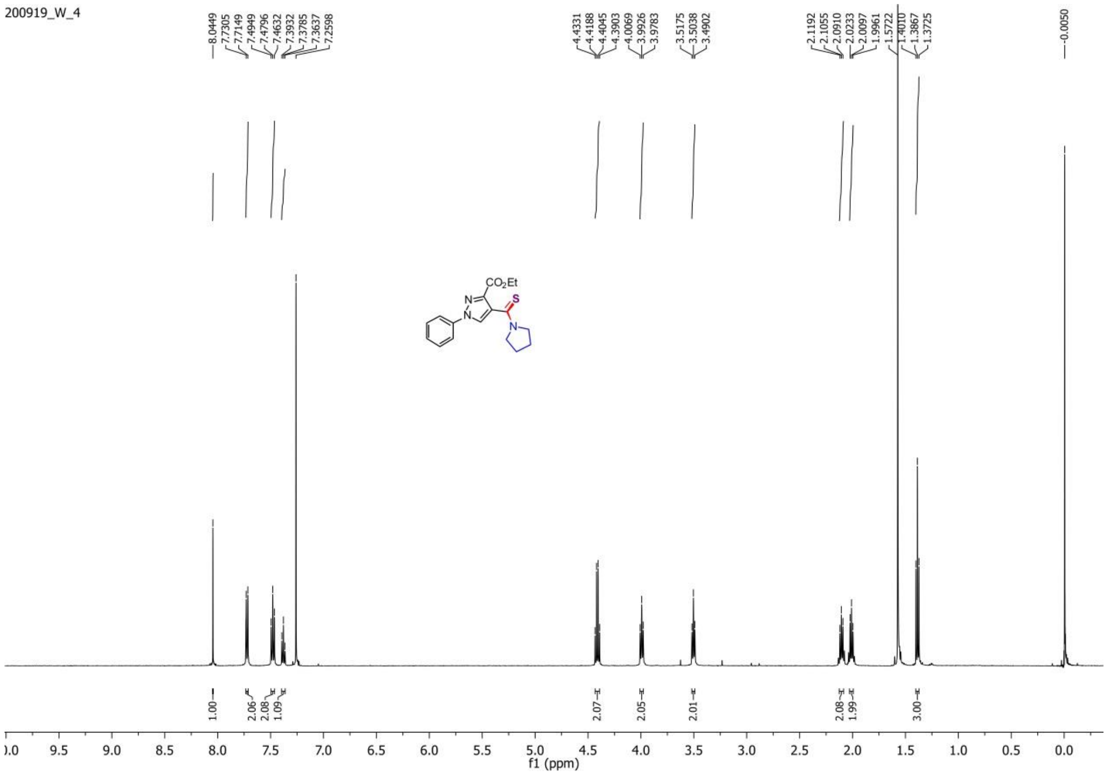

Figure S17: ^{1}H NMR spectrum of **5A**.

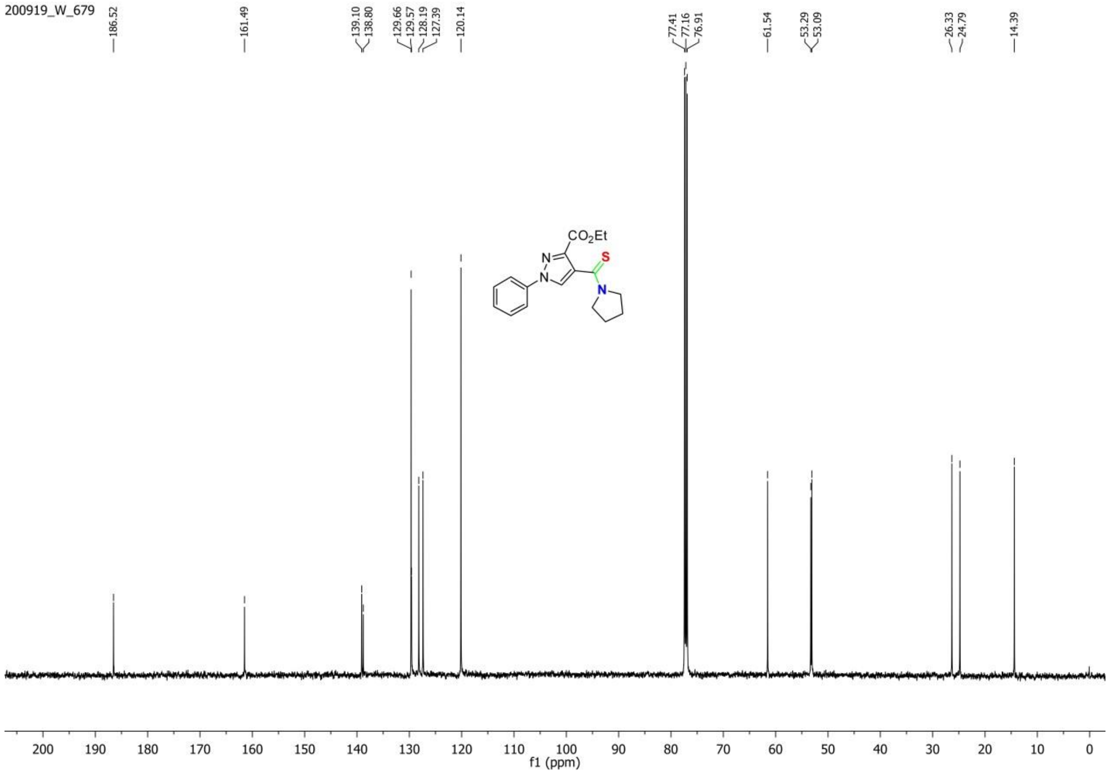

Figure S18: ^{13}C NMR spectrum of **5A**.

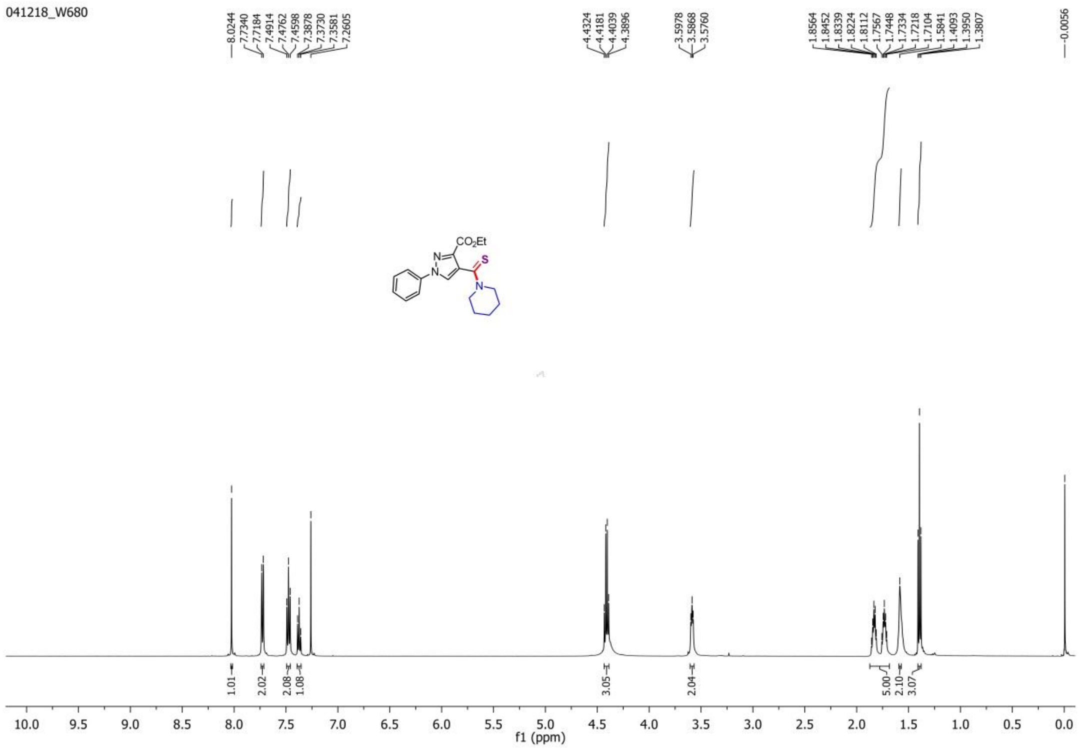

Figure S19: ^{1}H NMR spectrum of **5B**.

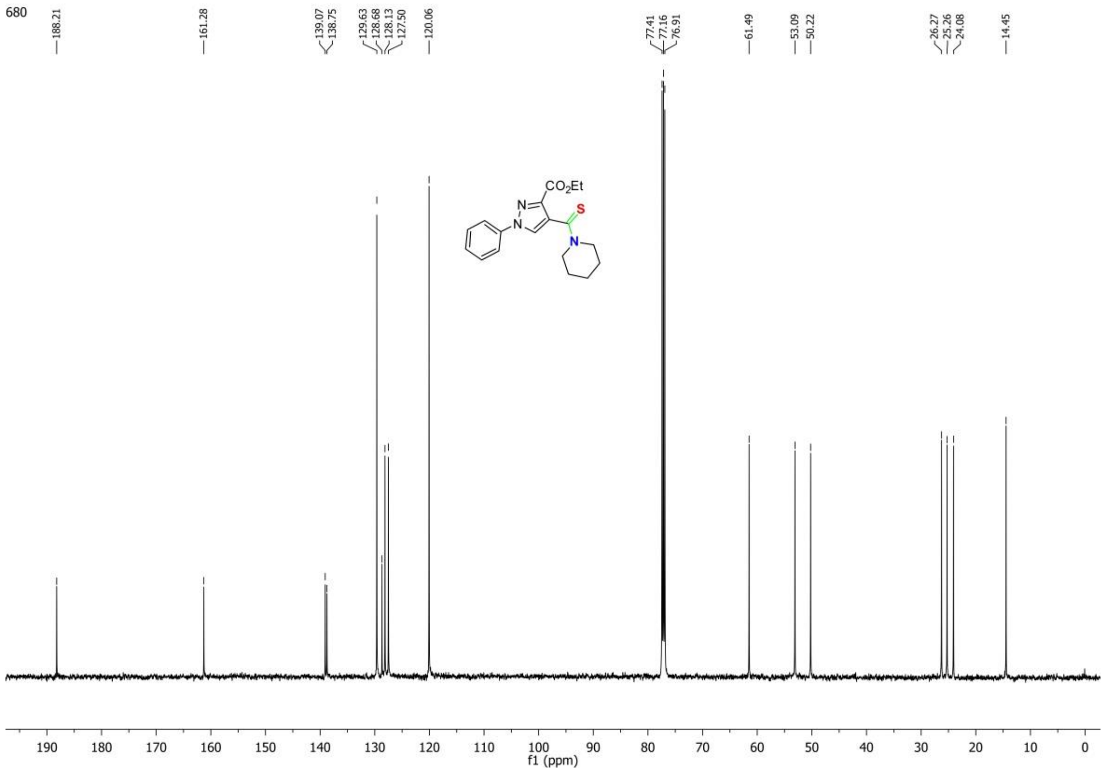

Figure S20: ^{13}C NMR spectrum of **5B**.

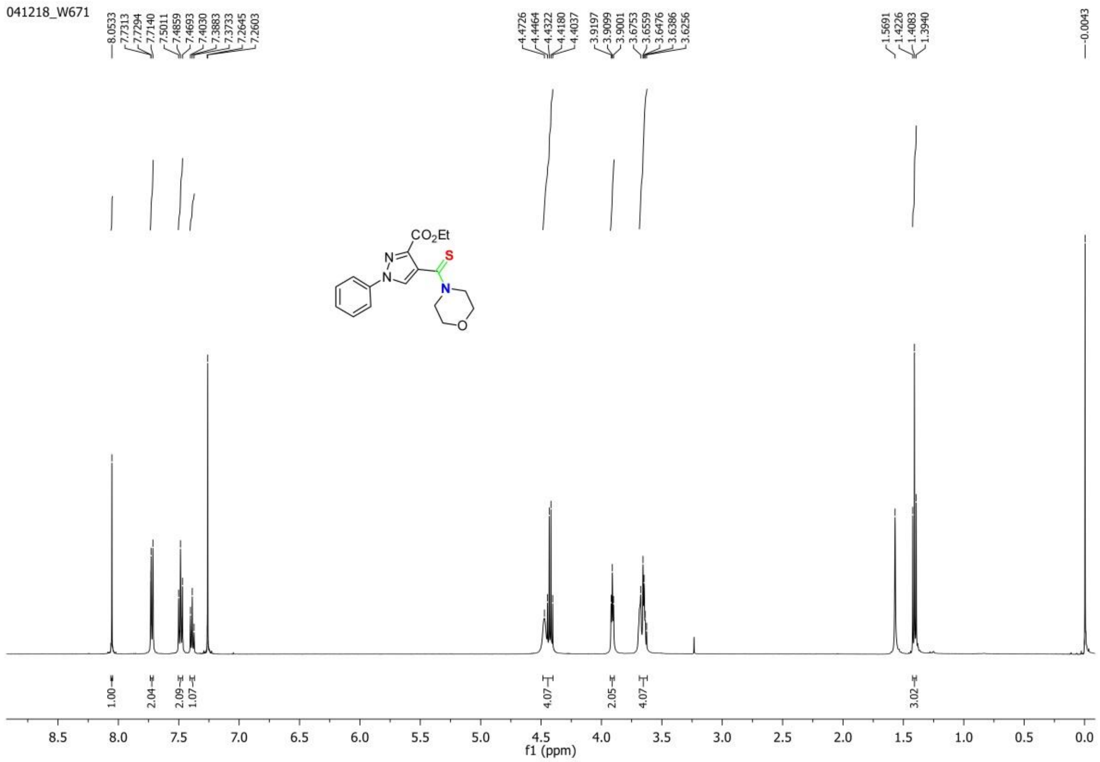

Figure S21: ^{1}H NMR spectrum of **5C**.

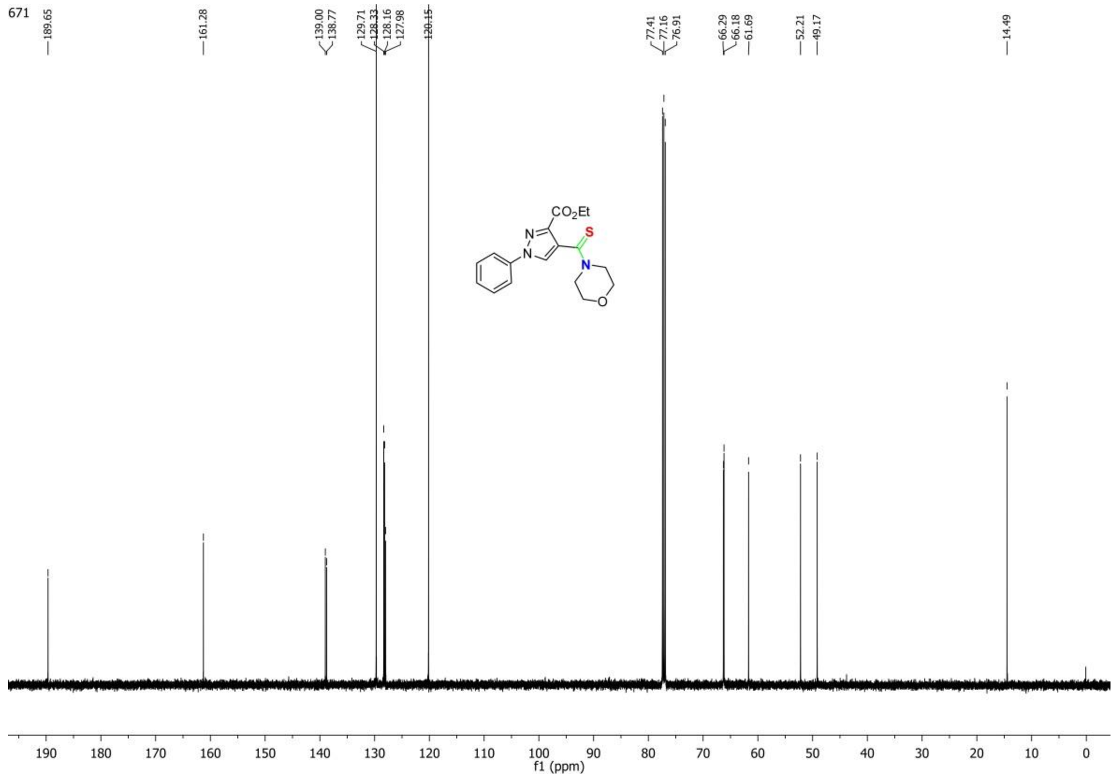

Figure S22: ^{13}C NMR spectrum of **5C**.

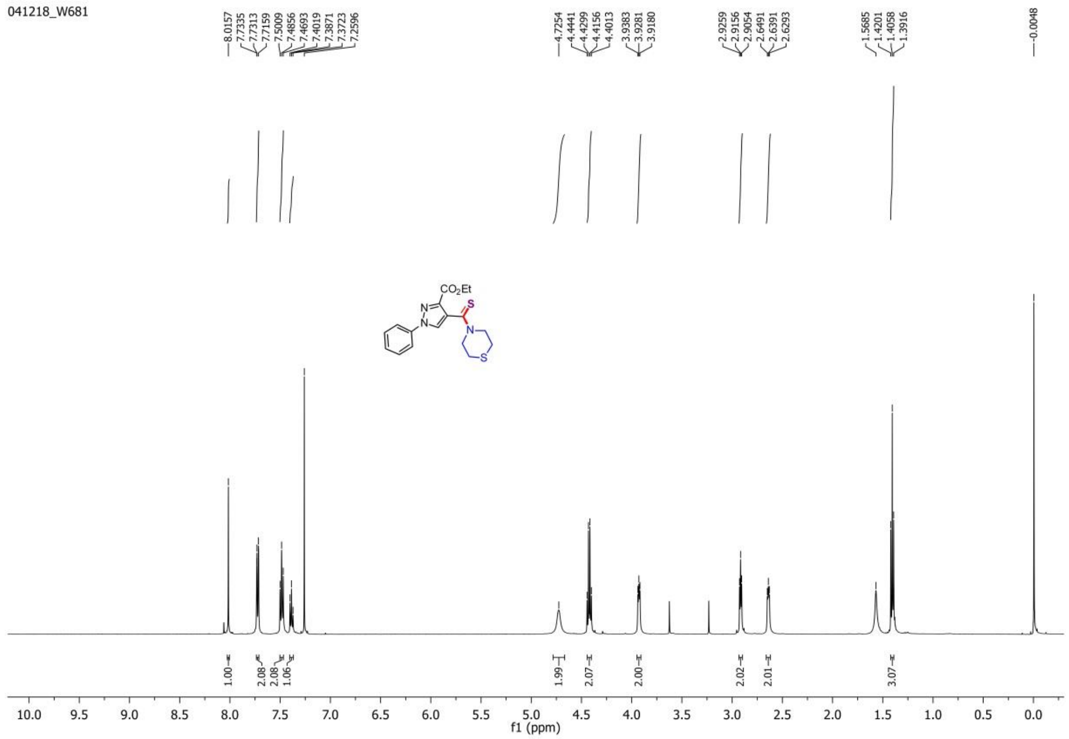

Figure S23: ^{1}H NMR spectrum of **5D**.

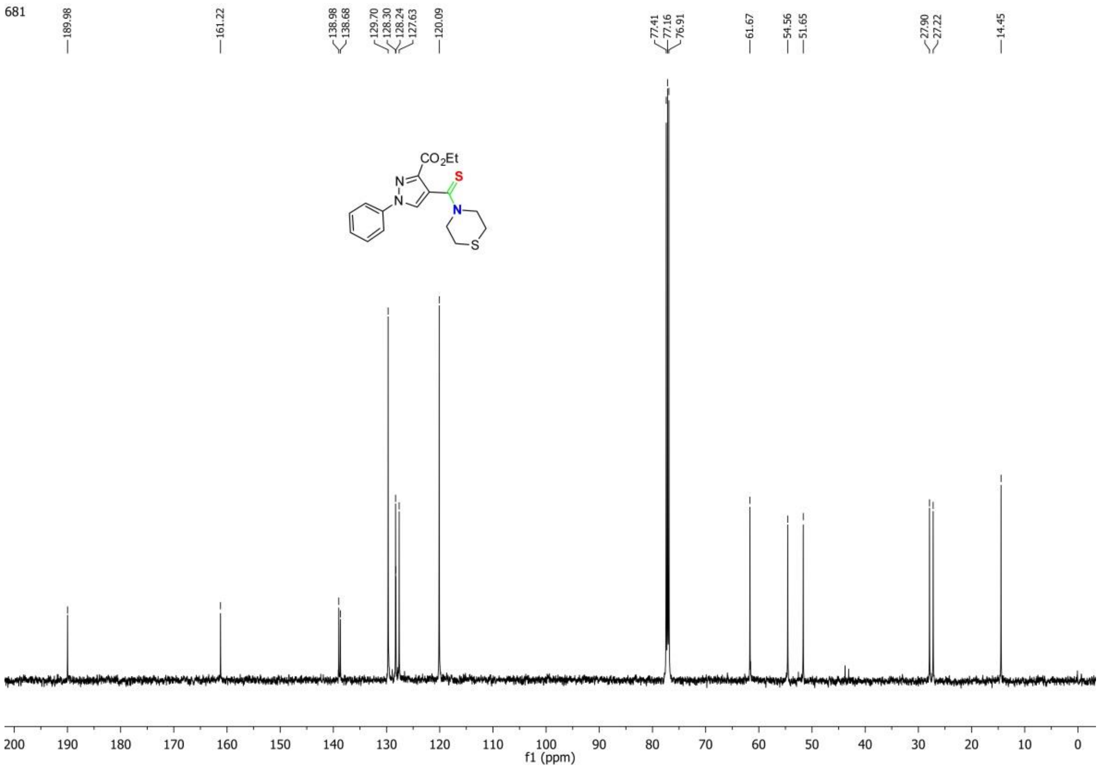

Figure S24: ^{13}C NMR spectrum of **5D**.

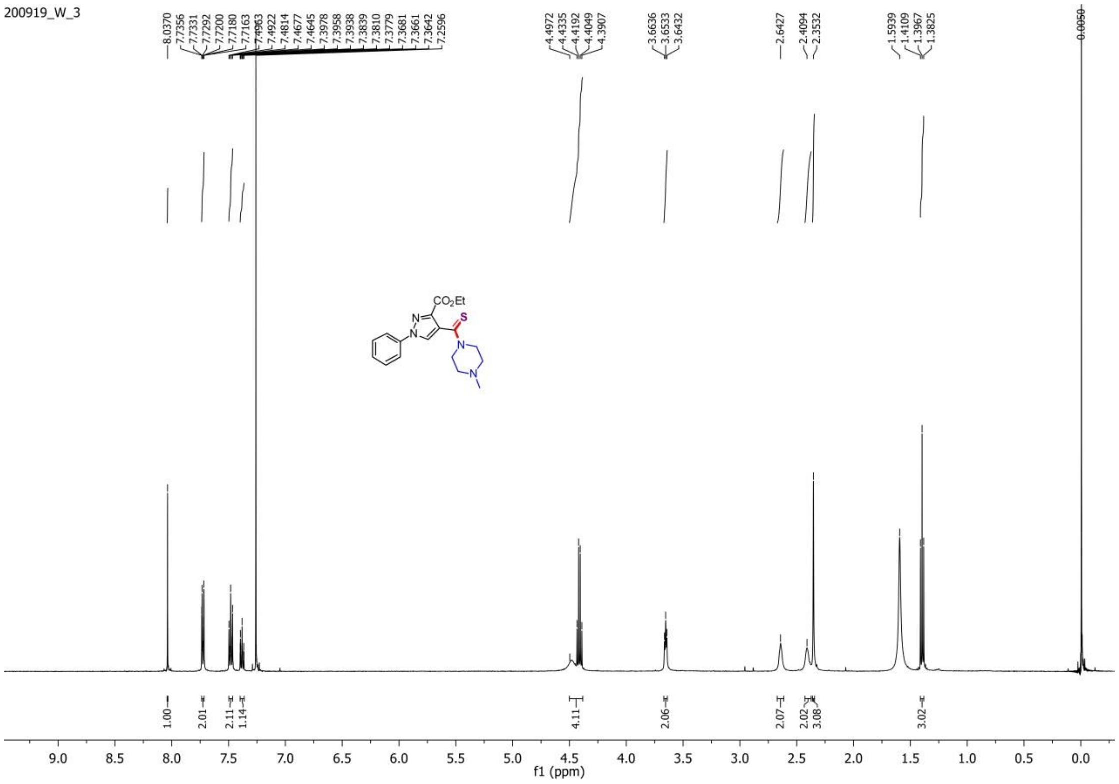

Figure S25: ^{1}H NMR spectrum of **5E**.

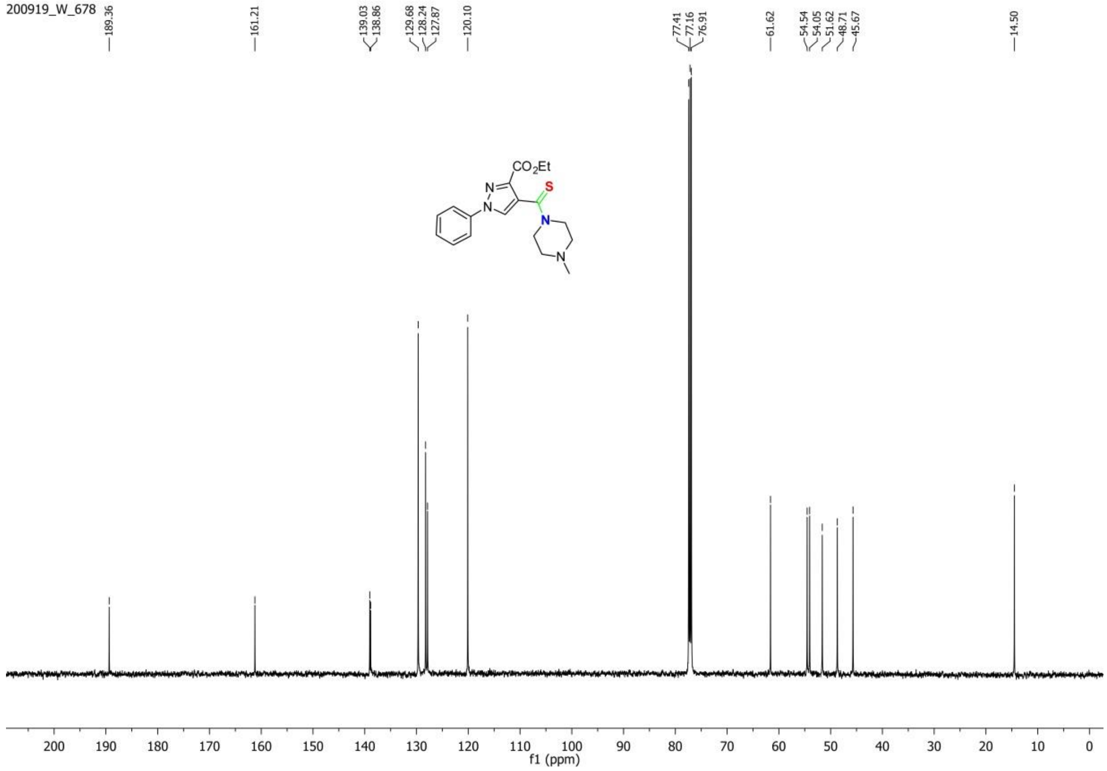

Figure S26: ^{13}C NMR spectrum of **5E**.

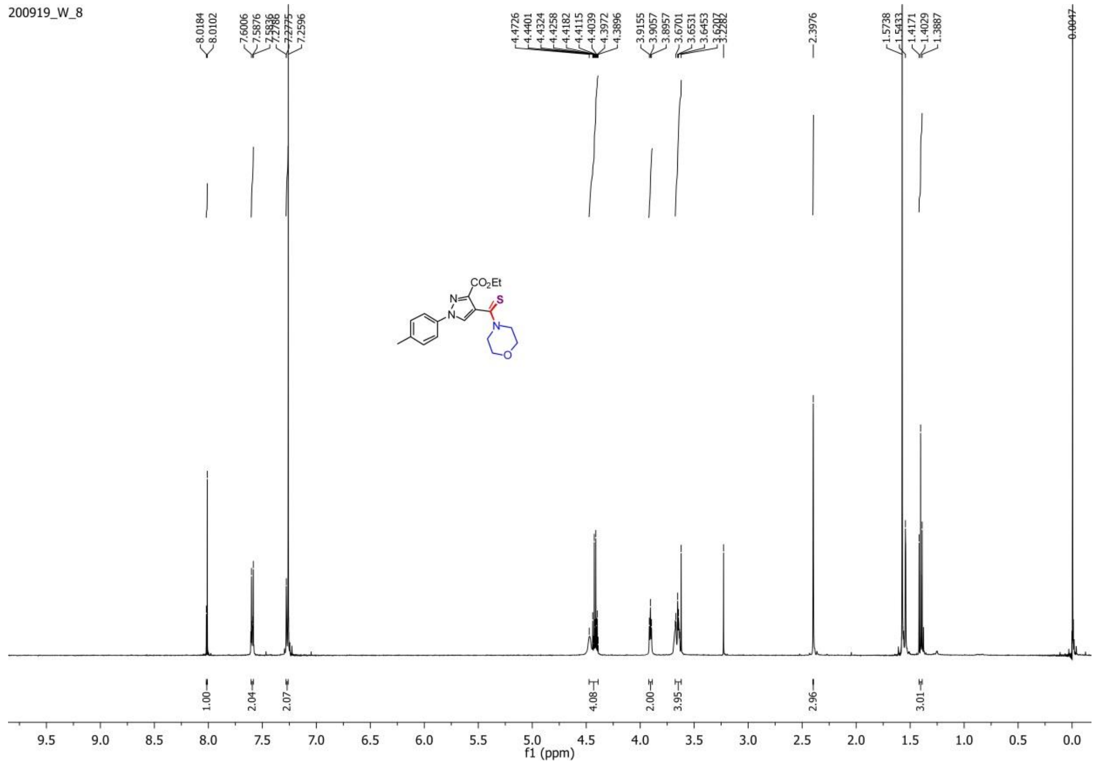

Figure S27: ^{1}H NMR spectrum of **6C**.

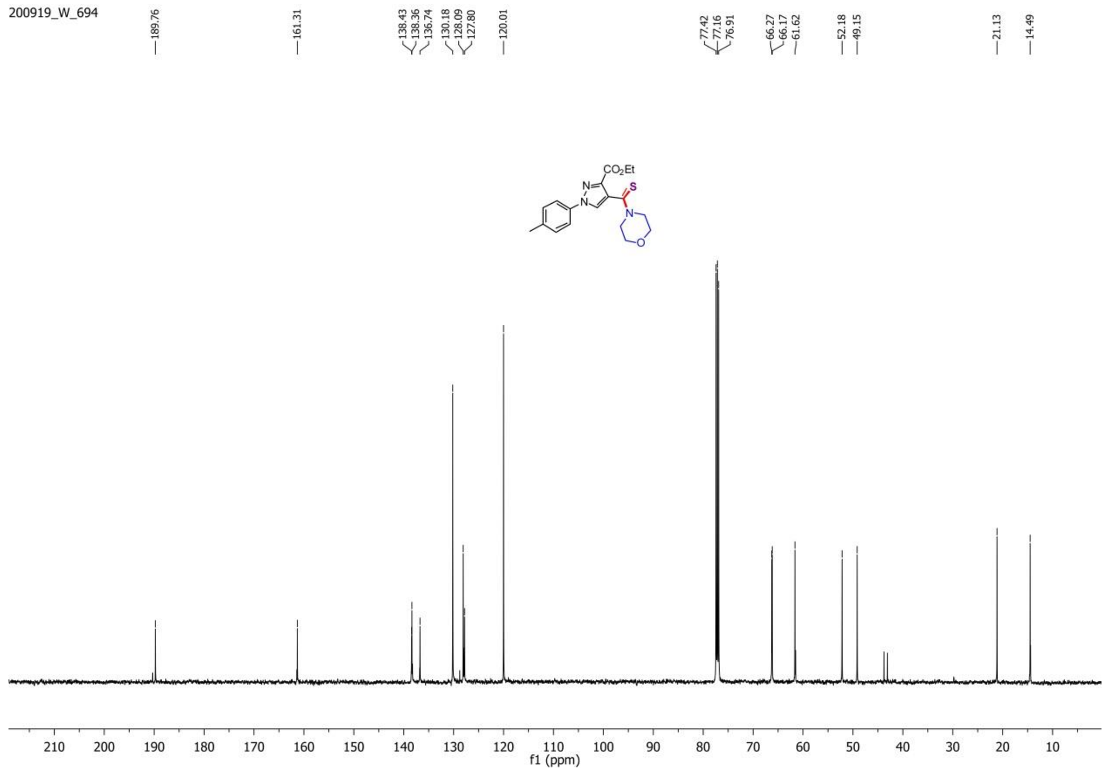

Figure S28: ^{13}C NMR spectrum of **6C**.

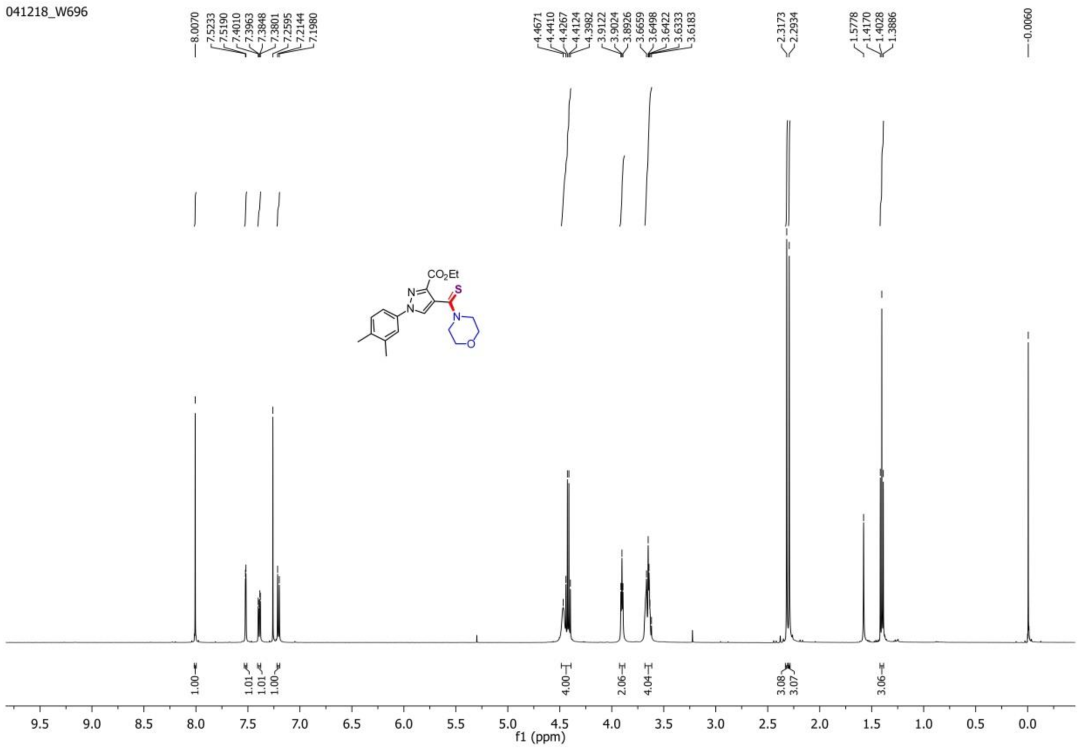

Figure S29: ^{1}H NMR spectrum of **7C**.

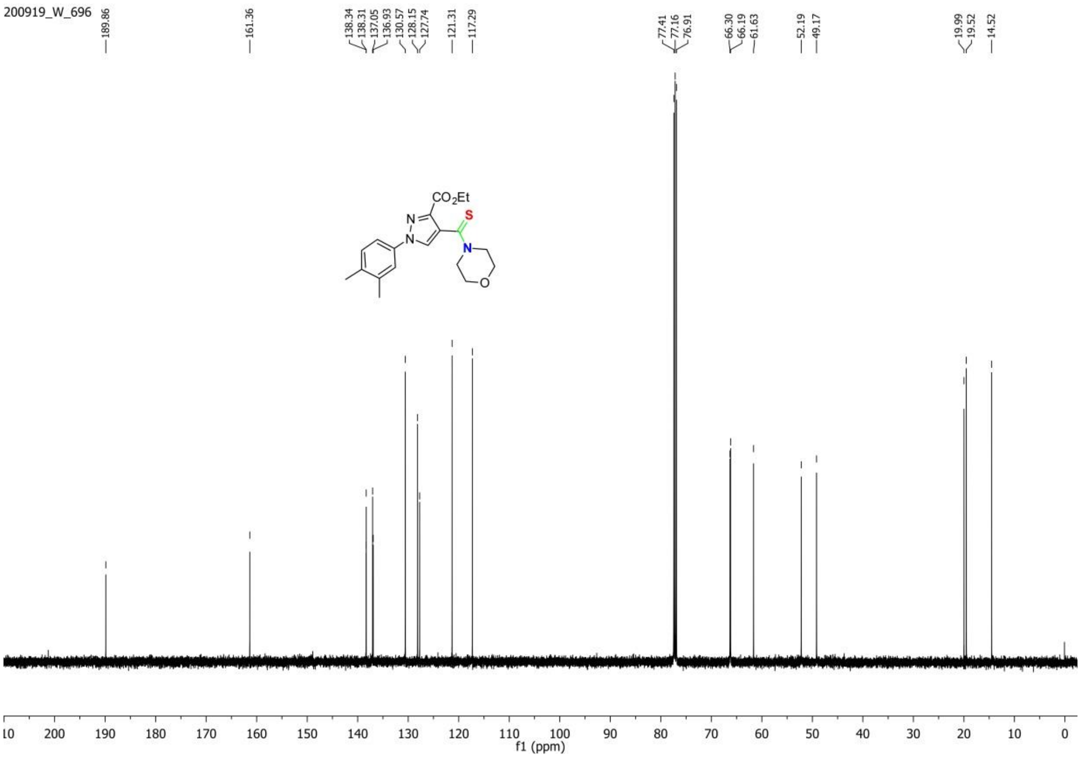

Figure S30: ^{13}C NMR spectrum of **7C**.

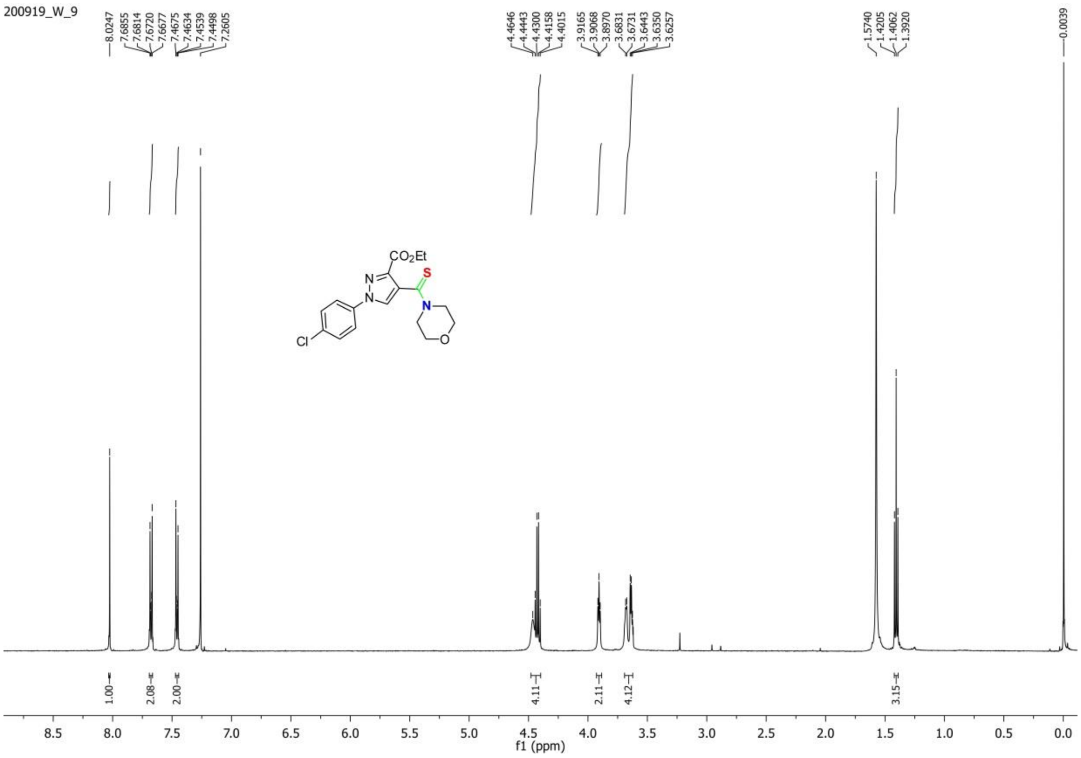

Figure S31: ^{1}H NMR spectrum of **8C**.

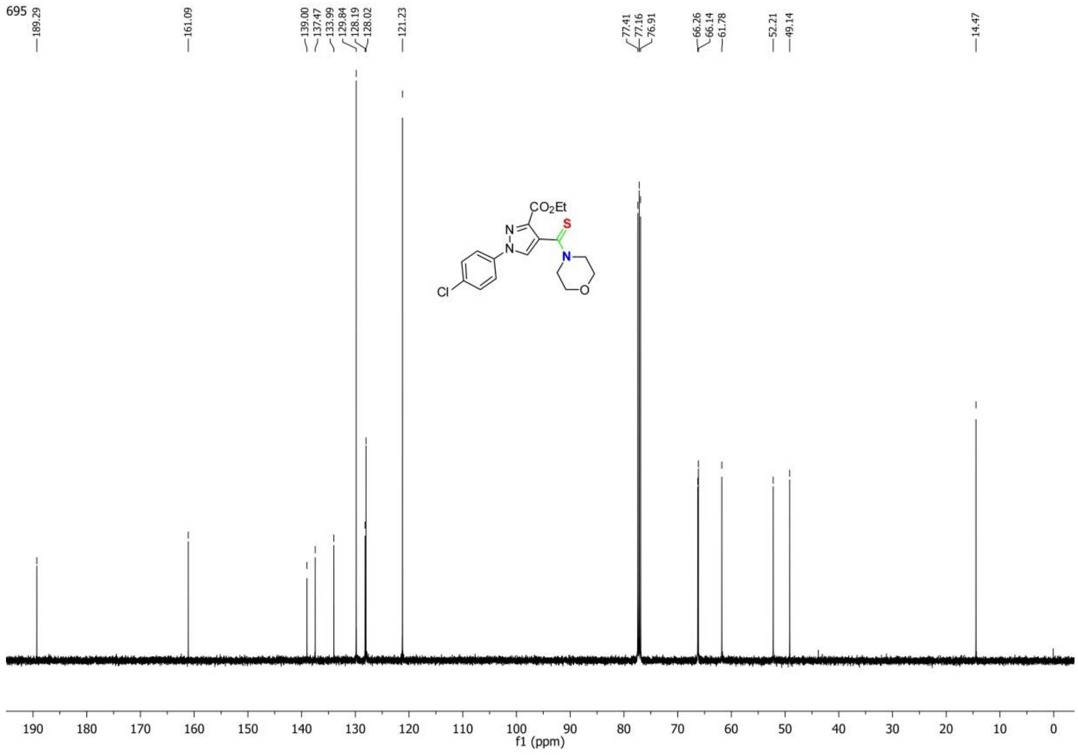

Figure S32: ^{13}C NMR spectrum of **8C**.

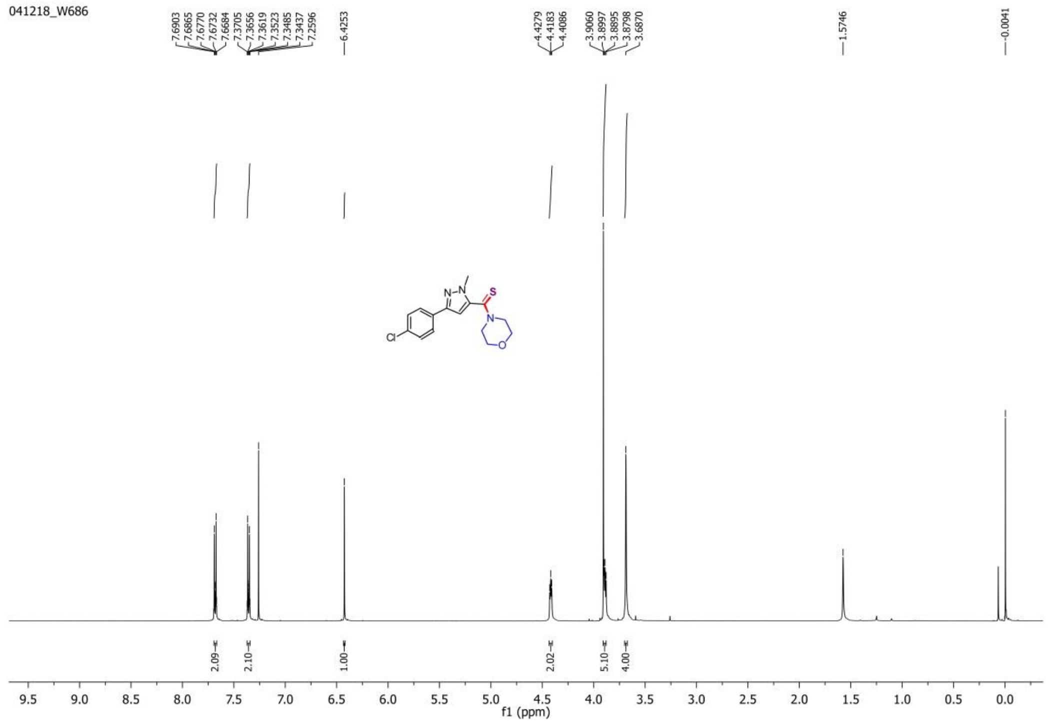

Figure S33: ^{1}H NMR spectrum of **9C**.

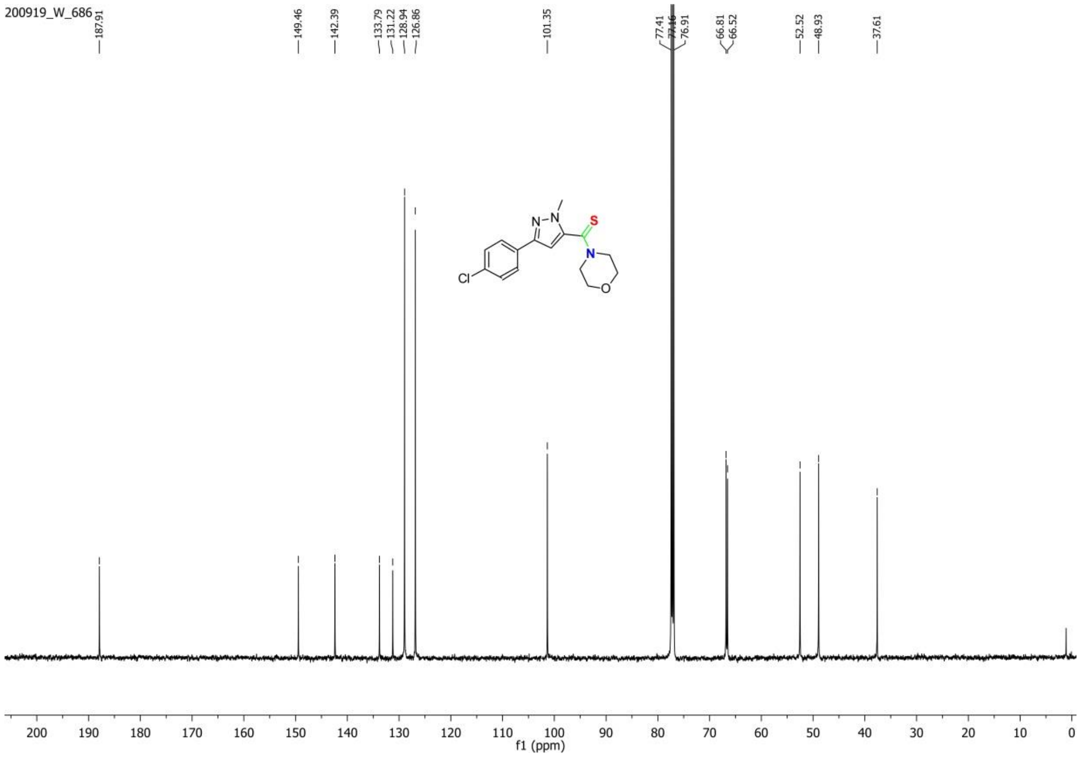

Figure S34: ^{13}C NMR spectrum of **9C**.

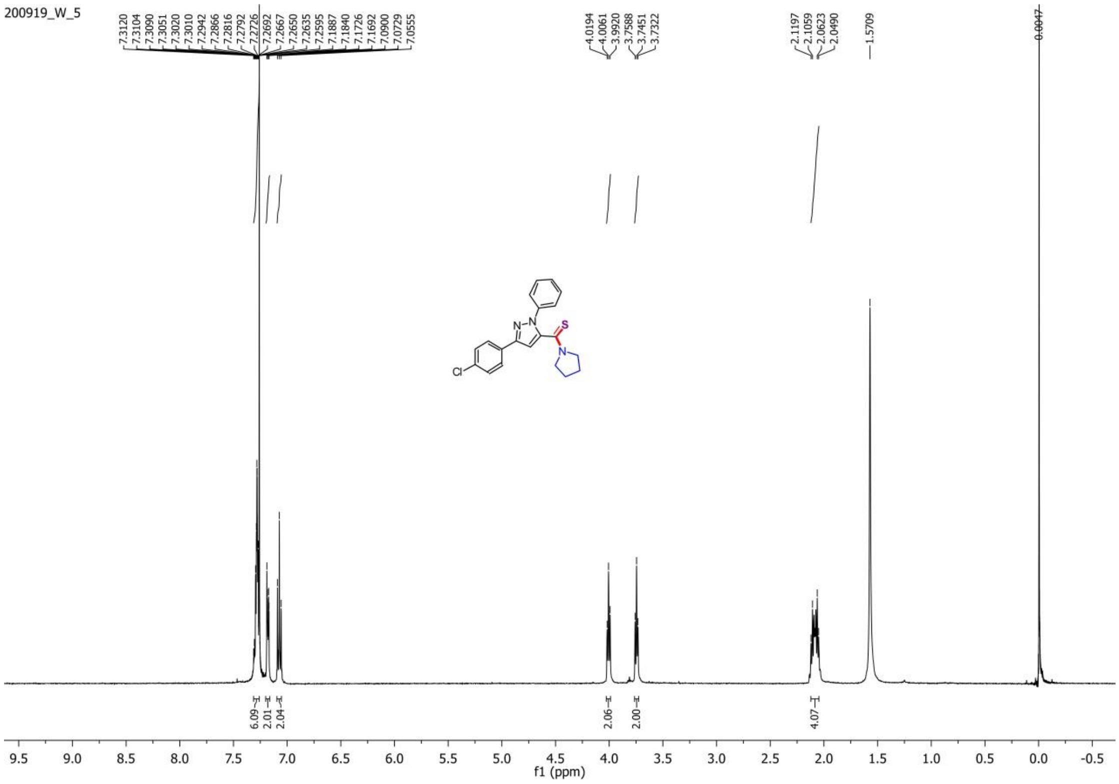

Figure S35: ^{1}H NMR spectrum of **10A**.

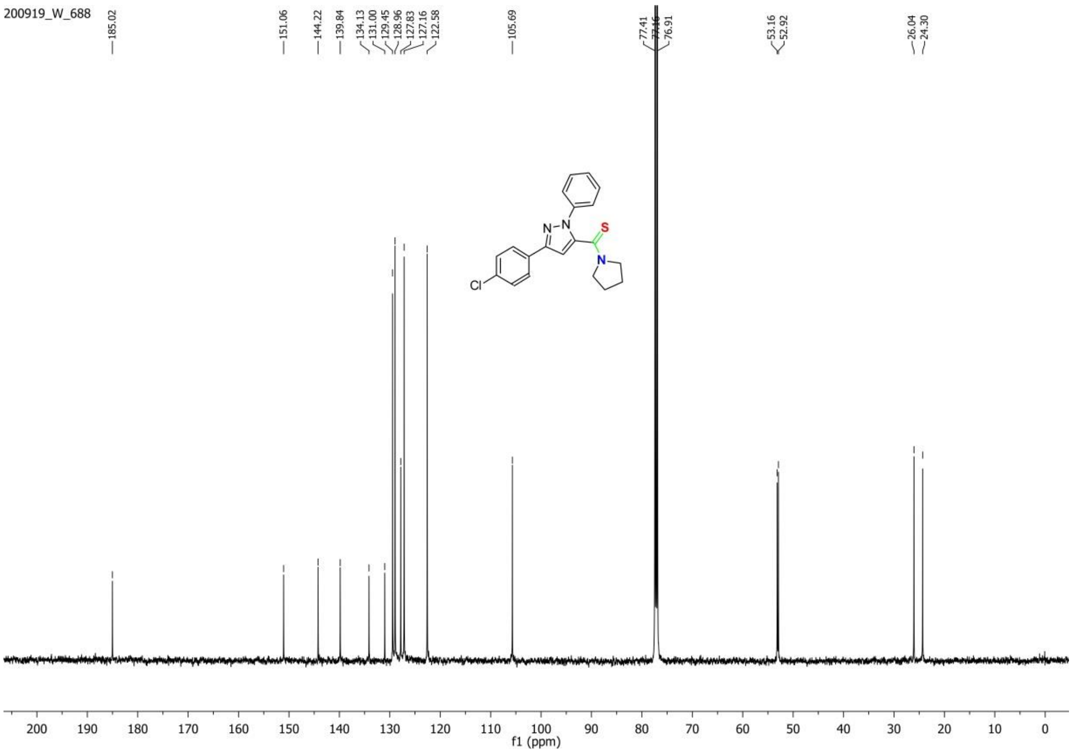

Figure S36: ^{13}C NMR spectrum of **10A**.

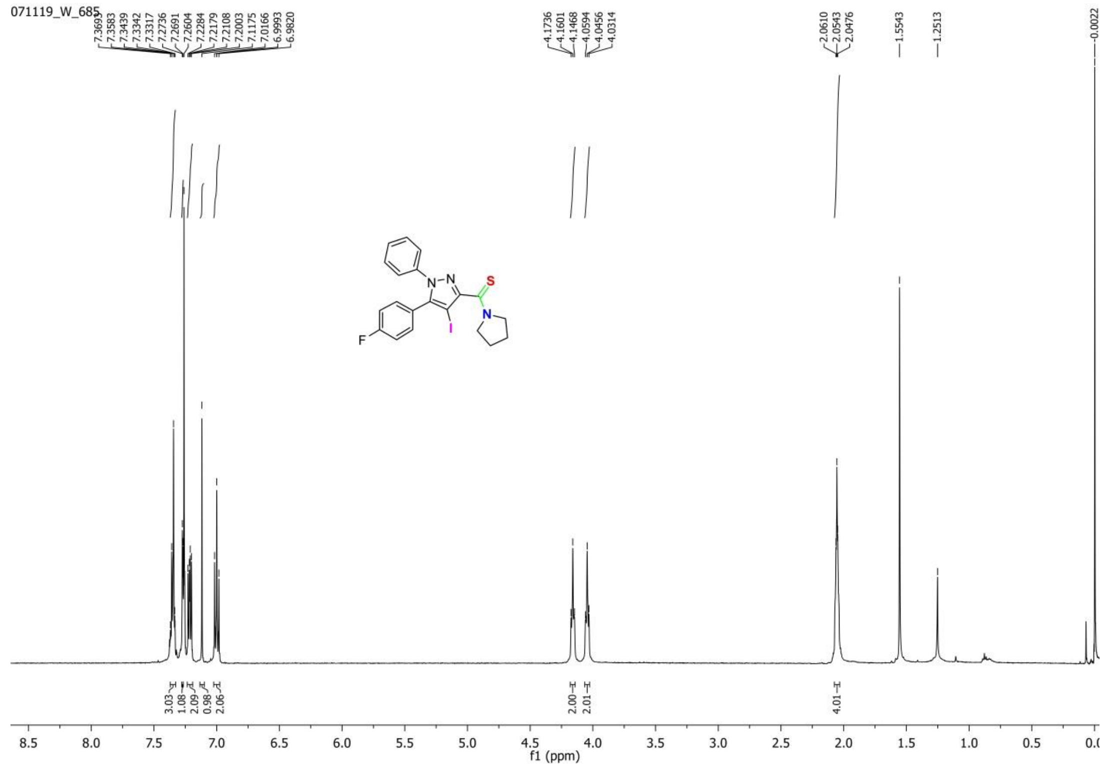

Figure S37: ^{1}H NMR spectrum of **11A**.

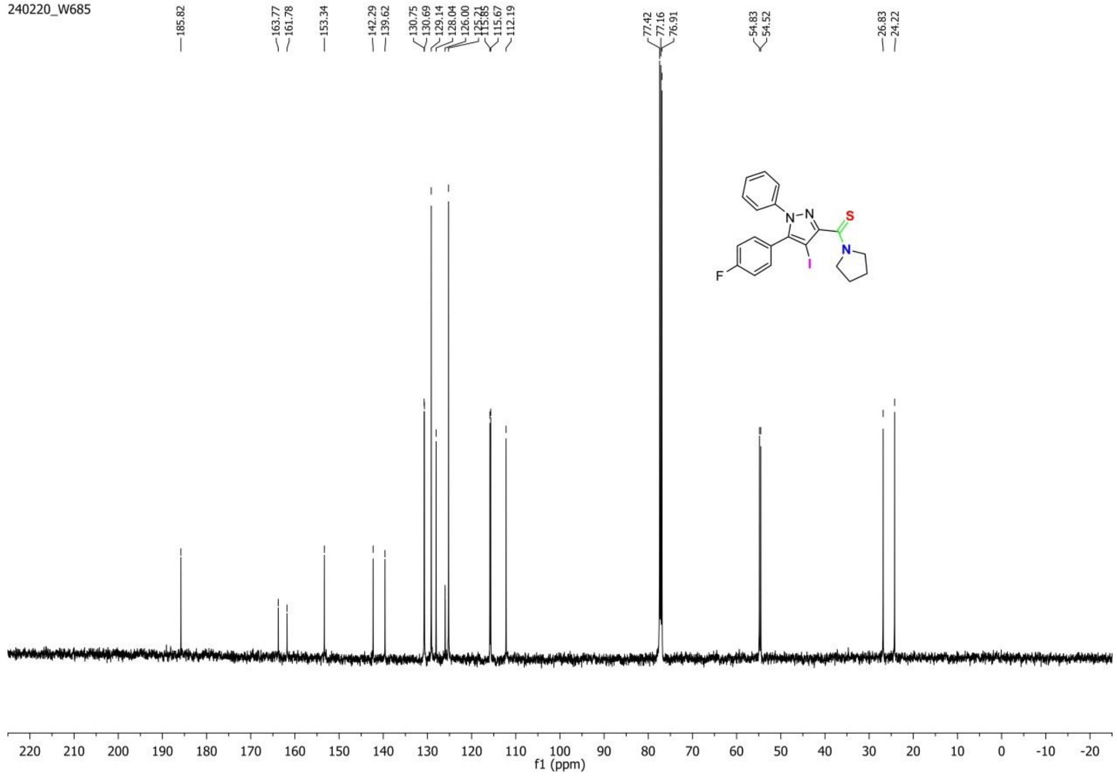

Figure S38: ^{13}C NMR spectrum of **11A**.

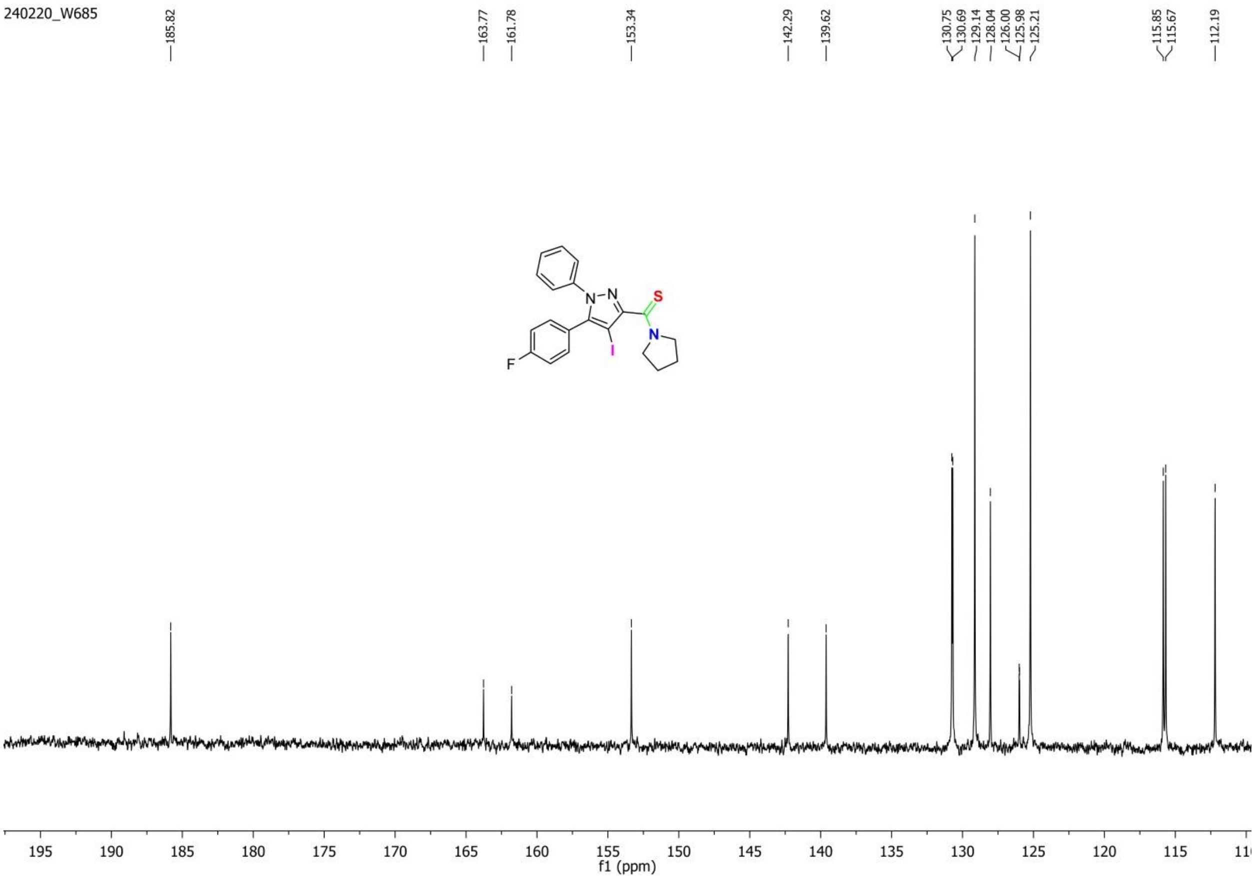

Figure S39: Expansion of ^{13}C NMR spectrum of **11A**.

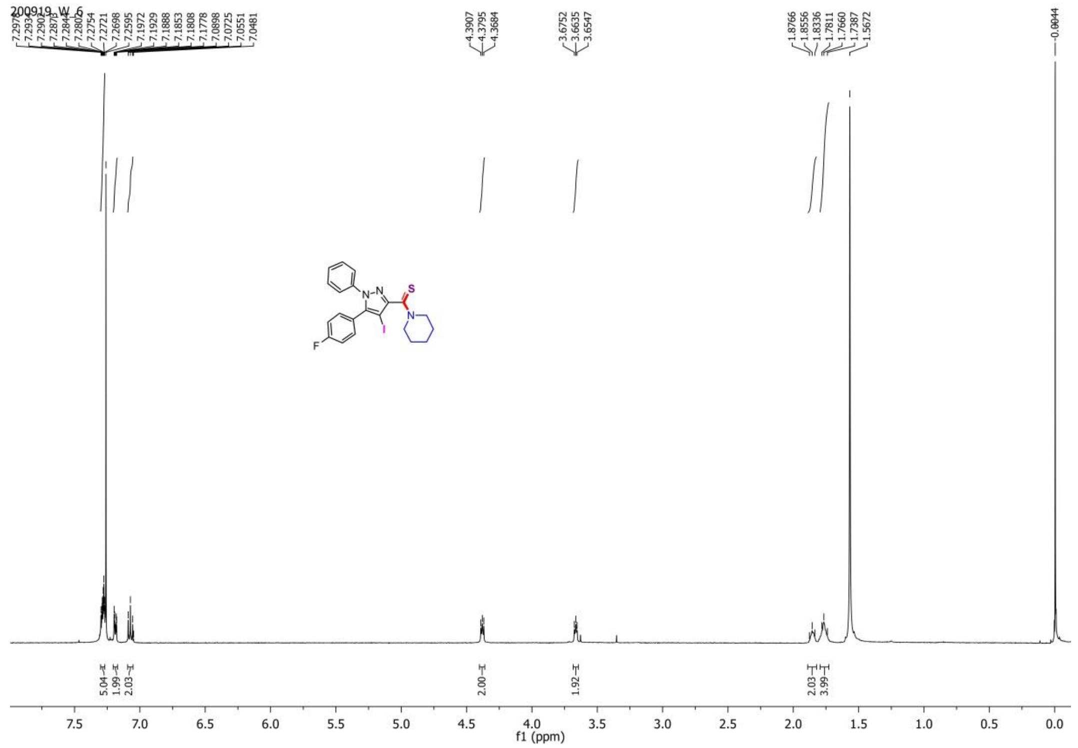

Figure S40: ^{1}H NMR spectrum of **11B**.

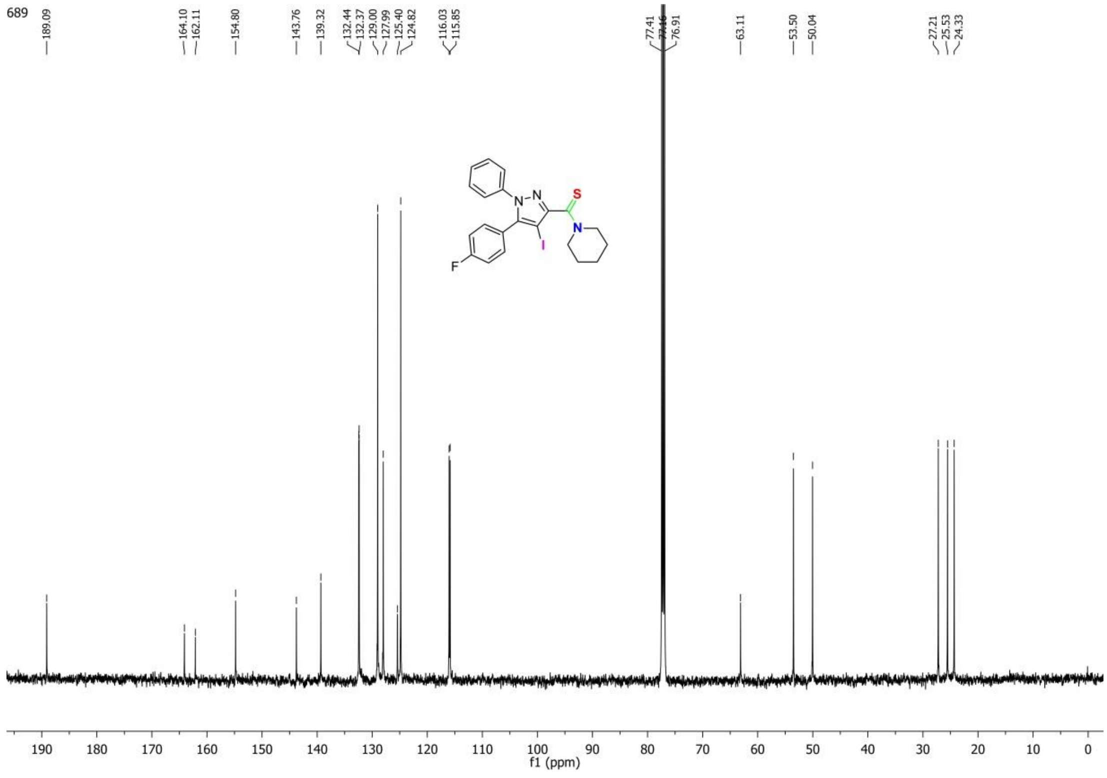

Figure S41: ^{13}C NMR spectrum of **11B**.

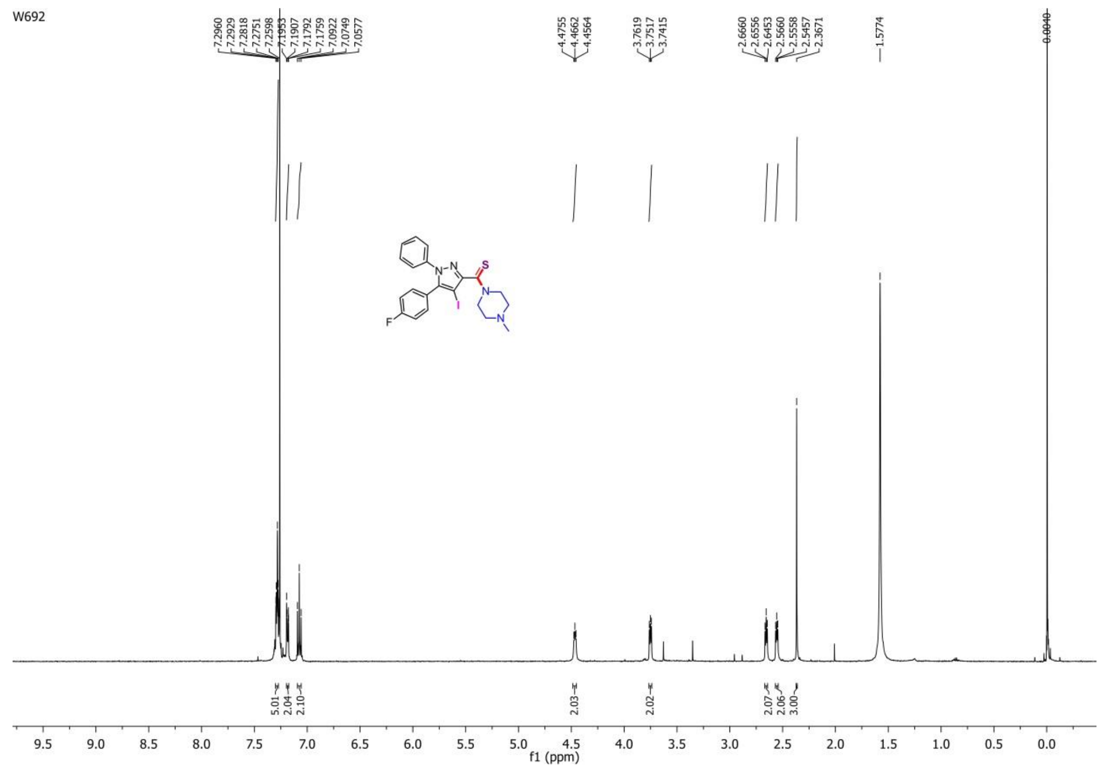

Figure S42: ^{1}H NMR spectrum of **11E**.

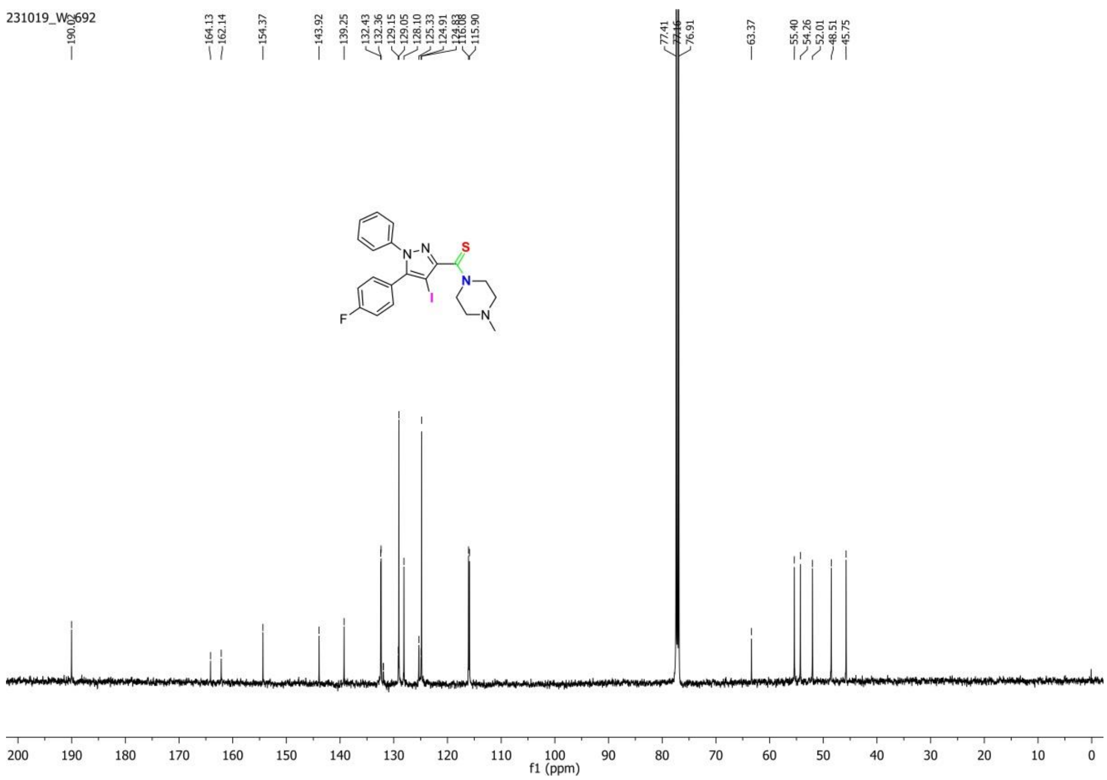

Figure S43: ^{13}C NMR spectrum of **11E**.

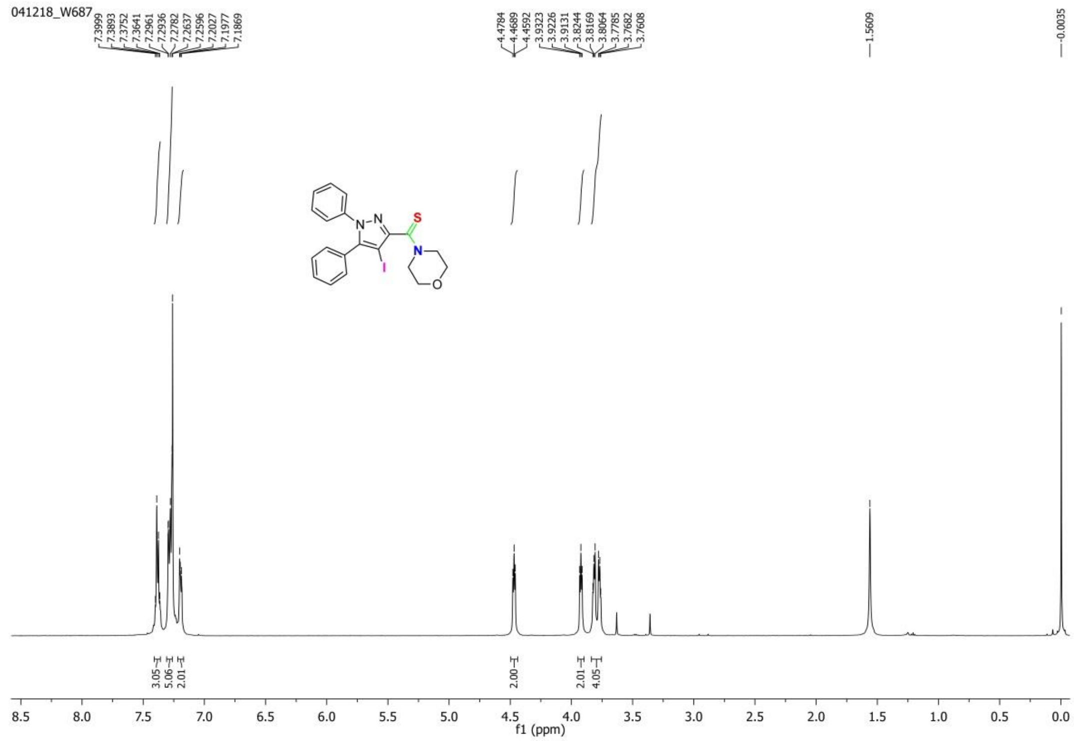

Figure S44: ^{1}H NMR spectrum of **12C**.

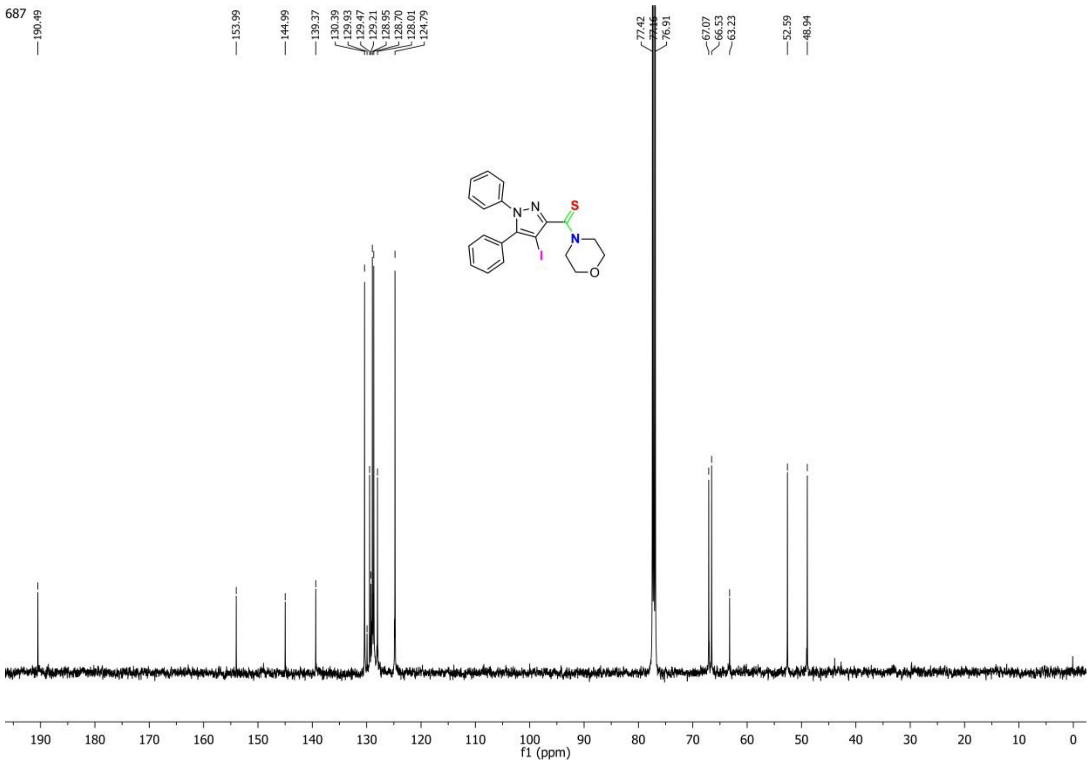

Figure S45: ^{13}C NMR spectrum of **12C**.

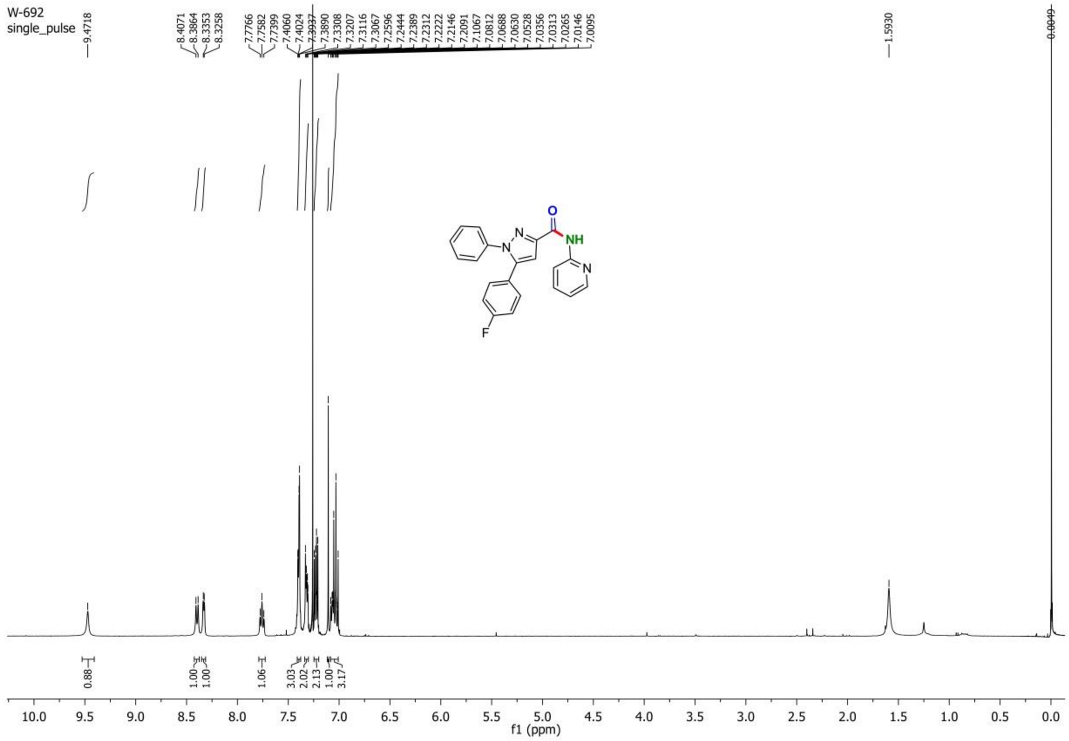

Figure S46: ^{1}H NMR spectrum of **1F**.

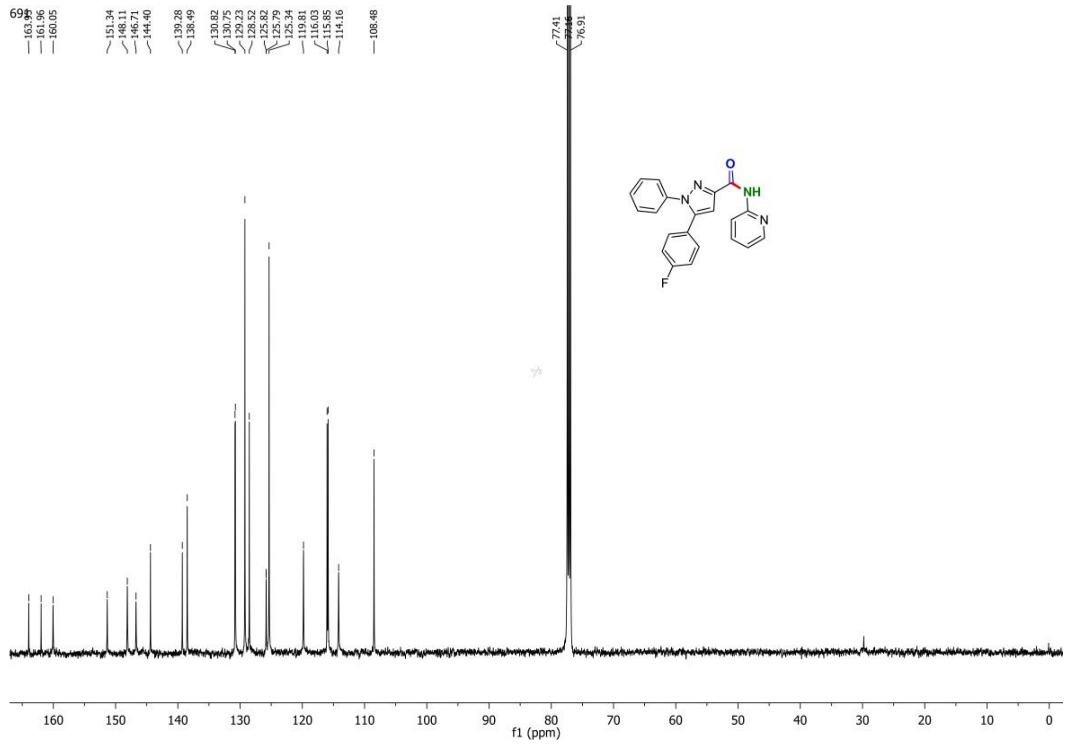

Figure S47: ^{13}C NMR spectrum of **1F**.

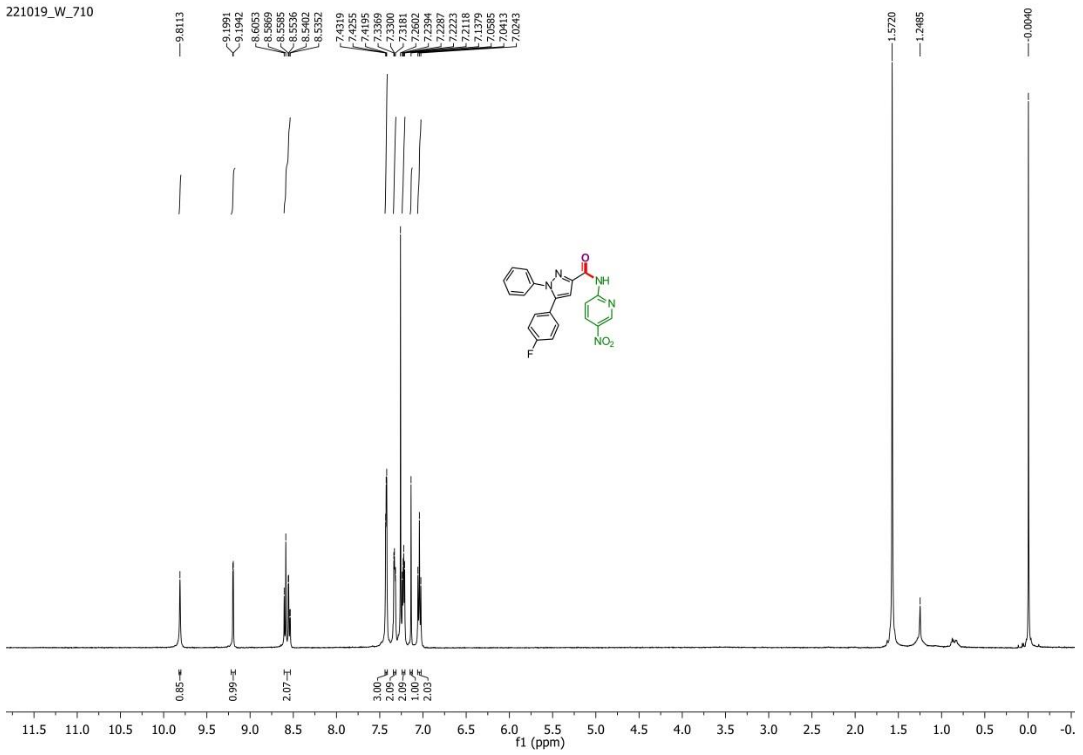

Figure S48: ^{1}H NMR spectrum of **1G**.

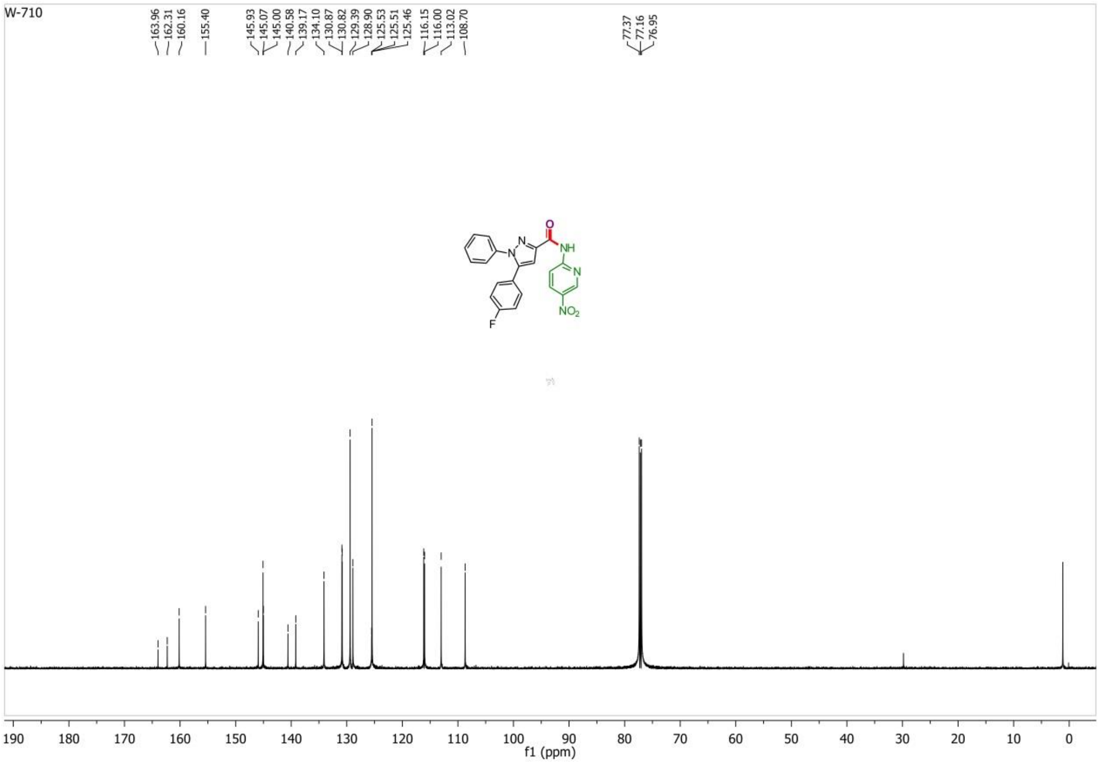

Figure S49: ^{13}C NMR spectrum of **1G**.

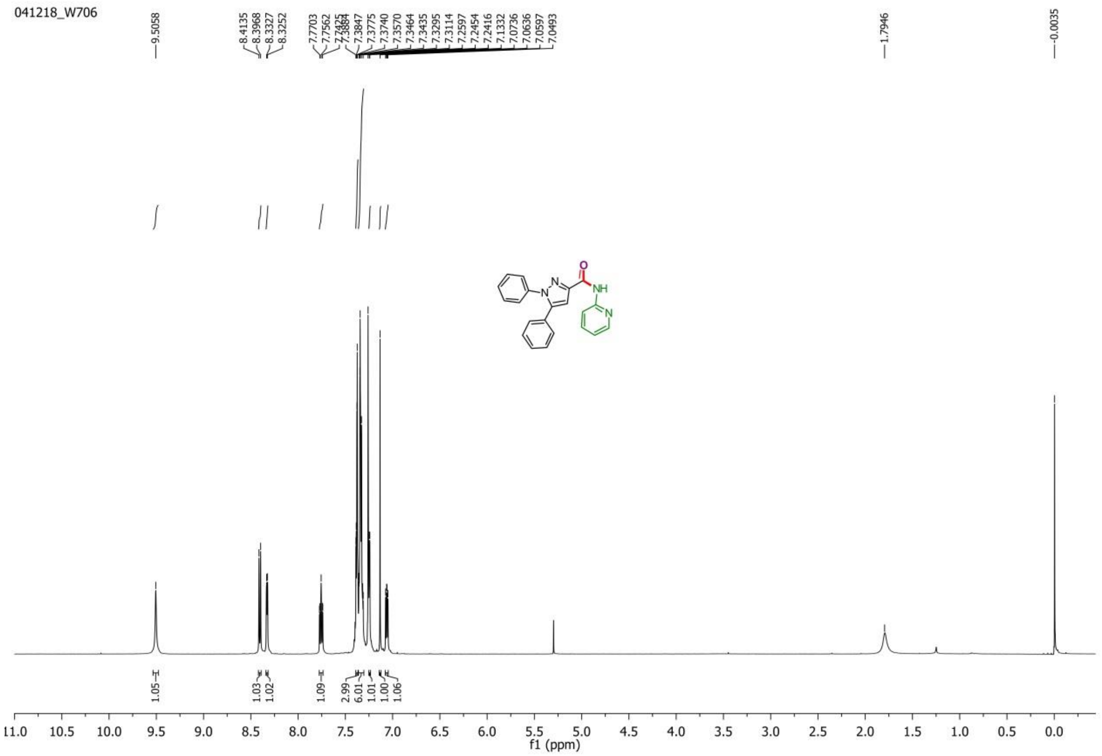

Figure S50: ^{1}H NMR spectrum of **4F**.

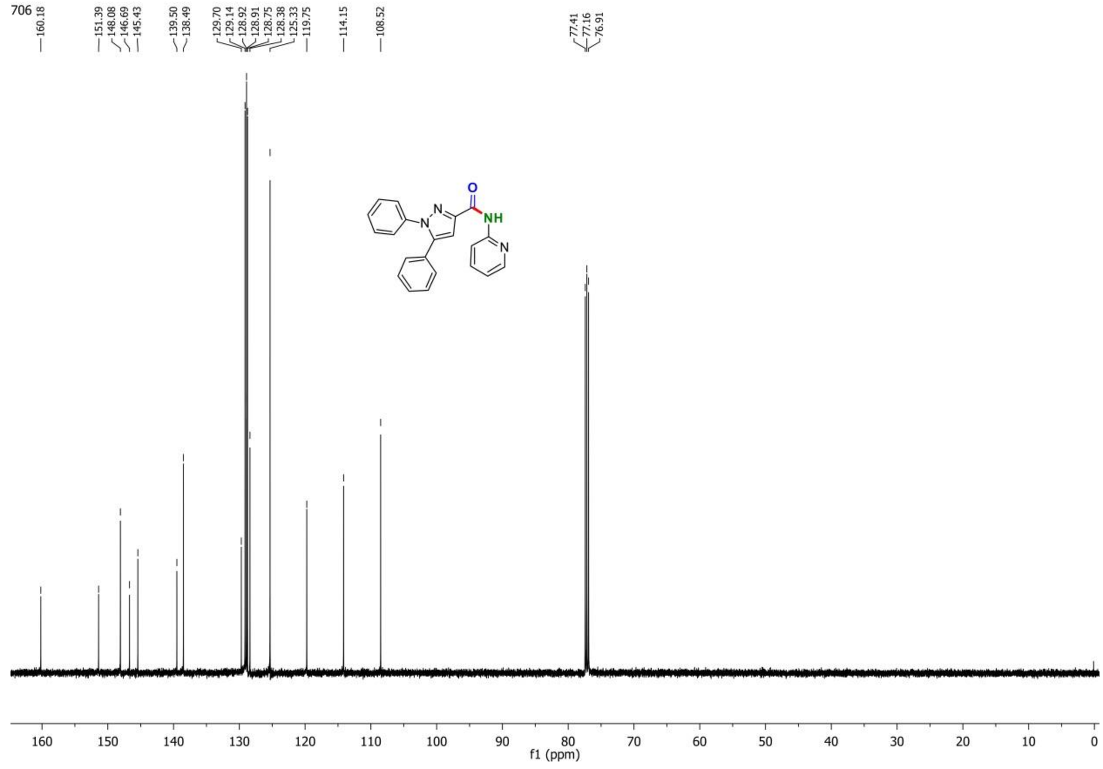

Figure S51: ^{13}C NMR spectrum of **4F**.

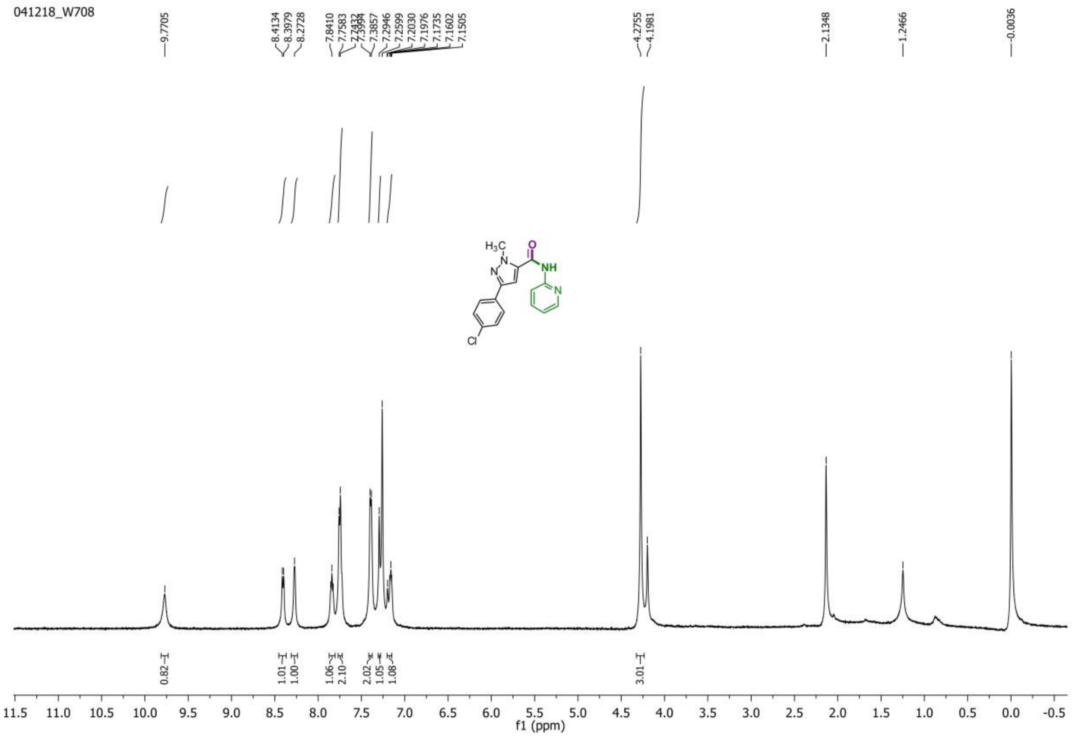

Figure S52: ^{1}H NMR spectrum of **9F**.

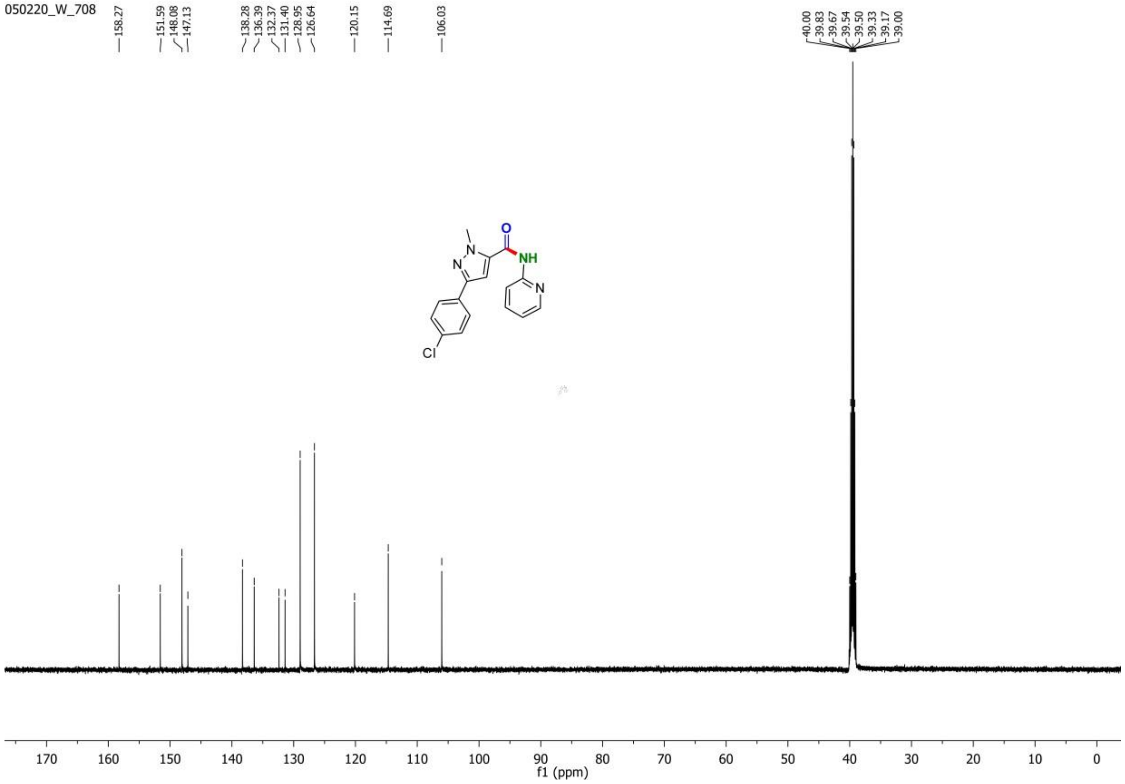

Figure S53: ^{13}C NMR spectrum of **9F**.

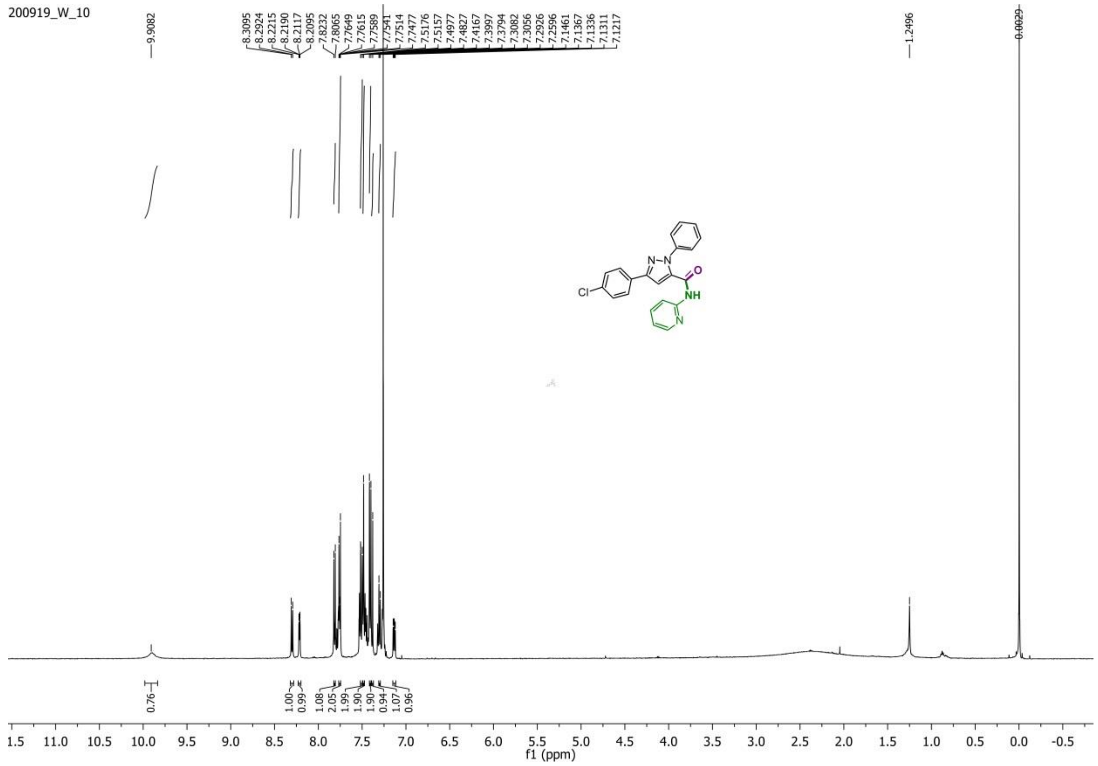

Figure S54: ^{1}H NMR spectrum of **10F**.

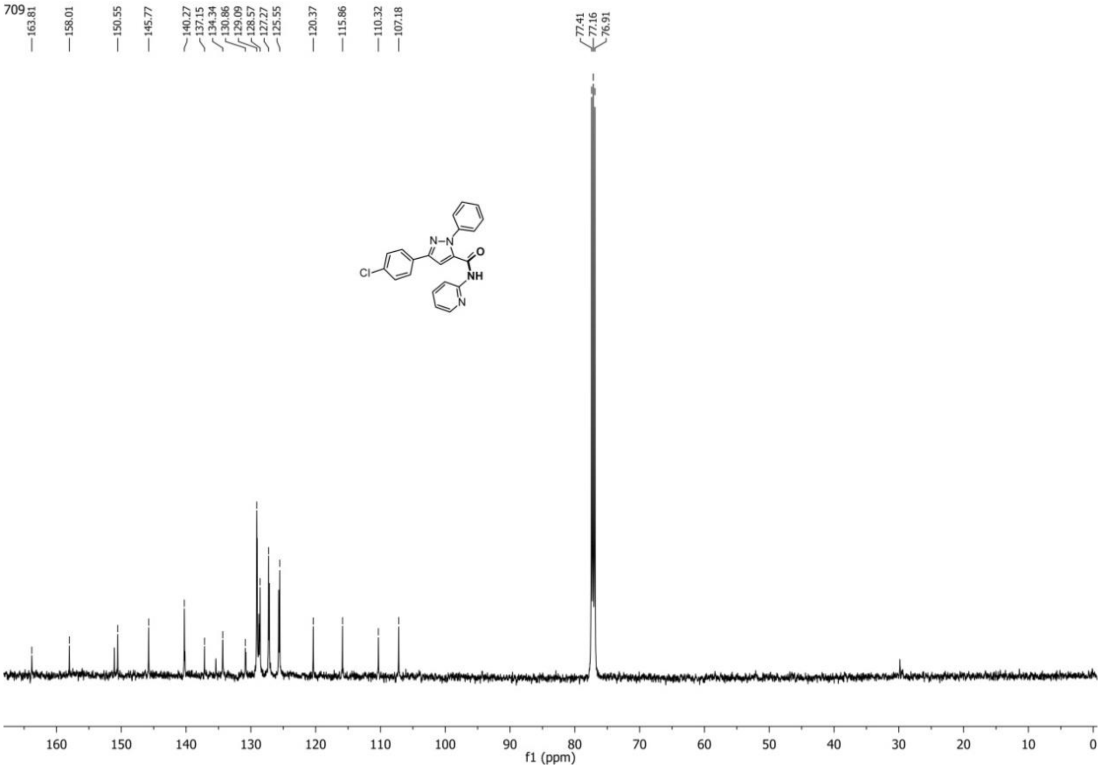

Figure S55: ^{13}C NMR spectrum of **10F**.
